# A pseudo-cubic metal–organic cage with conformationally switchable faces for dynamically adaptive guest encapsulation

**DOI:** 10.1038/s41557-024-01708-5

**Published:** 2025-01-08

**Authors:** Houyang Xu, Tanya K. Ronson, Andrew W. Heard, Paula C. P. Teeuwen, Laura Schneider, Philipp Pracht, John D. Thoburn, David J. Wales, Jonathan R. Nitschke

**Affiliations:** 1 Yusuf Hamied Department of Chemistry, University of Cambridge, Cambridge, UK; 2 Department of Chemistry and Chemical Biology, TU Dortmund University, Dortmund, Germany; 3 Randolph-Macon College, Department of Chemistry, Ashland, VA USA

**Keywords:** Molecular capsules, Self-assembly, Coordination chemistry

## Abstract

The creation of hosts capable of accommodating different guest molecules may enable these hosts to play useful roles in chemical purifications, among other applications. Metal–organic cages are excellent hosts for various guests, but they generally incorporate rigid structural units that hinder dynamic adaptation to specific guests. Here we report a conformationally adaptable pseudo-cubic cage that can dynamically increase its cavity volume to fit guests with differing sizes. This pseudo-cube incorporates a tetramine subcomponent with 2,6-naphthalene arms that cooperatively adopt a non-planar conformation, enabling the cage faces to switch between *endo* and *exo* states. A wide range of guest molecules were observed to bind within the cavity of this cage, spanning a range of sizes from 46% to 154% of the cavity volume of the empty cage. Experimental and computational evidence characterizes the flipping of cage faces from *endo* to *exo*, expanding the cavity upon binding of larger guests.

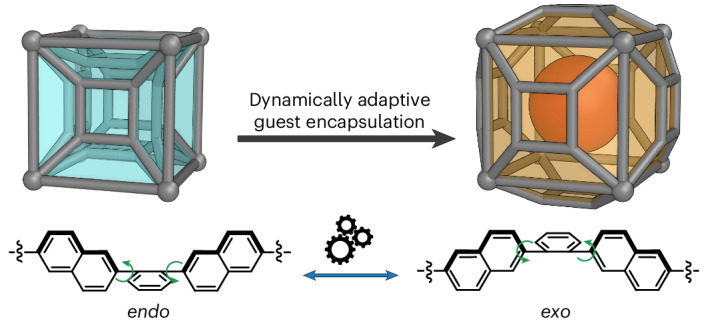

## Abstract (web summary)

Synthetic nanocages that can adapt the size and shape of their cavity in response to a given guest have potential applications in various areas, including chemical purification. Now a flexible, pseudo-cubic metal–organic cage has been developed that is able to dynamically expand its cavity from 46% to 154% of its initial volume by flipping its cage faces.

## Main

Biological molecules dynamically adapt their conformations to undertake their functions. For example, DNA supercoils enable the cell packaging and regulation of replication and transcription processes^1,2^. RNA adopts a loose chain configuration to transcribe information but folds to carry nucleic acids or to incorporate into ribosomes^3^, whereas proteins undergo conformational changes to bind ligands and catalyse or inhibit reactions^4,5^. The tremendous range of functions for these biomolecules thus depends on their ability to adapt their conformations.

Host–guest chemistry and molecular recognition likewise underpin a diverse array of functions in biology and chemistry^6–8^. Many artificial host molecules have been developed^9–14^, including polyhedral metal–organic cages, whose well-defined cavities serve as binding pockets^11,12^. Guest binding may be driven by solvophobic effects^15,16^, hydrogen bonding^17,18^, Coulombic interactions^19^, halogen bonding^20^ and arene stacking^21,22^. As described by Fischer’s lock-and-key principle^23^, size and shape complementarity between binding sites and guest molecules has a pivotal role in determining binding affinities^24–26^.

Enhancing the binding versatility of a supramolecular host can reduce the effort required to create a new receptor for a given guest molecule. More versatile hosts may be prepared by creating systems that involve variable numbers of assembling units^27^, incorporating structural adaptability through the introduction of mechanical bonds^28,29^, creating hosts with chemical switchability^30–32^ or imparting inherent adaptability to the structures^33–35^. In the field of metal–organic cages, the quest for cage designs that combine conformational flexibility with structural integrity remains an important challenge. In accordance with Koshland’s ‘induced fit’ theory, conformationally adaptable host molecules, with structural dynamics resembling those of proteins, may be able to reconfigure in the presence of different guests to optimize binding affinity^36^. However, the entropic demands associated with the assembly of metal–organic cages necessitate preorganization, highlighting the need to balance structural rigidity with adaptability^37^. Reported cases of metal–organic cages incorporating such adaptability are few, with strategies including the introduction of flexible ligands^35,38^, stereochemically adaptable vertices^39,40^ or geometrically flexible frameworks^41–44^. Here, we report the synthesis of a Zn_8_L_6_ pseudo-cubic cage with inherently non-planar faces, which can adopt *endo* or *exo* states. As each face can switch independently from *exo* to *endo*, the metal–organic cage possesses the capability to adjust the size and shape of its cavity in response to the specific molecules it binds, ensuring a versatile and optimized guest encapsulation. This work demonstrates that incorporating conformational flexibility into metal–organic cages is feasible while producing only a single discrete assembly and preventing the formation of coordination polymers, thus addressing an enduring challenge in the design of such adaptable systems.

## Results and discussion

**Fig. 1:**
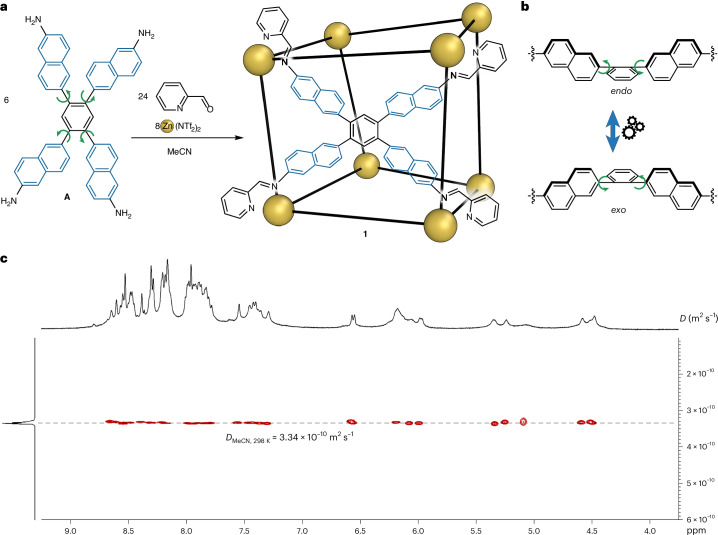
Preparation and characterization of cage 1. **a**, The synthesis of pseudo-cube **1** from tetramine subcomponent **A**. **b**, The two conformations of each face, as a result of the 2,6-naphthalyl rotational units. **c**, ^1^H NMR and ^1^H DOSY spectra of pseudo-cube **1**, revealing a diffusion coefficient (*D*) of 3.34 × 10^−10^ m^2^ s^−1^ (400 MHz, 298 K, CD_3_CN).

We sought to generate a conformationally adaptive cage by incorporating rotational subunits into the ligand arms. To implement this concept, the naphthalene-armed tetramine subcomponent **A** was designed and synthesized via a two-step route in 72% overall yield (Supplementary Scheme 1). Based on our previous work with rectangular tetramine subcomponents^43^, we hypothesized that subcomponent **A** would produce a pseudo-cubic cage with 2-formylpyridine and Zn^II^. The four 2,6-naphthyl groups panelling each face of the cage were designed to rotate so as to generate non-planar faces. This rotation could help in accommodating a guest, whereby each face may adopt an *exo* or *endo* configuration (Fig. 1a). The *exo* and *endo* ligand configurations are expected to have the same metal–metal distances, thus allowing each face to switch independently without introducing strain into the overall cage framework.

### Synthesis and characterization of pseudo-cube 1

**Fig. 2:**
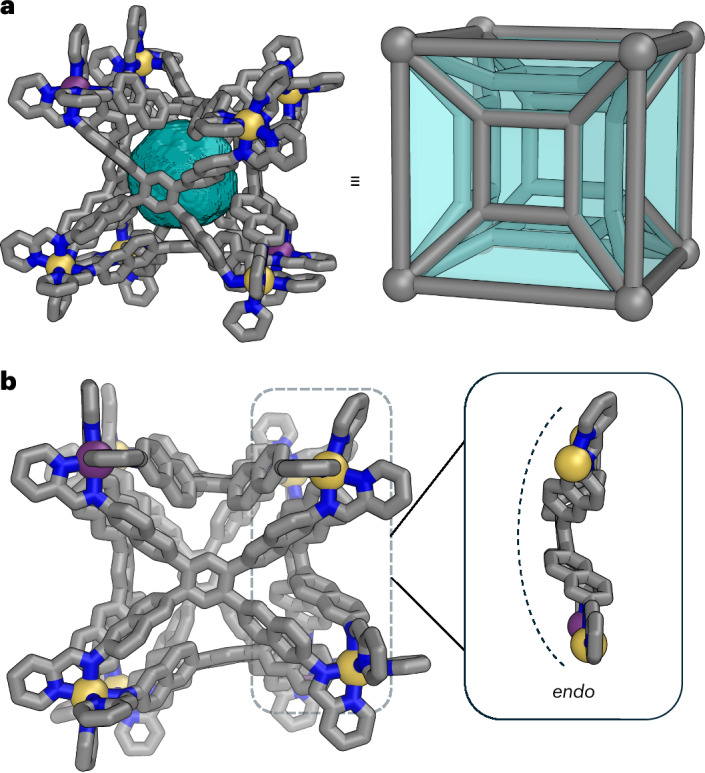
Single-crystal structure of cage **1**. **a**, Oblique view of the crystal structure of **1**, showing the Molovol^44^-calculated cavity in cyan and a cartoon representation of its all-*endo* conformation. **b**, Front view of the crystal structure, showing its all-*endo* conformation. C, grey; N, blue; *fac*-Δ-Zn, yellow; *fac*-Λ-Zn, purple. Hydrogens, counterions and solvent molecules have been omitted for clarity.

The self-assembly of tetramine subcomponent **A** (6 equiv.), zinc(II) bis(trifluoromethanesulfonyl)imide (triflimide, NTf_2_^−^) (8 equiv.) and 2-formylpyridine (24 equiv.) generated Zn^II^_8_L_6_ cage **1**, as confirmed by nuclear magnetic resonance (NMR) (Supplementary Figs. 1–16) and high-resolution electrospray ionization mass spectrometry (ESI-HRMS) experiments (Fig. 1b,c and Supplementary Fig. 17). After heating at 343 K overnight in CH_3_CN, all the ^1^H NMR signals of the product exhibited the same diffusion constant in the ^1^H diffusion-ordered spectroscopy (DOSY) NMR spectrum (Fig. 1c). Four sets of magnetically inequivalent ligand arm signals were observed (Supplementary Figs. 7–16), indicating the possible presence of a *C*_3_ symmetry axis, consistent with overall *D*_3_ or *S*_6_ point symmetry^45^. The ESI-HRMS spectrum of **1** indicated a Zn_8_L_6_(NTf_2_)_16_ composition (Supplementary Fig. 17). Single-crystal X-ray diffraction analysis confirmed the pseudo-cube structure of cage **1**, with *D*_3_ point symmetry (Fig. 2, Supplementary Fig. 18 and Supplementary Table 1). Both enantiomers are present in the crystal structure. In one (Fig. 2), two antipodal metal vertices exhibited Λ handedness, with the others adopting Δ stereochemistry. Each face is enclosed by a tetratopic pyridyl-imine ligand incorporating tetramine **A**, with short and long axes of the tetratopic ligand mismatching in 6 out of the 12 edges of the pseudo-cube.

The crystal structure of cage **1** verifies the presence of the non-planar facial conformations. As seen in Fig. 2, steric hindrance between neighbouring naphthyl units led to a configuration in which the central phenyl moieties of each face protrude towards the cage interior. We label this face conformation *endo*, and the alternative arrangement, where the central phenyl is projected outwards from the centre, *exo*. Because steric hindrance between naphthyl groups precludes a face from adopting a planar conformation, each face of **1** may thus be assigned to either an *endo* or *exo* conformation.

Entropic factors may account for the all-*endo* initial facial conformation in the case of empty **1**. The all-*endo* conformation would minimize the cavity volume when no guest is bound, thereby releasing the maximum number of solvent molecules into solution. The six *endo* faces of **1** were found to enclose a single central cavity (Fig. 2a) with a volume of 389 Å^3^, as calculated using MoloVol^46^ (Supplementary Fig. 19). We infer that the dynamic switching of these faces between the two conformations has a key role in allowing **1** to bind a wider range of guests than would otherwise be possible, as discussed below.

### Guest binding studies of 1

Host–guest studies revealed that **1** accommodated neutral and anionic guests of varying sizes (Fig. 3). The volumes of these guest molecules have been sphericity-corrected, spanning a range from 178 Å^3^ for adamantane to 599 Å^3^ for *tetrakis*(4-chlorophenyl)borate. This nearly fourfold volume difference between the smallest and largest guest encapsulated by **1** is remarkable compared with previous reports on flexible cages^34,35,37,47^. The addition of any of the guests shown in Fig. 3 to a solution of the cage resulted in either a shift of all ^1^H NMR signals or the emergence of at least one new set of signals for **1** (Supplementary Figs. 20–34). For all neutral guests, ESI-HRMS supported a 1:1 binding ratio, with distinct [H + G] peaks observed (Supplementary Figs. 35–47). No evidence for other binding ratios was observed. For the tetraarylborates, ^1^H NMR titrations suggested a 1:1 binding stoichiometry (Supplementary Figs. 48–53).

**Fig. 3:**
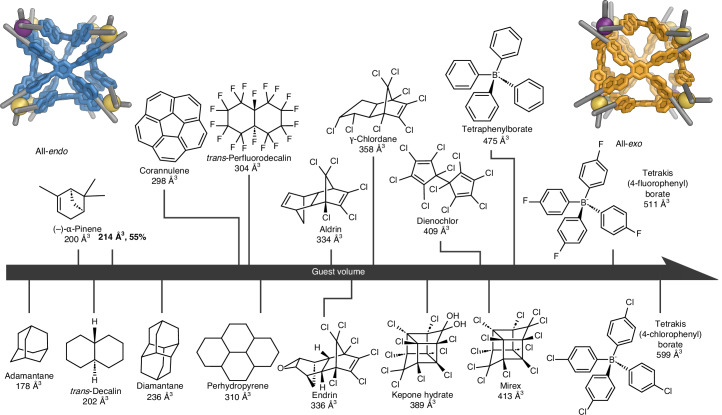
The range of guest molecules used in this study, all of which exhibited 1:1 binding with **1**. The sphericity-corrected volumes of guests ranged from 178 to 599 Å^3^ (summarized in Supplementary Table 2), with larger guests flipping more faces of **1** from *endo* to *exo* during dynamically adaptive guest encapsulation. The all-*endo* structure here used for illustration is the single-crystal X-ray diffraction structure of 1. The all-*exo* structure is a structure of B(*p*-Cl-C_6_H_4_)^−^⊂**1** optimized with GFN2-xTB^53^, which gave six *exo* faces in the minimization. C: grey, blue (for *endo* faces) or orange (for *exo* faces); *fac*-Δ-Zn: yellow; *fac*-Λ-Zn: purple. The host hydrogen atoms in both conformations and the guest in the all-*exo* structure are omitted, and the vertices including the imine-pyridyl ligand moieties have been simplified, for clarity.

In accordance with Rebek’s rule^48^, optimal binding with **1** would correspond to a guest molecule with a volume of approximately 214 Å^3^, which corresponds to 55% of the cavity volume of **1** in the crystal. Many of the guests shown in Fig. 3 exceed this volume, with the largest guest having a volume of 599 Å^3^, corresponding to 154% of the initial cavity volume of **1**.

### Conformational transformation of 1 upon guest binding

During investigations of guest binding within **1**, in most cases, only one set of guest peaks was observed, implying fast guest exchange on the NMR timescale (Supplementary Fig. 20). However, we noted splitting of the cage signals into at least two sets, consistent with different environments in slow exchange on the NMR timescale. One host set corresponds to the original signals of the empty cage, whereas the other emerges upon guest addition and gradually intensifies over time. Since only a single set of shifted ^1^H NMR signals was observed for the guest molecule, we infer that the cage undergoes a structural reconfiguration, slow on the NMR timescale, allowing it to optimise its conformation to accommodate the guest. Directly studying the conformations of the cage using ¹H NMR in these cases proved challenging, so we turned to alternative techniques to elucidate these structural changes.

**Fig. 4:**
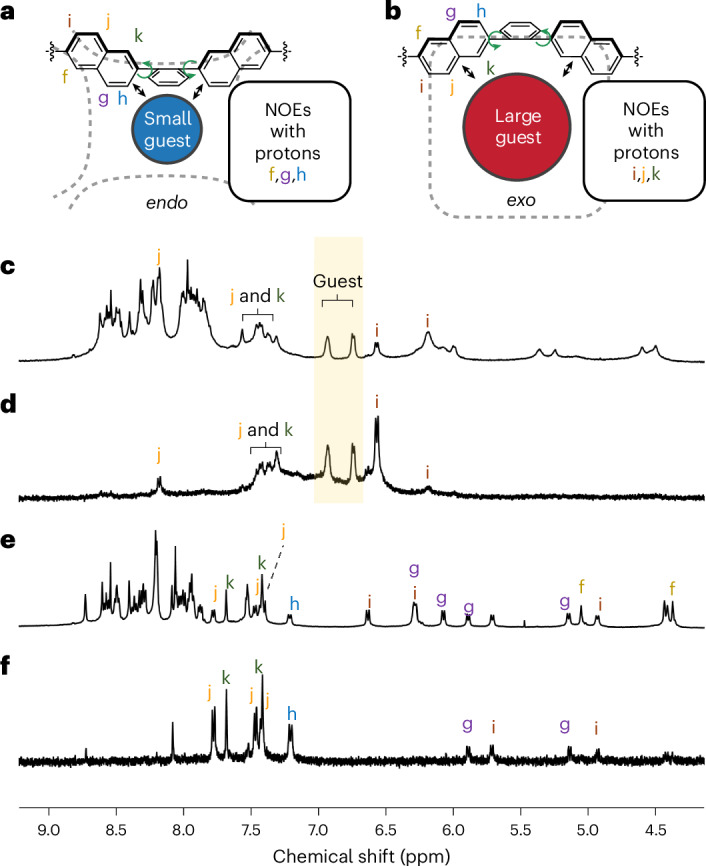
One-dimensional (1D) NOESY spectra of host–guest complexes of **1**, showing the host conformational changes upon guest binding. **a**,**b**, Illustration of different naphthalene protons to show NOEs between guest molecules and host panels in *endo* (**a**) versus *exo* (**b**) configurations: When a small guest is bound in proximity to an *endo* panel, protons *f*, *g* and *h* will exhibit NOEs with the guest. Larger guests will show NOEs with protons *i*, *j* and *k* with an *exo* host panel. **c**,**d**, ^1^H NMR spectrum (**c**) and 1D selective gradient NOESY spectrum (irradiated as highlighted at 7.03–6.69 ppm) (**d**) of B(*p*-Cl-C_6_H_4_) ^−^⊂**1**. **e**,**f**, ^1^H NMR spectrum (**e**) and 1D selective gradient NOESY spectrum (irradiated at 2.08–2.00 ppm) (**f**) of diamantane⊂**1**.

As our attempts to obtain X-ray-quality crystals of the host–guest complexes of **1** were not successful, we turned to ^1^H-^1^H nuclear Overhauser effect spectroscopy (NOESY) measurements to probe the conformations of the guest⊂**1** complexes in solution. As shown in Fig. 4a,b, selectively irradiating at frequencies corresponding to guest signals revealed which host protons were in closest proximity to the guest. When a face is in an *endo* conformation, the *f*, *g* and *h* protons point to the interior of the cavity and will thus exhibit nuclear Overhauser effects (NOEs) with the guest, as observed during smaller guest binding. By contrast, *exo* faces have the *j*, *k* and *i* protons pointing inwards. These protons are thus adjacent to the guest when larger guests are bound. When the *tetrakis*(4-chlorophenyl)borate anion was bound, its phenylene protons showed NOEs with protons *j* and *k* (Fig. 4c,d). Minor NOE signals were also observed with protons *g* and *i*. No NOEs were observed between the guest and any imino-pyridine protons. We do not anticipate that the large *tetrakis*(4-chlorophenyl)borate anion could bind to the exterior of the cage without being in proximity to these protons. Hence, all these observations support the hypothesis that the cage face conformations switch to *exo* to accommodate this large anion within the cavity.

In the case of diamantane (Fig. 4e,f), the observed NOEs suggest spatial proximity between the guest molecule and both sides of the host naphthalenes, indicating that **1** may adopt different conformations featuring both *endo* and *exo* faces to accommodate diamantane. Given the number of signals present, we infer that the multiple conformations are probably in fast exchange due to the structural flexibility of the cage faces. The integral ratios between outward-facing and inward-facing naphthalene signals (Supplementary Fig. 54) suggest that more *exo* than *endo* faces may be present in these conformations, provided that the distances between the naphthalene protons and the guest remain comparable.

Differing NOE correlations between the three ligand arms that meet at each vertex also support the assignment of *exo* ligand conformations following the binding of large guests within **1** Supplementary Fig. 55). The *f* protons from different ligand arms showed cross peaks in the NOESY spectrum of empty **1** (Supplementary Fig. 56), whereas, following the binding of larger guests, such as diamantane (Supplementary Figs. 57–59) and *trans*-perfluorodecalin (Supplementary Figs. 60–62), fewer inter-*f* cross peaks were found, in favour of more *f*–*i* cross peaks, consistent with more naphthalenes adopting the conformation required in *exo* ligand faces (Fig. 4b).

### Expansion of 1 upon guest binding

Increasing numbers of *exo* faces should result in an increase in the measured size of **1**. A series of ion mobility mass spectrometry (IMS) (Supplementary Figs. 63–85) and ^1^H DOSY experiments (Supplementary Figs. 87–97) were therefore carried out to gauge the increasing size of **1** upon guest binding. As shown in Fig. 5a, cage **1** generates a characteristic series of ions that travel with different drift time. As the collisional cross-section (CCS) value is proportional to the drift time, slower ions, with lower charges, provide the most sensitive means of probing CCS. We thus chose the guest adducts of the [**1**·(NTf_2_)_11_]^5+^ ion, which was the smallest-charged ion that was consistently observed, for analysis of CCS values. Figure 5b shows mobilograms of the 5+ ions of **1** and its host–guest complexes, showing that guest binding results in variable increases in drift time.

**Fig. 5:**
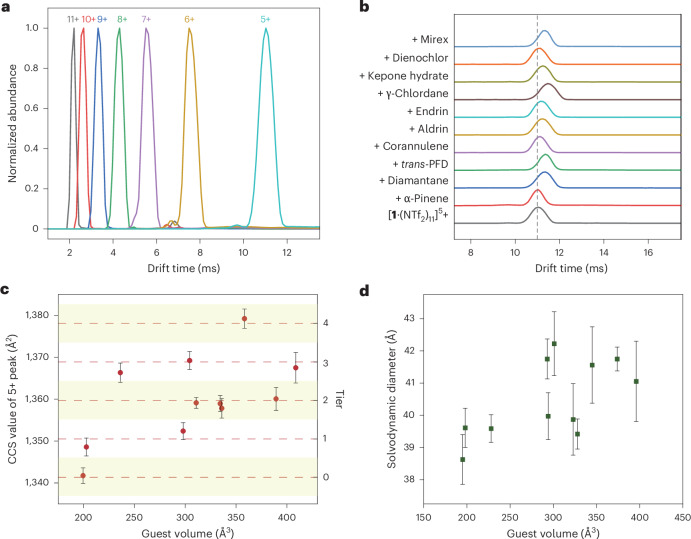
Mass spectrometry and NMR reveal how cage **1** increases its size in discrete increments upon binding progressively larger guests. **a**, Normalized mobilograms of ions corresponding to cage **1** with varying numbers of counterions. **b**,**c**, Stacked, normalized mobilograms (**b**) and CCS values (**c**) of the [**1**·(NTf_2_)_11_]^5+^ ion from cage **1** and its host–guest complexes, showing how larger guests lead to larger CCS values that cluster into tiers, which we infer to correspond to increasing numbers of *exo* cage faces. Guest volumes are corrected for sphericity. The data are presented as mean values ± standard deviation from the CCS values of ~1,000–3,000 distinct ion peaks, with details provided in Supplementary Table 3 and Supplementary Information section 5.4. **d**, The solvodynamic diameter of cage **1** and its various host–guest complexes from ^1^H DOSY data. The data are presented as mean values ± standard deviation from the solvodynamic diameters, calculated from ~20–40 individual NMR signals with the Stokes–Einstein equation. For details, please see Supplementary Table 4 and Supplementary Information section 5.5. Source data

CCS values obtained using the Mason–Schamp equation^49–51^ are summarized in Fig. 5c and Supplementary Table 3, where they are plotted against the guest volumes. Examination of the CCS values suggested that they cluster into five tiers. We thus fitted the CCS values to the linear expression

1 $${{\rm{CCS}}}_{{\rm{guest}}\subset \mathbf{1}}={{\rm{CCS}}}_{{\rm{guest}}\subset \mathbf{1}}^{0}+n\times \Delta {\rm{CCS}},$$

where ${{\rm{CCS}}}_{{\rm{guest}}\subset 1}^{0}$ and ΔCCS stand for the smallest observed CCS value of guest⊂**1** and the tier increment, respectively. Each tier of data points (highlighted with horizontal stripes in Fig. 5c) was assigned a tier number *n* from 0 to 4. Linear least-squares fitting of the CCS values using equation (1) provided a tier increment ∆CCS of 9.2 Å^2^ and a smallest CCS guest⊂**1** value of 1,341 Å^2^ (Supplementary Fig. 86), with an *R*^2^ of 0.997. The quality of fit obtained suggests that the CCS values may describe a quantized system, where a discrete number of cage faces become *exo* during adaptation to the size and shape of each specific guest.

The corresponding ^1^H DOSY results are shown in Fig. 5d. The solvodynamic diameters, calculated via the Stokes–Einstein equation, also exhibited a positive correlation with increasing guest size, but the values are distributed more evenly than the CCS values.

The different conditions probed by the two methods may account for the different results obtained. As ions desolvate during IMS measurements, van der Waals interactions between proximate naphthyl groups would be maximized during the formation of a structure with the minimum number of *exo* faces to accommodate a given guest. These internal solvation effects^52^ are thus anticipated to favour a single conformation with a discrete number of *endo* faces and a correspondingly well-defined CCS. In contrast, in the liquid phase of the DOSY measurements, acetonitrile solvation may stabilize host–guest complex microstates with varying *exo*–*endo* face ratios simultaneously. As these microstates equilibrate in fast exchange on the NMR timescale (Supplementary Fig. 20–34), DOSY would be expected to report a diffusion rate intermediate between them.

### Possible conformations and cavity states of 1

**Fig. 6:**
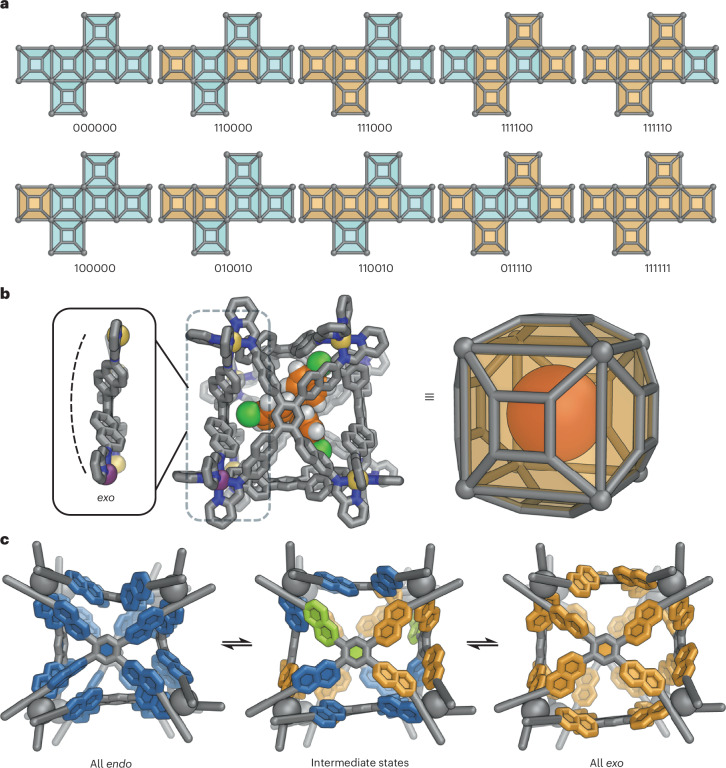
Illustration of possible conformations for cage **1**. **a**, Enumeration of the ten possible states that cage **1** can adopt, with differing face conformations. A blue colour and ‘0’ denote *endo* faces, while orange and ‘1’ represent *exo* faces. **b**, The GFN2-xTB^53^ model of B(*p*-Cl-C_6_H_4_)^−^⊂**1**, minimised to give six *exo* faces (111111 in **a** above, ‘all-*exo*’ in **c** below). C, grey or orange (guest); N, blue; *fac*-Δ-Zn, yellow orange; *fac*-Λ-Zn, purple; Cl, green; H, white. Hydrogen atoms of the cage are omitted for clarity. **c**, An illustration of the pathway for conversion between the all-*endo* to the all-*exo* conformation of **1**, calculated with the GFN-FF^54^ potential and computational tools from the energy landscape framework (Supplementary Video 1). The metal vertices are simplified for clarity. Blue, lime and orange denotes *endo*, intermediate and *exo* states of the structure, respectively.

On the basis of molecular modelling, we hypothesized that each face of **1** has local minima corresponding to *endo* and *exo* configurations, with all of the conformations shown in Fig. 6a potentially accessible. We also inferred that the energetic difference between these conformations would be considerably less than the energy of guest binding. This view of the guest-binding behaviour of **1** was consistent with the CCS values obtained (Fig. 5c), in which five of the cavity volume states may represent the five tiers found in the plot. However, ^1^H NMR spectra of the host–guest complexes do not show clear-cut evidence of cage desymmetrization, suggesting that the conversion between different cage conformations occurs in fast exchange. Figure 6b and Supplementary Fig. 98 shows an GFN2-xTB^53^-optimized structure of B(*p*-Cl-C_6_H_4_)^−^⊂**1**, which suggests that **1** expands its cavity by adopting an all-*exo* conformation to accommodate this large anionic guest.

To further understand the conversion process between the bistable states of the faces of **1**, we conducted a simulation of the transition of **1** from fully *endo* to fully *exo*. The crystal structure of empty **1** and a modelled structure of B(*p*-Cl-C_6_H_4_)^−^⊂**1** were used to define the starting and end points, and were optimized with the GFN-FF method^54^ before the simulation. A 40-step path, with 40 transition states between the two states, was found and is visualized in Fig. 6c and Supplementary Video 1. This pathway suggests that the naphthyls can rotate individually and, in some steps, concertedly during the transition. These rotations expand the cavity of the cage in response to the presence of guest molecules and help optimize the host to provide the best fit in each case. The energy landscape for this pathway is summarized in a disconnectivity graph^55,56^ and an energy profile based on all the configurations identified along the pathway (Supplementary Figs. 99 and 100 and Supplementary Table 5).

Our computational results suggested that the *endo* and *exo* face configurations of **1** are nearly isoenergetic. Although the results from the methods we used, especially force field (FF) methods, may exhibit some degree of uncertainty, they still provide valuable insights into the energetic differences between the various conformations of **1**. The fully *exo* conformation of **1** is lower in energy than the fully *endo* conformation of **1** when calculated with the GFN-FF method, but higher in energy using density functional theory (DFT) at the r^2^SCAN-3c level^57^. When an implicit acetonitrile solvation model was applied, the fully *exo* structure became energetically comparable to the *endo* starting point. With our experimental observations in mind, namely that certain guests drive the cage towards *exo* conformations, such subtle differences in the energies underscore the importance of suitable guest molecules in triggering the conformational change of **1**. The comparison of energy landscapes calculated with DFT and GFN-FF methods also indicates the important role of aromatic stacking interactions in the energy differences among the conformations. Whereas DFT calculations attempt to capture these interactions, FF methods are more likely to neglect them. Therefore, the difference in the energy landscapes calculated with different methods indicates that *endo* conformations are more favourable when the cavity is empty, while the presence of a guest would disrupt the stacking interactions, thereby destabilizing the *endo* conformations and pushing the cage towards *exo* structures.

GFN-FF produces a highest-energy barrier of 34.9 kJ mol^−1^ for the all-*endo* to all-*exo* conversion pathway, versus 58.9 kJ mol^−1^ using r^2^SCAN-3c. Estimates from variable-temperature NMR results (Supplementary Figs. 101–104 and Supplementary Tables 6 and 7) suggested an energy barrier of 4–9 kJ mol^−1^ for the structural conversion, lower than the calculated barriers. As the calculations were performed using the empty cage structure, the observed difference suggests that guests reduce these barriers through non-covalent interactions and facilitate the structural transformation of **1** by stabilizing higher-energy intermediate structures, accounting for the fast NMR exchange between conformations of **1** at room temperature. We also note that entropic effects and intermolecular interactions from guest binding have not been considered in the calculations. The mechanistic characteristics of the interconversion process are the main focus of interest in the simulations. By complementing our experimental observations, these computational results provide a more comprehensive understanding of the structural dynamics of **1**.

## Conclusion

The introduction of 2,6-naphthalene rotating struts into tetramine subcomponent **A** enables the resulting pseudo-cube **1** to bind guest molecules with a wide range of sizes and shapes. From an initial all-*endo* conformation, the ability of the cage panels to adopt *exo* conformations allows **1** to bind guest molecules ranging from 178 to 599 Å^3^. Our results thus allow us to infer that **1** can adopt ten volume-quantized states through face reconfigurations, leading to seven accessible cavity volumes. This approach of integrating conformationally switchable faces into coordination cages may be generalizable across different classes of metal–organic cage, enabling augmentation of their versatility as molecular receptors.

## Online content

Any methods, additional references, Nature Portfolio reporting summaries, source data, extended data, supplementary information, acknowledgements, peer review information; details of author contributions and competing interests; and statements of data and code availability are available at 10.1038/s41557-024-01708-5.

## Supplementary information

Supplementary Information Supplementary Figs. 1–104, Tables 1–7 and Schemes 1 and 2.

Supplementary Data 1 Atom coordinates for the optimized structure shown in Fig. 6b.

Supplementary Data 2 Atom coordinates for the optimized pathway shown in Fig. 6c.

Supplementary Data 3 Single-crystal XRD data of empty cage **1** (CCDC 2367409).

Supplementary Video 1 A multimedia representation of the optimized pathway shown in Fig. 6c.

## Source data

Source Data Fig. 5 Source data for Fig. 5c (IMS) and Fig. 5d (^1^H DOSY).

## Data Availability

The authors declare that all data supporting the findings of this study are included within the Article and its Supplementary Information and are also available from the authors upon request. Crystallographic data for the structures reported in this paper have been deposited at the Cambridge Crystallographic Data Centre under deposition number 2367409 (empty cage **1**). Copies of these data can be obtained free of charge via www.ccdc.cam.ac.uk/data_request/cif. Source data are provided with this paper.
