# Supplementary material for: A pseudo-cubic metal–organic cage with conformationally switchable faces for dynamically adaptive guest encapsulation
Source: Nat Chem. 2025 Jan 8;17(2):289–96. doi: 10.1038/s41557-024-01708-5 (PMC11794150; doi:10.1038/s41557-024-01708-5)
Supplement: Supplementary file 1 — Supplementary Figs. 1–104, Tables 1–7 and Schemes 1 and 2. [file 41557_2024_1708_MOESM1_ESM.pdf]

nature chemistry

Supplementary information

https://doi.org/10.1038/s41557-024-01708-5

A pseudo-cubic metal–organic cage with conformationally switchable faces for dynamically adaptive guest encapsulation

In the format provided by the authors and unedited

Table of Contents

1 General Information S2
2 Synthesis and characterization S3
2.1 Subcomponent **A** S3
2.2 Pseudo cube **1** S9
3 X-ray crystallography S18
4 Volume calculation S22
5 Guest binding studies S25
5.1 Neutral guests S25
5.2 Anionic guests S47
5.3 Further NMR evidence of the face-flipping of **1** upon guest binding S53
5.4 Ion mobility mass spectrometry (IMS) studies S59
5.5 DOSY spectra S74
6 Computational studies S82
6.1 Structure modelling of B(*p*-Cl-C_6H_4)$^-$⊂**1** S82
6.2 Structural transformation simulations S82
6.3 Comparison of the energy barrier for the structural conversion of **1** S87
7 References S94

1 General Information

Unless specified differently, all chemicals were purchased from commercial suppliers and used without further purification unless stated otherwise. NMR spectra were recorded using NMR spectrometers including: Bruker DRX-400, Bruker Avance 500 Cryo, Bruker 500 TCI-ATM Cryo, 500 MHz AVIII HD Smart Probe, and Bruker 700 TCI-ATM Cryo. Chemical shifts (δ) for ^{1}H NMR spectra are reported in parts per million (ppm) and are reported relative to the solvent residual peak.

Signal multiplicity in ^{1}H and ^{13}C NMR spectra were reported with the following abbreviations: singlet (s), doublet (d), triplet (t), doublet of doublets (dd), triplet of doublets (td), doublet of doublet of doublets (ddd), multiplet (m), broad (br) and apparent (app.). HSQC spectra were recorded with edited pulse sequence, rendering CH/CH_3 and CH_2 cross peaks with opposite phases. All ^{1}H DOSY experiments were run on a Bruker 400 MHz Avance III HD Smart Probe spectrometer. The maximum gradient strength was 5.35 G/cm A. These experiments were run with a standard Bruker pulse program, ledbpgp2s, employing a stimulated echo and longitudinal eddy-current delay (LED) using bipolar gradient pulses for diffusion using 2 spoil gradients. Rectangular gradients were used with a total duration of 1.5 ms. A gradient ramp of 10% or 90% were utilized. Gradient recovery delays were 1100 µs. Data were processed with Bruker Dynamic Center 2.8.0.1 with diffusion coefficients calculated as the mean value for all the peaks identified. 1D NOESY experiments were run on a 500 MHz AVIII HD Smart Probe spectrometer using selective refocusing with a shaped pulse.

All high-resolution electrospray ionization mass spectra (ESI-HRMS) in this study were recorded using a Waters Synapt G2-Si instrument.

2 Synthesis and characterization

2.1 Subcomponent A

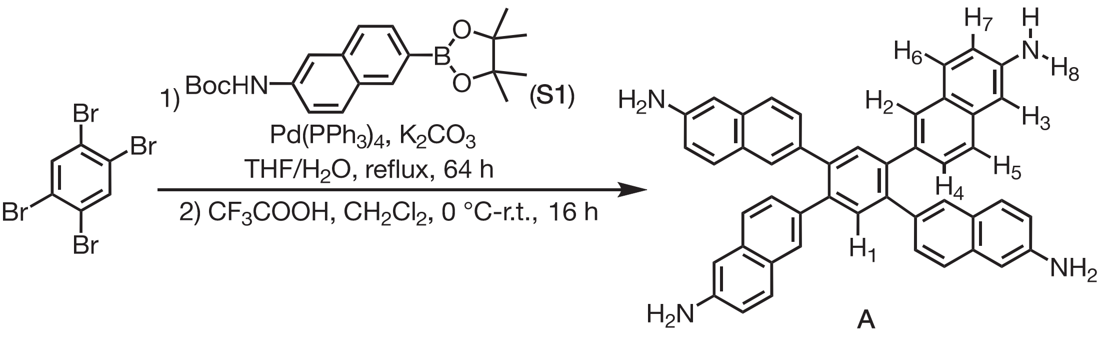

Supplementary Scheme 1. The synthetic route of subcomponent **A**.

S1 (Tert-butyl (6-(4,4,5,5-tetramethyl-1,3,2-dioxaborolan-2-yl)naphthalen-2-yl)carbamate) was synthesized from commercially available 2-amino-6-bromonaphthalene via a reported method.[1]

1,2,4,5-tetrabromobenzene (400 mg, 1.02 mmol, 1.00 equiv.), **S1** (2.06 g, 5.58 mmol, 5.5 equiv), and potassium carbonate (1.40 g, 10.2 mmol, 10.0 equiv) were added to a Schlenk flask. A mixture of THF/H_2O (4:1, 125 mL) was added to the flask and the resulting solution was bubbled with N_2 for 15 min. Palladium *tetrakis* (triphenylphosphine) (117 mg, 0.102 mmol, 0.1 equiv) was then added, before the mixture was heated at reflux for 64 h under nitrogen. After cooling to room temperature, the mixture was concentrated in *vacuo*, extracted with dichloromethane (100 mL × 5), dried over $MgSO_4$, and concentrated in *vacuo* again. A mixture of methanol / diethyl ether (1:6, 40 mL) was added to the resulting solid. The suspension was sonicated and then centrifuged. The supernatant was removed and the same process was repeated to the precipitate 5 times. The resulting solid was suspended in dichloromethane (10 mL), cooled to 0 °C, and 2 mL of trifluoroacetic acid was added. The mixture was stirred at room temperature for 16 h. The mixture was concentrated in *vacuo*, diluted in saturated sodium bicarbonate solution (50 mL), and filtered under reduced pressure. The residue was washed with dichloromethane/petroleum ether (1:6, 42 mL × 6), dried under *vacuo*, and was obtained as the product as a yellowish gray powder. Yield: (472 mg, 72% over 2 steps).

^{1}H NMR (500 MHz, DMSO-*d*$_6$, 298 K): δ = 7.72 (s, 4H), 7.58 (s, 2H), 7.51 (d, 4H, J = 8.8 Hz), 7.25 (d, 4H, J = 8.5 Hz), 7.00 (dd, 4H, $J1$ = 8.6 Hz, $J2$ = 1.5 Hz), 6.88 (dd, 4H, $J1$ = 8.6 Hz, $J2$ = 2.0 Hz), 6.72 (d, 4H, J = 1.6 Hz), 5.37 (s, 8H).

^{13}C NMR (126 MHz, DMSO-*d*$_6$, 298 K): δ = 146.9, 139.0, 133.7, 133.6, 133.2, 128.8, 128.0, 127.7, 126.4, 124.4, 118.4, 105.5.

ESI-HRMS calcd m/z = 643.2856 for $[C_{46}H_{35}N_4]^+$, found m/z = 643.2837.

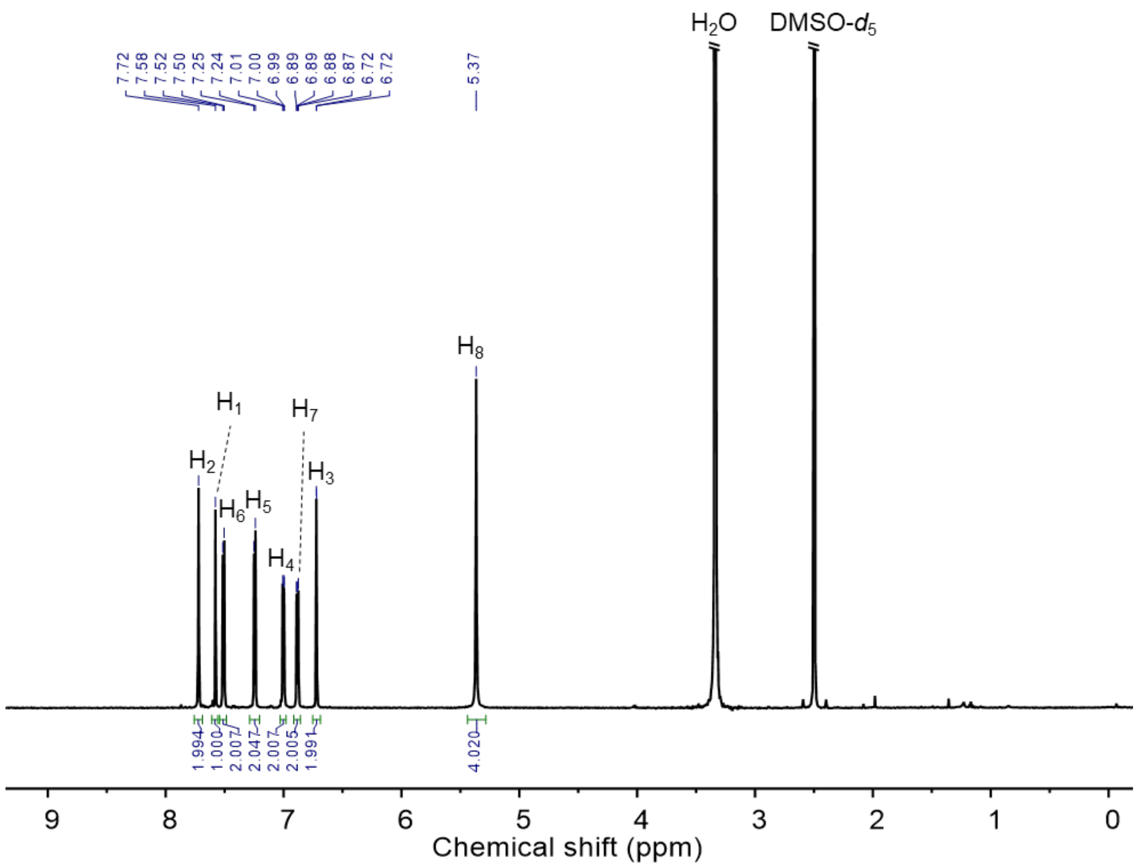

Supplementary Figure 1. ^{1}H NMR Spectrum (500 MHz, DMSO-*d*$_6$, 298K) of subcomponent **A.**

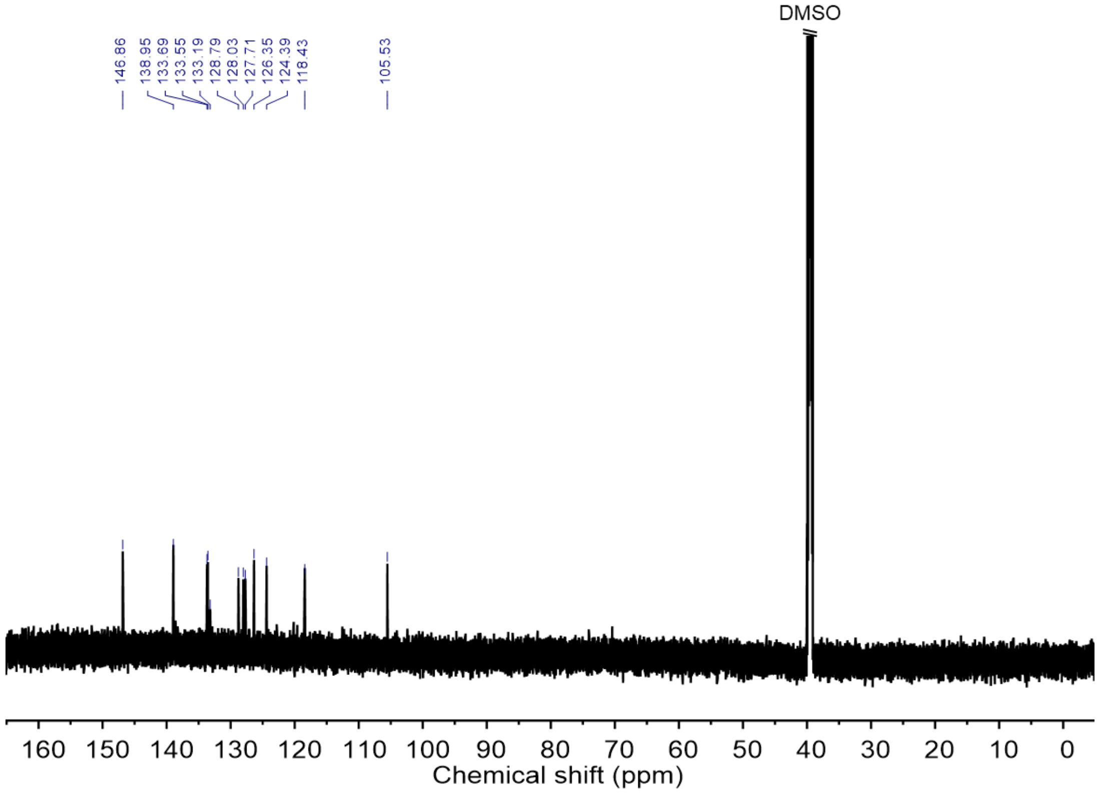

Supplementary Figure 2. ^{13}C NMR Spectrum (126 MHz, DMSO-d_6, 298K) of subcomponent **A.**

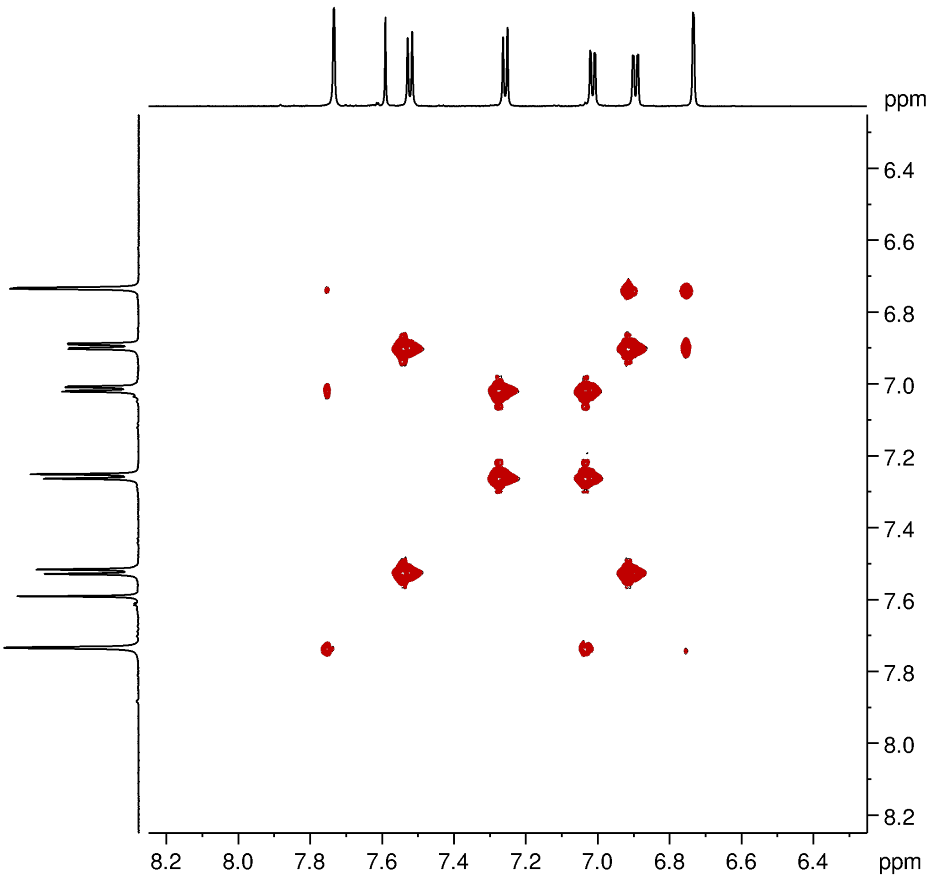

Supplementary Figure 3. Aromatic region of the ^{1}H-^{1}H DQF-COSY spectrum (500 MHz, DMSO-d_6, 298K) of subcomponent **A.**

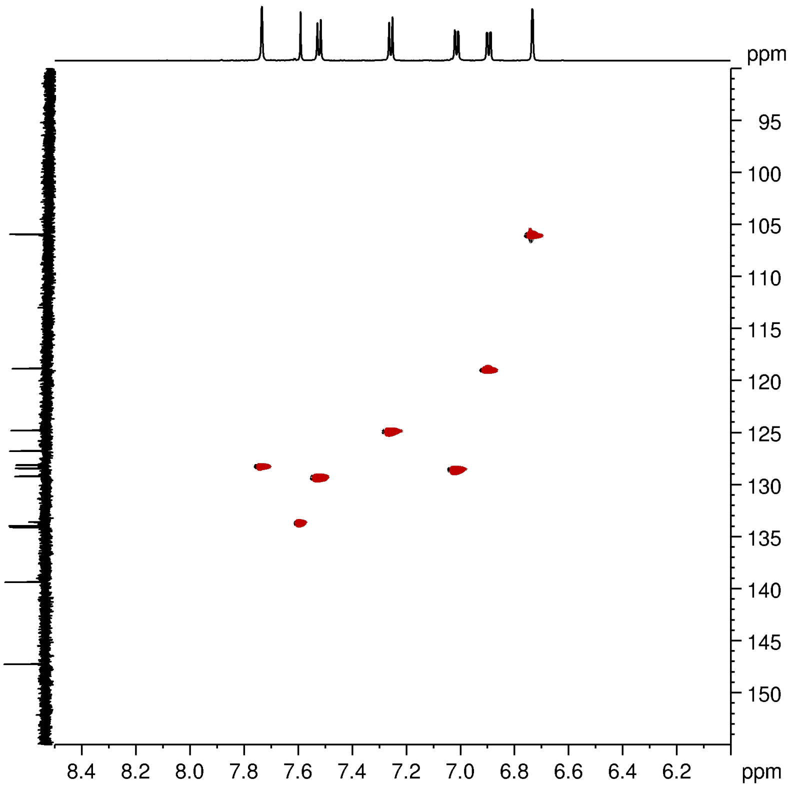

Supplementary Figure 4. Aromatic region of the ^{1}H-^{13}C HSQC spectrum (500 MHz, DMSO-d_6, 298K) of subcomponent **A.**

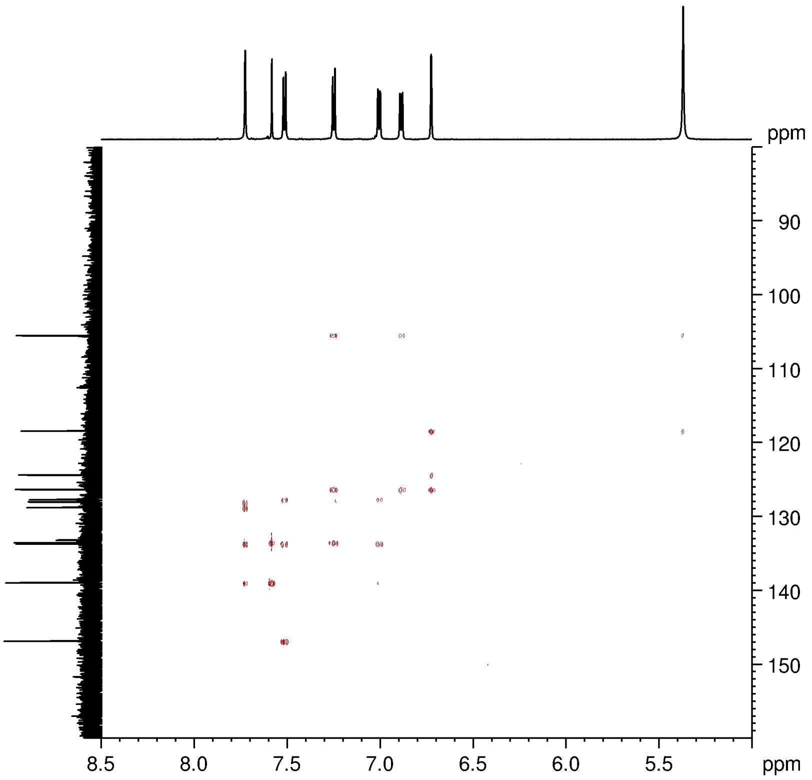

Supplementary Figure 5. Aromatic region of the ^{1}H-^{13}C HMBC spectrum (500 MHz, DMSO-d_6, 298K) of subcomponent **A.**

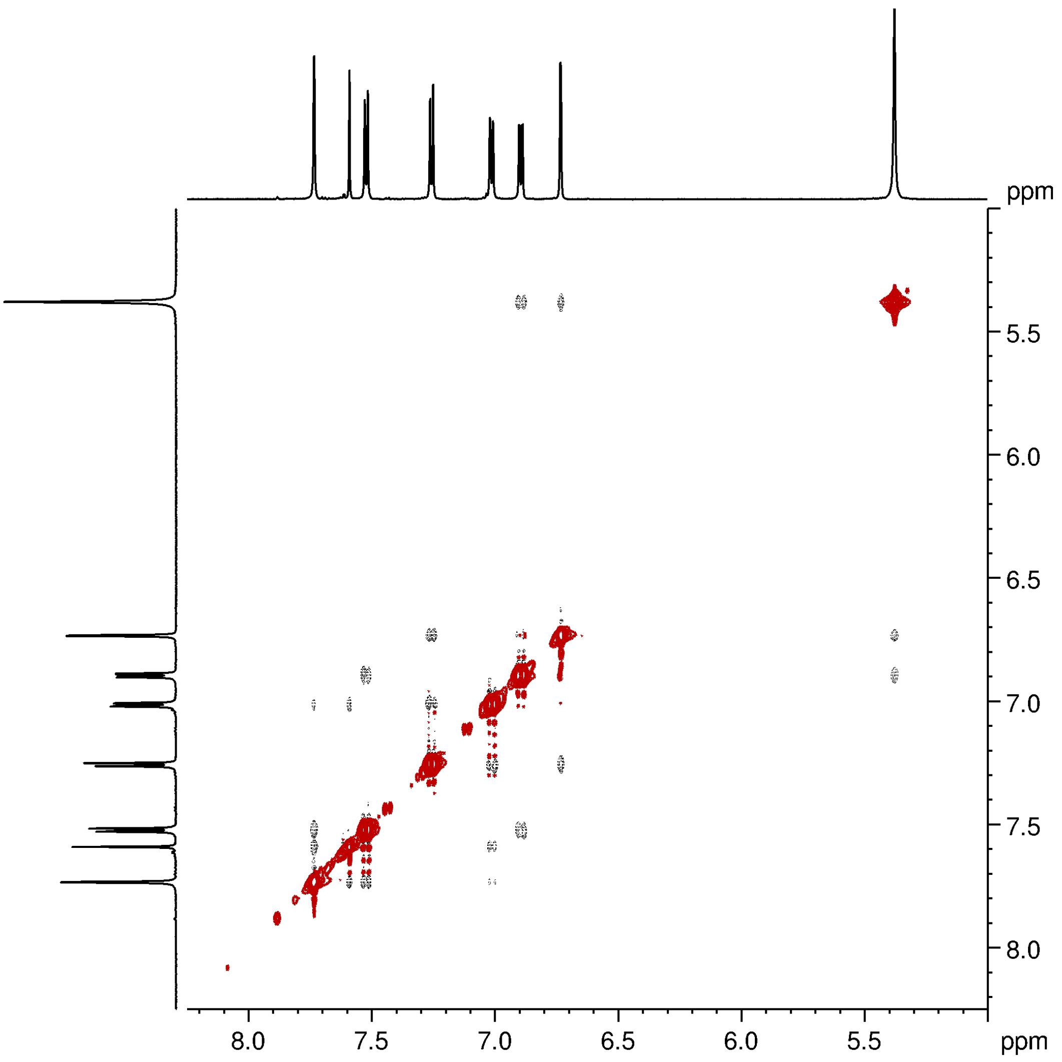

Supplementary Figure 6. Aromatic region of the ^1H-^1H ROESY spectrum (500 MHz, DMSO-d_6, 298K) of subcomponent **A**. The red (diagonal) and black signals (NOE cross peaks) are in two different phases respectively.

2.2 Pseudo cube 1

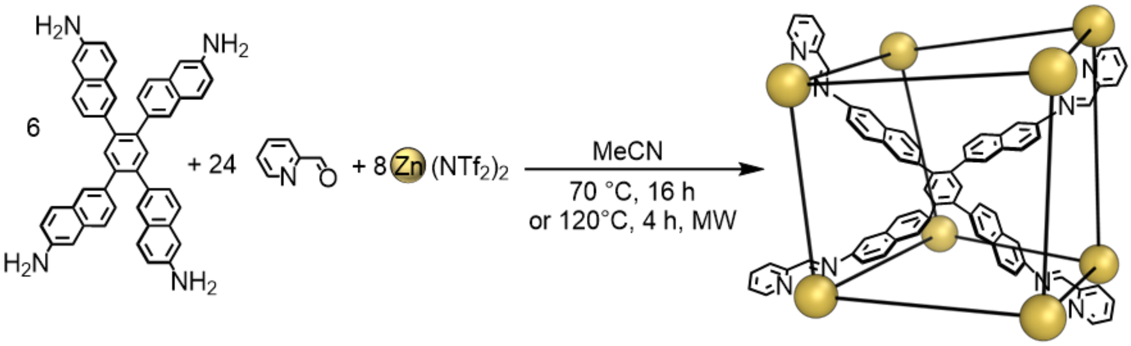

Supplementary Scheme 2. The self-assembly of pseudo cube **1**.

To a mixture of subcomponent **A** (20.0 mg, 31.1 µmol, 1.0 equiv) and $Zn(NTf_2)_2$ (26.0 mg, 41.5 µmol, 1.3 equiv) in distilled acetonitrile (8.0 mL), 2-formylpyridine (13.3 µL, 140 µmol, 4.5 equiv) was added. The reaction mixture was either heated at heated at 70 °C for 16 hours in a sealed vessel, or at 120 °C for 4 hours in a microwave reactor. After cooling to room temperature, the reaction mixture was filtered through a glass fibre plug, concentrated to a small volume under a stream of nitrogen, before the addition of diethyl ether (ca. 15 mL). The precipitate was separated with centrifugation and washed with diethyl ether (3 × 15 mL), affording **1**$\cdot(NTf_2)_{16}$ as a brownish yellow solid (50.7 mg, 4.6 µmol, 89%).

^{1}**H NMR** (700 MHz, CD_3CN, 298 K): δ = 8.64 (s, 6H), 8.60 (s, 6H), 8.56-8.52 (m, 12H), 8.50-8.45 (m, 12H), 8.38 (m, 6H), 8.33-8.27 (m, 18H), 8.24-8.11 (m, 42 H), 8.00-7.77 (m, 60H), 7.54 (s, 6H), ii7.51-7.43 (m, 12H), 7.43-7.32 (m, 18H), 7.29 (m, 6H), 6.56 (d, 6H, J = 8.6 Hz), 6.28-5.93 (m, 30 H), 5.34 (m, 6H), 5.23 (m, 6H), 5.04 (m, 6H), 4.69-4.34 (m, 18H).

^{13}**C NMR** (176 MHz, CD_3CN, 298 K): δ = 165.7, 165.3, 165.3, 164.2, 151.3, 151.0, 150.9, 150.3, 147.4, 147.3, 147.2, 147.1, 147.0, 146.9, 146.8, 145.9, 143.7, 143.6, 143.1, 141.2, 140.7, 140.4, 140.2, 139.7, 138.3, 135.3, 133.6, 133.0, 132.4, 132.4, 132.3, 132.2, 131.9, 131.7, 131.6, 131.2, 131.1, 130.9, 130.5, 130.3, 130.1, 129.9, 129.7, 129.4, 129.1, 128.9, 128.5, 127.4, 127.2, 123.5, 122.5, 122.4, 121.7, 121.3, 121.2, 120.7 (*J* = 321 Hz, NTf_2), 119.9, 119.1, 118.4, 118.3, 118.1, 118.1.

ESI-HRMS ([**1**$\cdot(NTf_2)_{16}$] = $C_{420}H_{276}N_{48}Zn_8(NS_2O_4C_2F_6)_{16}$) *m/z* = 636.4501 [**1**$\cdot(NTf_2)_4]^{12+}$ (calc. 636.5647) 719.7559 [**1**$\cdot(NTf_2)_5]^{11+}$ (calc. 719.9023) 819.8223 [**1**$\cdot(NTf_2)_6]^{10+}$ (calc. 819.9074) 942.1274 [**1**$\cdot(NTf_2)_7]^{9+}$ (calc. 942.1359) 1094.8838 [**1**$\cdot(NTf_2)_8]^{8+}$ (calc. 1094.9215) 1291.2837 [**1**$\cdot(NTf_2)_9]^{7+}$ (calc. 1291.3601) 1553.1493 [**1**$\cdot(NTf_2)_{10}]^{6+}$ (calc. 1553.2783) 1919.7624 [**1**$\cdot(NTf_2)_{11}]^{5+}$ (calc. 1919.9638)

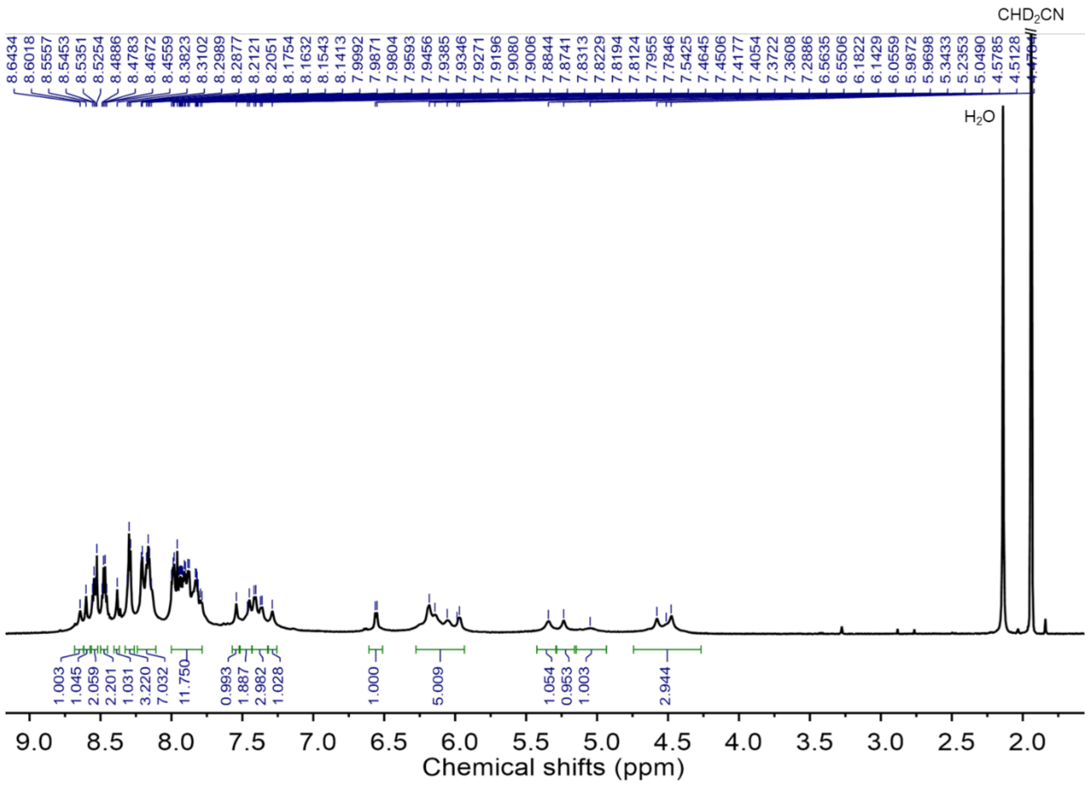

Supplementary Figure 7. ^1H NMR Spectrum (700 MHz, CD_3CN, 298K) of pseudo cube **1**·$(NTf_2)_{16}$.

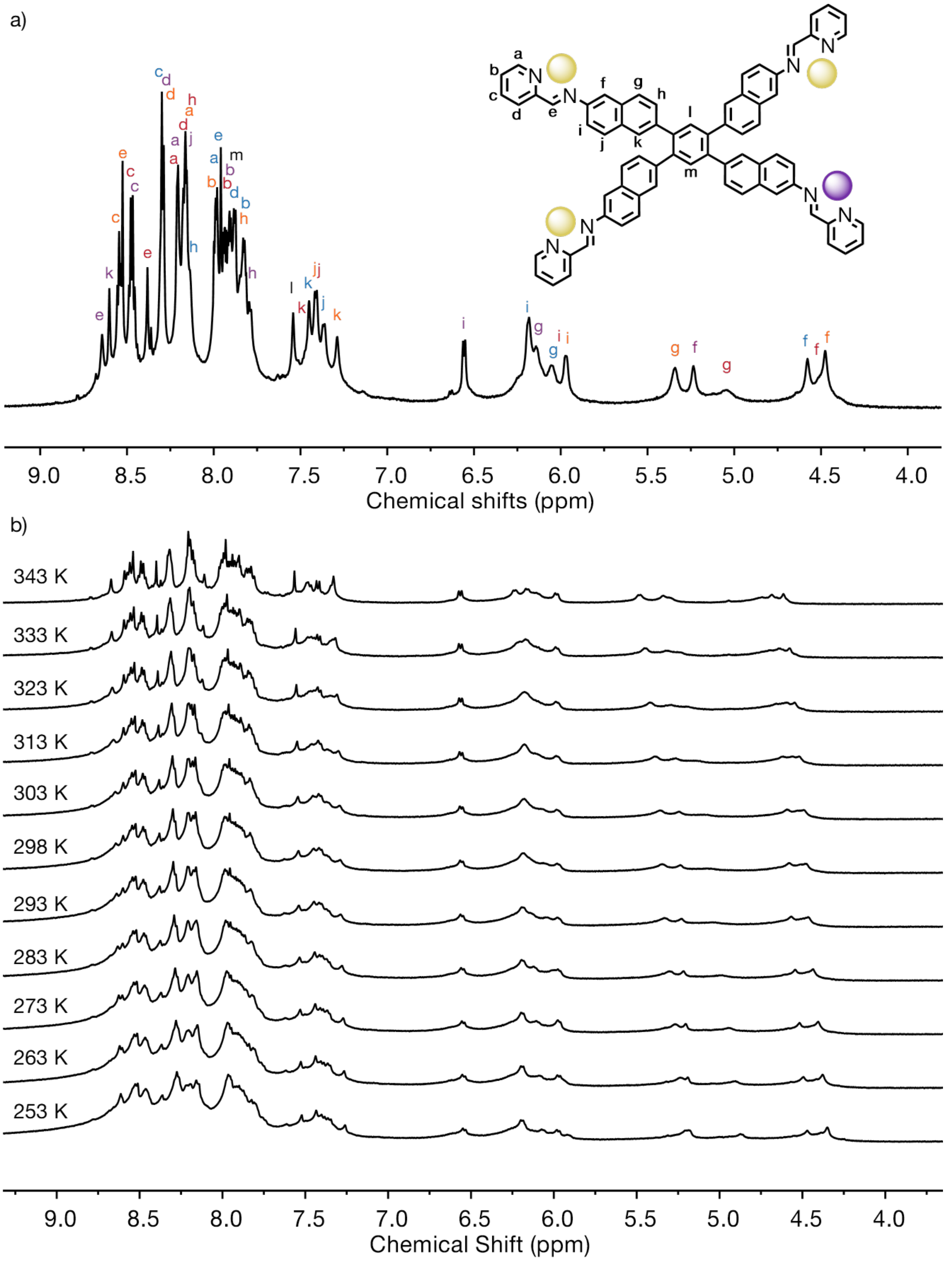

Supplementary Figure 8. a) Aromatic region of the ^{1}H NMR spectrum (700 MHz, CD_3CN, 298K) of pseudo cube **1**·$(NTf_2)_{16}$, with assignment of signals; b) ^{1}H VT NMR spectra (500 MHz, CD_3CN, 298K) of pseudo cube **1**·$(NTf_2)_{16}$, for a clearer view of the ^{1}H NMR signals of **1**·$(NTf_2)_{16}$. The signals associated with each distinct ligand arm could be distinguished and are marked with different colors; however, it was not possible to definitively attribute each set to a particular ligand arm within the structure due to the lack of a clear starting point for such assignments.

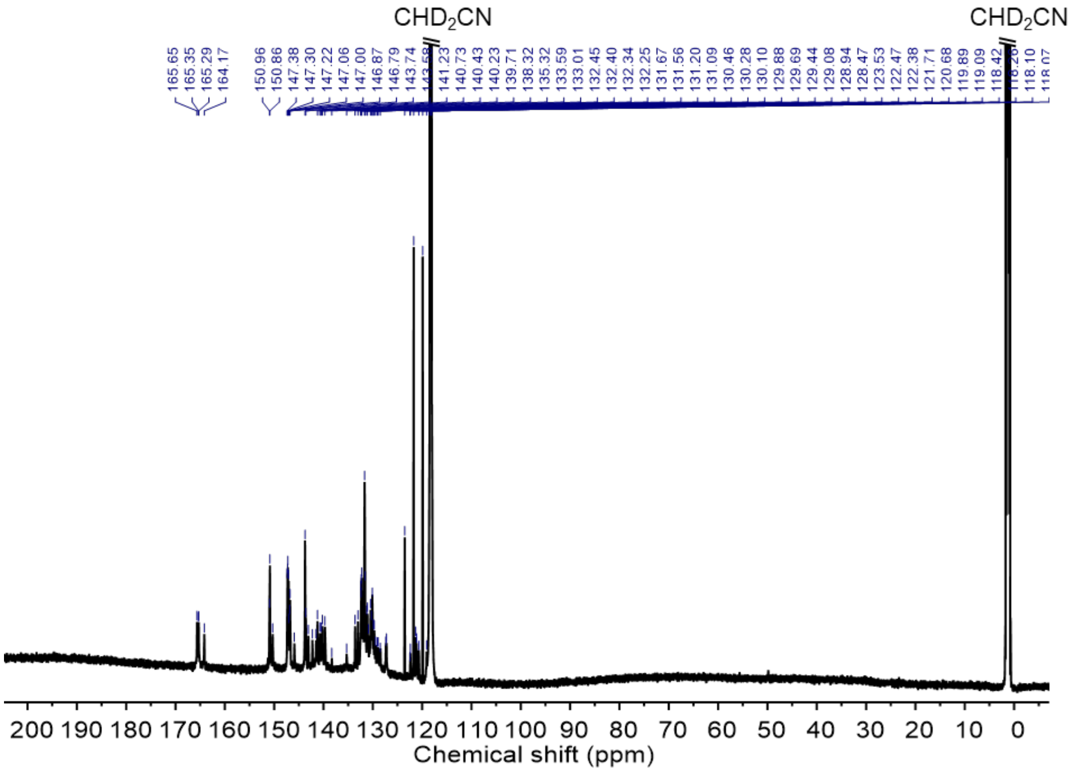

Supplementary Figure 9. ^{13}C NMR Spectrum (176 MHz, CD_3CN, 298K) of pseudo cube **1**·$(NTf_2)_{16}$.

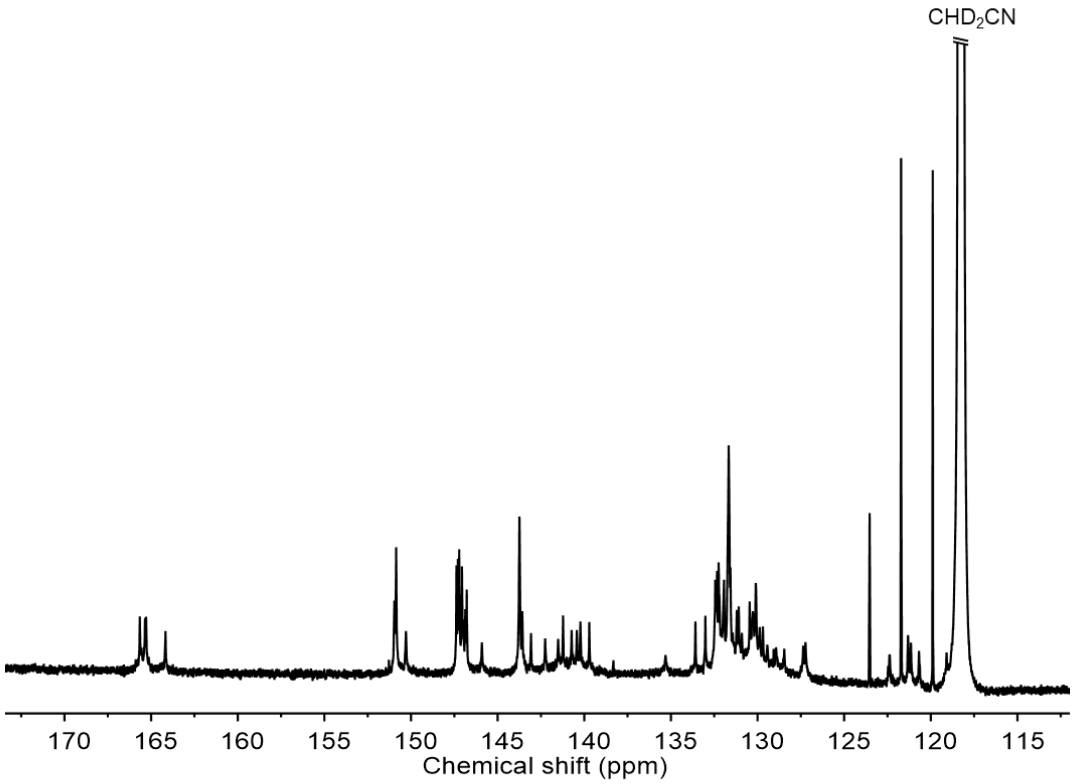

Supplementary Figure 10. Aromatic region of the ^{13}C NMR Spectrum (176 MHz, CD_3CN, 298K) of pseudo cube **1**·$(NTf_2)_{16}$.

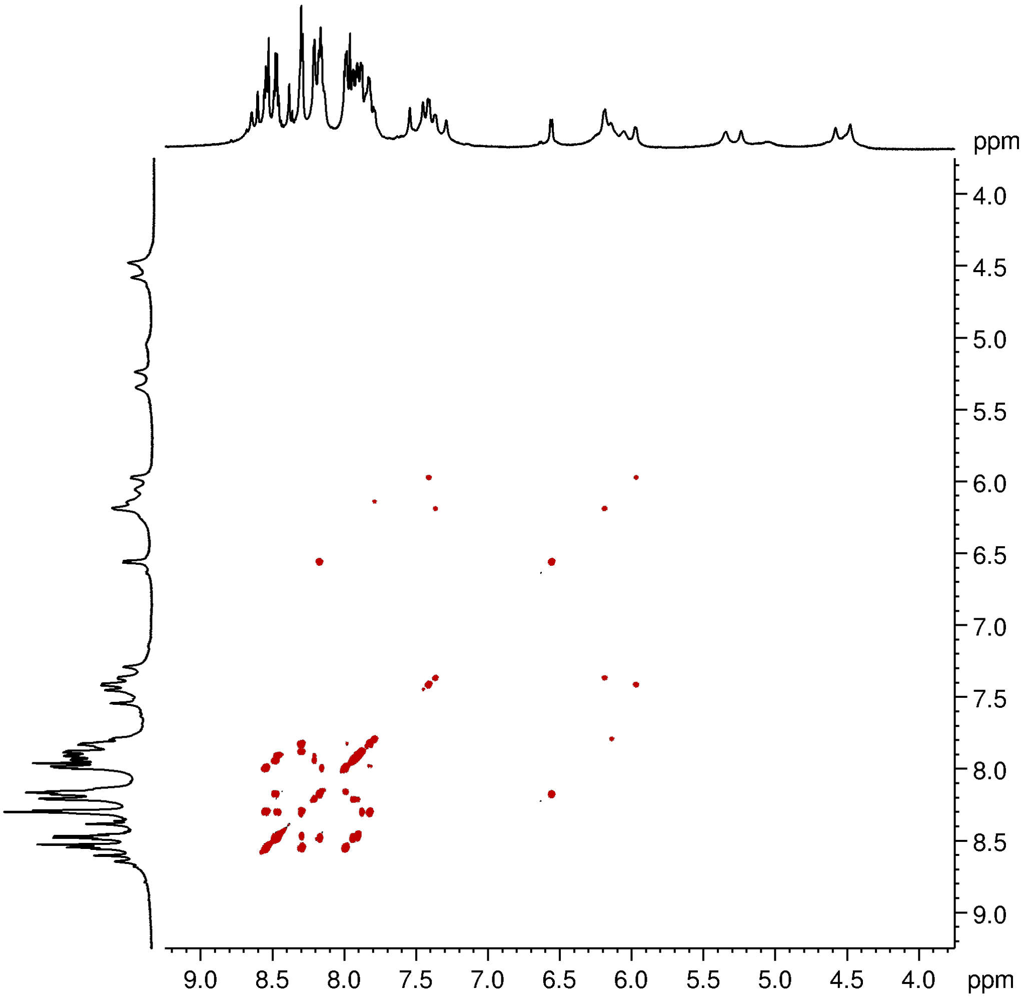

Supplementary Figure 11. Aromatic region of the ^{1}H-^{1}H DQF-COSY spectrum (700 MHz, CD_3CN, 298K) of pseudo cube **1**·$(NTf_2)_{16}$.

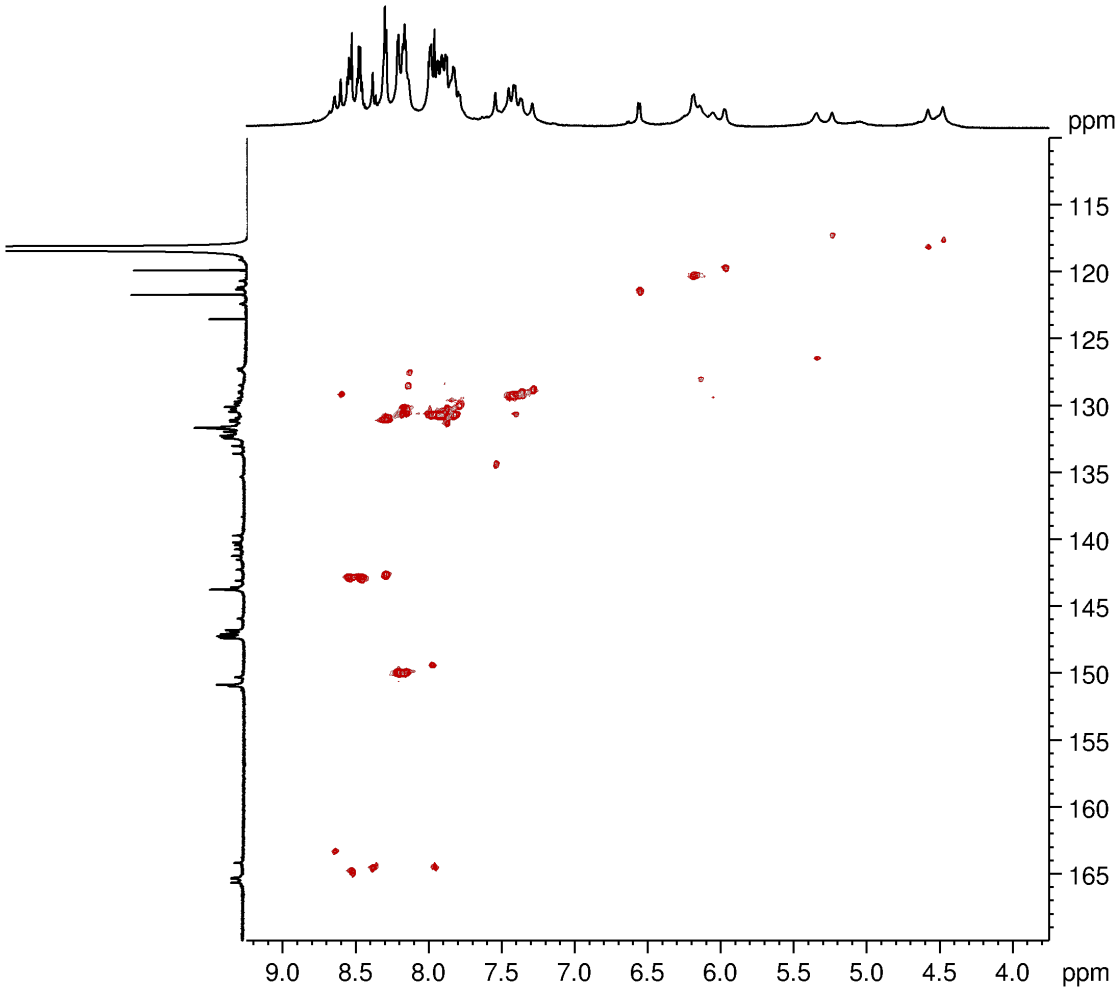

Supplementary Figure 12. Aromatic region of the ^{1}H-^{13}C HSQC spectrum (700 MHz, CD_3CN, 298K) of pseudo cube **1**·$(NTf_2)_{16}$.

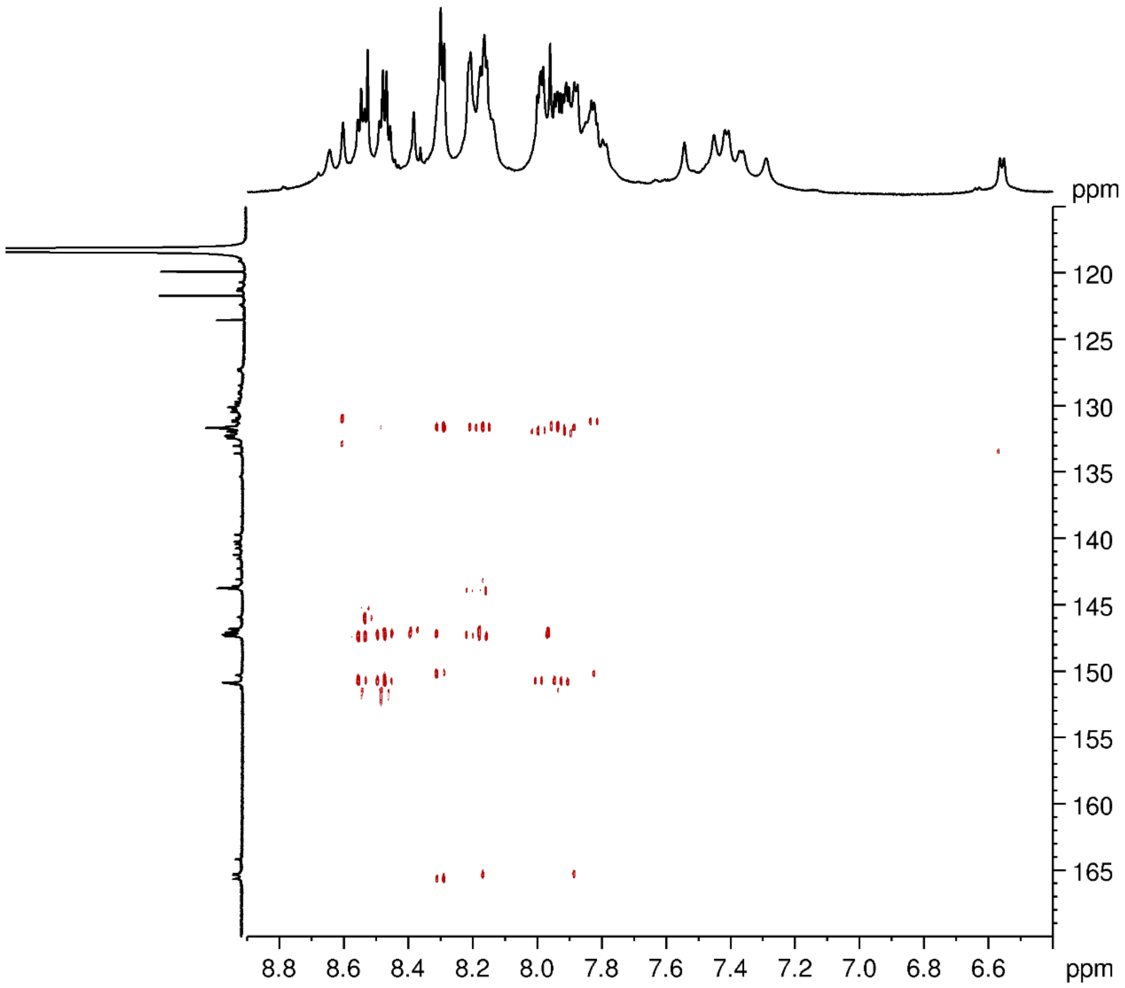

Supplementary Figure 13. Partial ^{1}H-^{13}C HMBC spectrum (700 MHz, CD_3CN, 298K) of pseudo cube **1**·$(NTf_2)_{16}$. No cross peaks of the cage have been found in other regions.

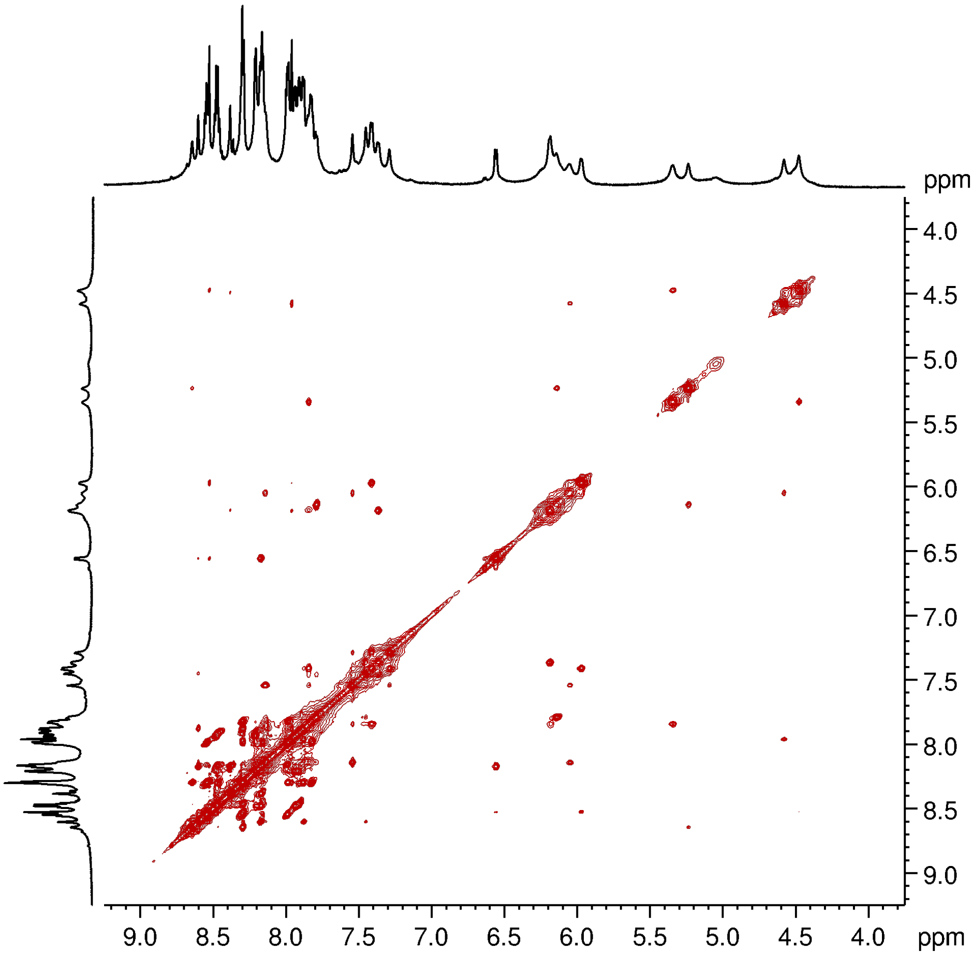

Supplementary Figure 14. Aromatic region of the ^{1}H-^{1}H NOESY spectrum (700 MHz, CD_3CN, 298K) of pseudo cube **1**·$(NTf_2)_{16}$.

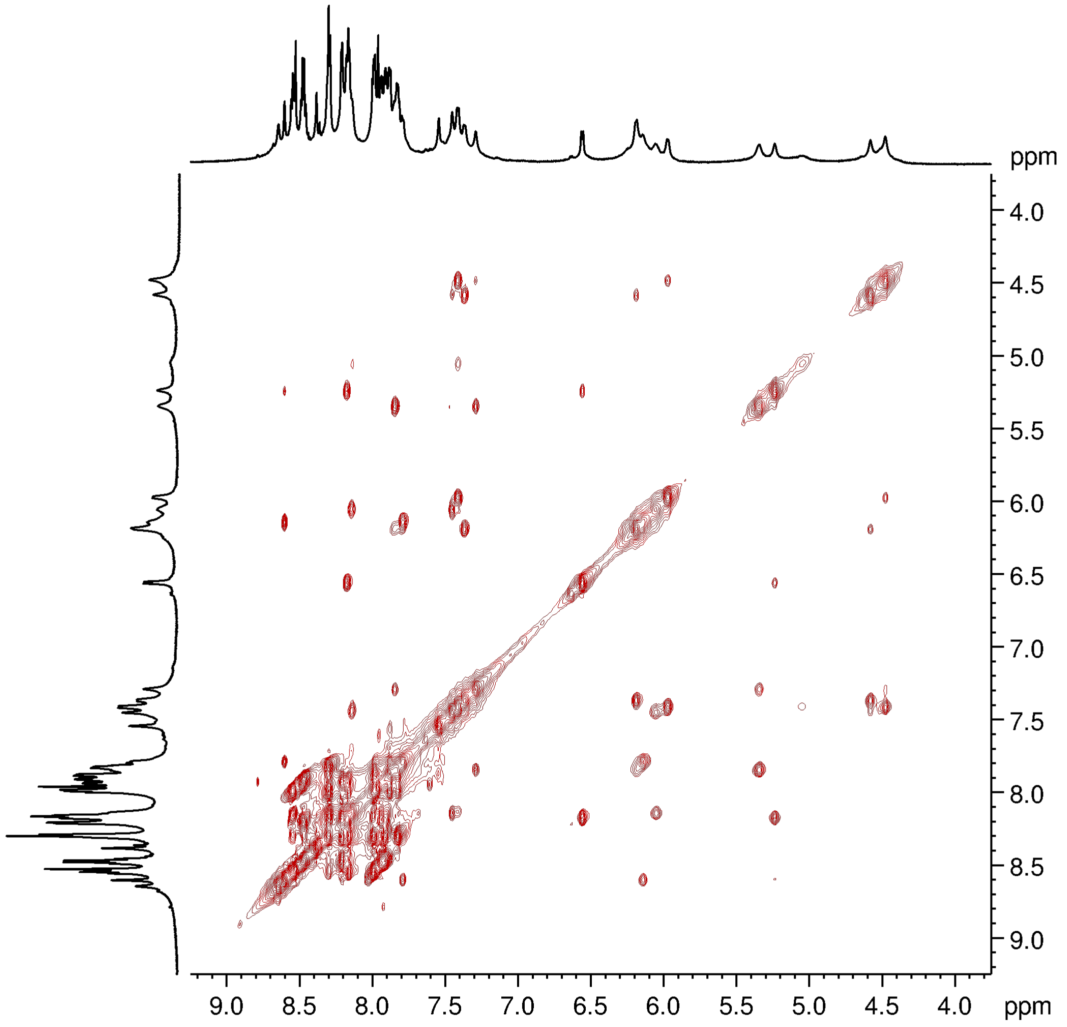

Supplementary Figure 15. Aromatic region of the ^{1}H-^{13}C TOCSY spectrum (700 MHz, CD_3CN, 298K) of pseudo cube **1**·$(NTf_2)_{16}$.

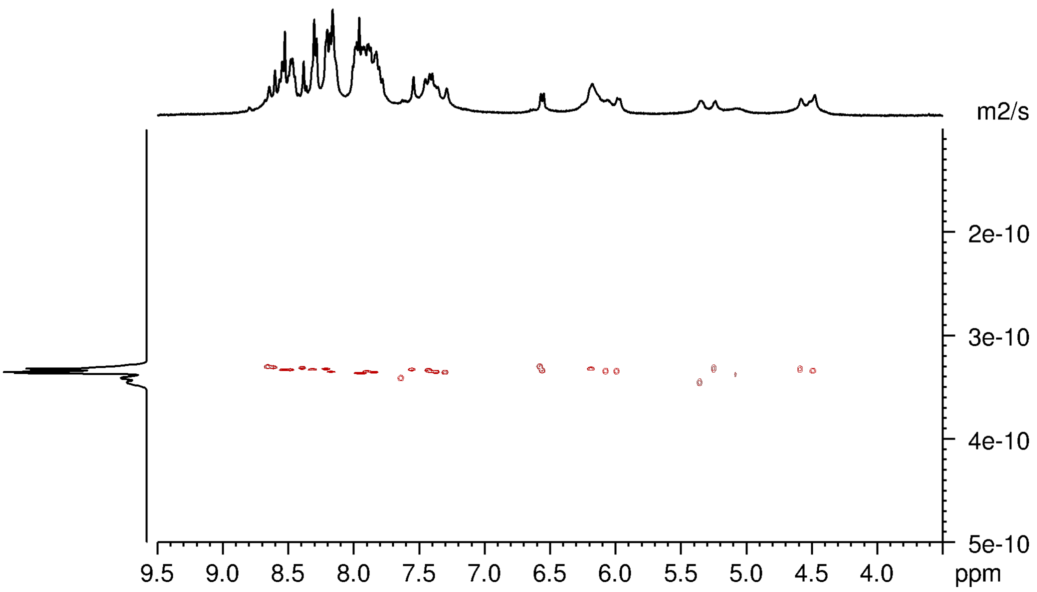

Supplementary Figure 16. ^{1}H-DOSY spectrum (400 MHz, CD_3CN, 298K) of pseudo cube **1**·$(NTf_2)_{16}$.

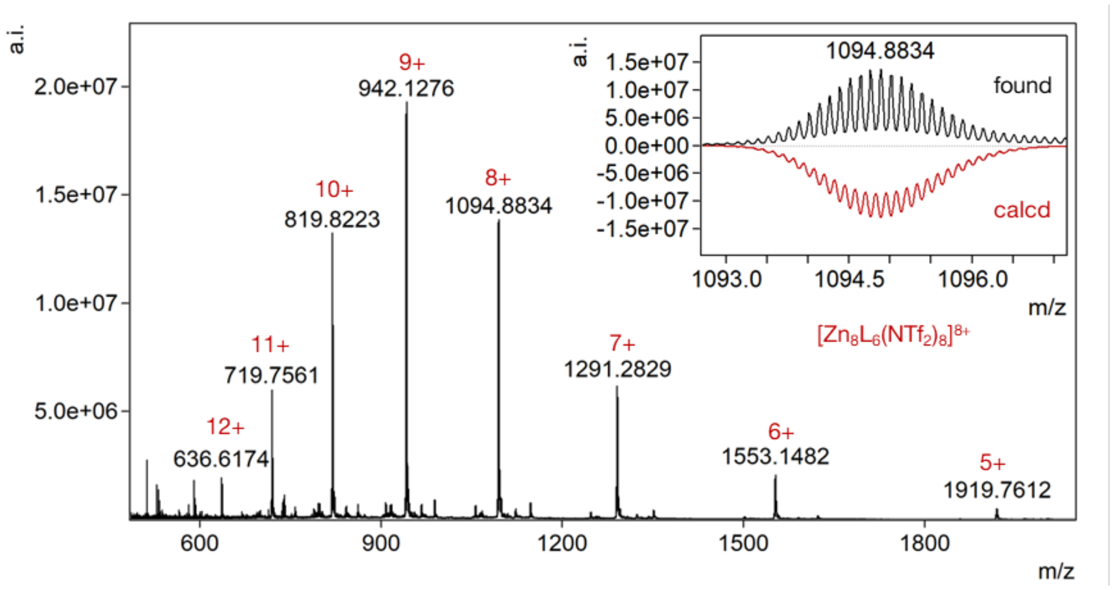

Supplementary Figure 17. ESI-HRMS spectrum of pseudo cube **1**, a Zn_8L_6·$(NTf_2)_{16}$ composition of the cage is evident.

3 X-ray crystallography

Data were collected at Beamline I19 of Diamond Light Source employing silicon double crystal monochromated synchrotron radiation (0.6889 Å) with ω and ψ scans at 100(2) K.[2] Data integration and reduction were undertaken with Xia2.[3-5] Subsequent computations were carried out using the Olex 2 1.2 graphical user interface.[6] Multi-scan empirical absorption corrections were applied to the data using the AIMLESS[7]

tool in the CCP4 suite.[8] The structures were solved by direct methods using SHELXT[9] then refined and extended with SHELXL.[10] In general, non-hydrogen atoms with occupancies greater than 0.5 were refined anisotropically. Carbon-bound hydrogen atoms were included in idealized positions and refined using a riding model. Disorder was modelled using standard crystallographic methods including constraints, restraints, and rigid bodies where necessary. Crystallographic data along with specific details pertaining to the refinement follow. Crystallographic data have been deposited with the CCDC (2367409). Crystal data, solution and refinement parameters are summarized in Supplementary Table 1.

Supplementary Table 1. Crystal Data, Solution and Refinement Parameters

	1
CCDC number	2367409
Formula	$C_{420}H_{276}F_{96}N_{48}Sb_{16}Zn_8$
Formula weight	10289.83
Temperature/K	100(2)
Crystal system	tetragonal
Space group	$I4^1/a$
a/Å	79.65280(10)
b/Å	79.65280(10)
c/Å	41.5024(2)
α/°	90
β/°	90
γ/°	90
Volume/Å^3	263314.8(14)
Z	16
ρ_{calc} g/cm^3	1.006
μ/mm-1	0.869
F (000)	78554.0

Crystal size/mm^3	0.20 × 0.20 × 0.10
Radiation	Synchrotron (λ = 0.6889)
2Θ range for data collection/°	1.402 to 40.296
Index ranges	-79 ⩽ h ⩽ 74, -74 ⩽ k ⩽ 79, -41 ⩽ l ⩽ 41
Reflections collected	457257
Independent reflections	68885 [R_{int} = 0.0652, R_{sigma} = 0.1042]
Goodness-of-fit on F^2	0.955
Final R indexes [I>=2σ (I)]	R_1[a]= 0.1187, wR_2[b]= 0.3251
Final R indexes [all data]	R_1 = 0.1763, wR_2 = 0.3480

[a] $R_1 = \Sigma||F_o| - |F_c|| /\Sigma |F_o|$; [b] $R_2 w = [\Sigma w(F_o^2 - F_c^2)^2 / w(F_o^2)^2]]^{1/2}$, where $w = q[\sigma^2(Fo^2) + (aP)^2 + bP]^{-1}$

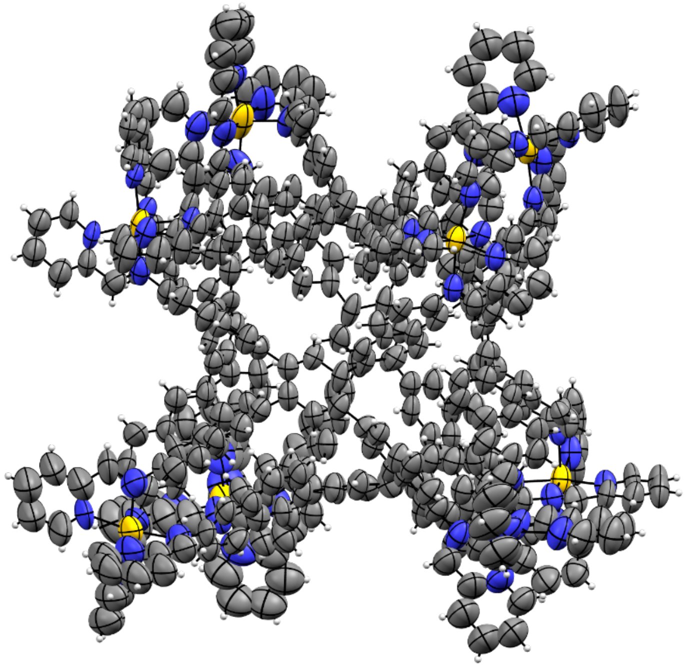

Supplementary Figure 18. Cationic portion of the crystal structure of **1**·13.2SbF_6, showing thermal ellipsoids at 50%. The asymmetric unit contains one whole structure (color codes: C = gray, N = light blue, Zn = yellow, H = white).

Specific refinement details:

The crystals of **1**·16SbF_6 were grown by diffusion of diethyl ether into an acetonitrile solution of **1**·16NTf_2 containing excess ($^{n}Bu_4N$)·SbF_6. The crystals employed immediately lost solvent after removal from the mother liquor and rapid handling prior to flash cooling in liquid nitrogen was required to collect data. Despite these measures and the use of synchrotron radiation few reflections at greater than 1.0 Å resolution were observed and the data were trimmed accordingly. Furthermore, there was a significant drop-off in diffraction intensity after around 1.4 Å resolution resulting in a low ratio of observed/unique reflections. Nevertheless, the quality of the data is easily sufficient to establish the connectivity of the structure. The asymmetric unit was found to contain one complete Zn_8L_6 assembly and associated counterions and solvent molecules.

In order to obtain a reasonable model for the organic parts of the structure DFIX restraints were applied to parts of the structure displaying a higher degree of thermal motion. Due to the thermal motion and less than ideal resolution, thermal parameter restraints (SIMU, RIGU) were applied to some pyridyl-imine moieties and counterions to facilitate anisotropic refinement. Several ligand sections were modelled as disordered over two locations with only the major occupancy parts refined anisotropically. The strongest remaining electron density peaks are close to the modelled counterions and may indicate minor unresolved disorder or may possibly

arise from absorption effects.

The anions within the structure show evidence of substantial disorder. Seven of the located SbF_6^- anions were modelled as disordered over two or three locations. The SbF_6^- anions were restrained to be approximately octahedral. Bond length restraints were applied to all the anions and most low occupancy anions were modelled with isotropic thermal parameters. The occupancies of all located anions were allowed to freely refine which resulted in a discrepancy of ca. 2.8 anions per Zn_8L_6 assembly.

Further reflecting the solvent loss and poor diffraction properties there is a significant amount of void volume in the lattice containing smeared electron density from disordered solvent and the remaining anions. Consequently the SQUEEZE[13] function of PLATON[14] was employed to remove the contribution of the electron density associated with these remaining anions and further highly disordered solvent, which gave a potential solvent accessible void of 94518 Å^3 per unit cell (a total of approximately 19501 electrons). Diffuse solvent molecules could not be assigned to acetonitrile or diethyl ether and were therefore not included in the formula. Consequently, the molecular weight and density given above are underestimated.

CheckCIF gives one A and two B level alerts. These alerts result from the limited resolution of the data and the poor diffraction properties.

4 Volume calculation

Molovol 1.0.0[15] has been used to calculate the cavity volume and shape of pseudo cube **1** from its crystal structure. The "Probe-occupied volume" (V_{occ}) have been obtained via calculation using the "single-probe mode". The parameters used for the calculation are:

Probe radius: 2.0 Å

Grid resolution: 0.1 Å

Optimization depth: 4

Element radii: Zn: 2.39 Å, N: 1.66 Å, H: 1.20 Å, C: 1.77 Å.

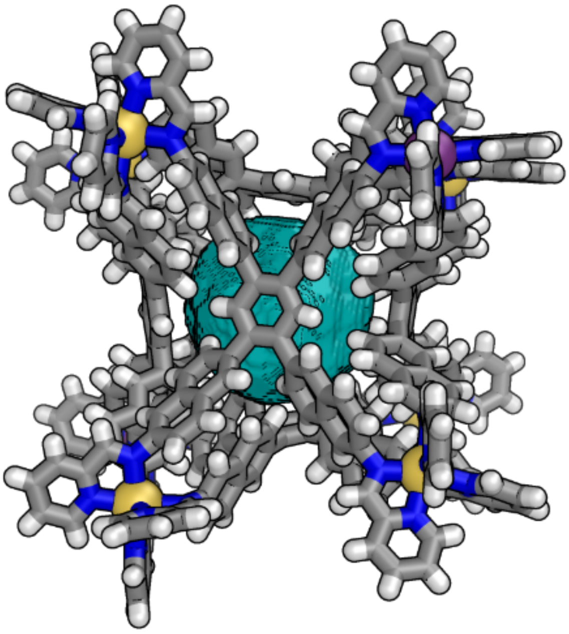

Supplementary Figure 19. MoloVol calculated void space (cyan mesh) within the crystal structure of 1. Color codes: C = gray, N = blue, *fac*-Δ-Zn = yellow orange, *fac*-Λ-Zn = purple.

Guest volumes were calculated with MoloVol 1.0.023 (Supplementary Table 2) based on molecular force field simulations (MM2 force field) obtained with Scrigress 3.4.5 (Fujitsu Limited) The following parameters were used in the volume calculations:

Probe mode: one probe

Probe radius: 1.2 Å

Grid resolution: 0.2 Å

Optimization depth: 4

Element radii: O: 1.5 Å, H: 1.2 Å, C: 1.77 Å, F: 1.46 Å, Cl: 1.82 Å, B: 1.91 Å.

Van der Waals volumes V_{vdW} were calculated and summarized in Supplementary Table 2. It is evident from Fig. S19 that the cavity shape of **1** is regular and approximately spherical. Yet, due to the open, convex structure of most guest molecules, these guest molecules take up more space than their volumes in the cavity of the cage due to their irregular shapes. To correct for that discrepancy between volume and size, we introduce the sphericity Ψ[16] (Equation S1):

$$\Psi = \frac{\pi^{\frac{1}{3}} \cdot (6\ V_{mol})^{\frac{2}{3}}}{S_{excl}} \qquad \text{(S1)}$$

with V_{mol} being the calculated molecular volume of the guest, and S_{excl} the probe excluded molecular surface (Supplementary Table 2). The higher the sphericity of a

non-tetrahedral guest at a given volume, the better the match to a cubic or nearly spherical cavity. The sphericity corrected molecular volumes Vmol·Ψ^{-1} have been summarized in Supplementary Table 2.

Supplementary Table 2. Calculated and sphericity corrected guest volumes

	V_{vdW} (Å^3)	S_{excl} (Å^2)	V_{mol} (Å^3)	Ψ	Sphericity corrected volume (Å^3)
Adamantane	150	159	160	0.90	178
α-Pinene	160	174	170	0.85	200
trans-Decalin	162	176	173	0.85	203
Diamantane	197	192	210	0.89	236
trans-Perfluorodecalin	243	230	262	0.86	304
Corannulene	231	233	236	0.79	298
Perhydropyrene	243	235	261	0.84	311
Aldrin	260	248	278	0.83	334
Endrin	265	247	284	0.85	336
γ-Chlordane	273	260	296	0.83	358
Kepone hydrate	308	271	336	0.86	389
Dienochlor	299	289	318	0.78	409
Mirex	327	281	356	0.86	413
Tetraphenylborate	332	327	347	0.73	475
Tetrakis (4-fluorophenyl)borate	349	345	369	0.72	512
Tetrakis (4-chlorophenyl)borate	394	389	415	0.69	599

5 Guest binding studies

5.1 Neutral guests

Depending on the distinct amounts of guest required to result in clear NMR and mass spectra of the host-guest complex, the host-guest complexes in this study were prepared by directly mixing 5.50 mg (0.5 μmol) of cage **1** and different equivalents of the guest (specified below) in 0.5 mL of CH_3CN or CD_3CN and heating at 70 °C until there is no further change on the spectra, which indicates that equilibrium is reached. To get clearer results, where applicable, the equilibrated mixtures with some guests were purified by precipitation via the addition of an excess amount (15 mL) of diethyl ether to remove excess guest, dried under *vacuo*, and redissolved in 0.5 mL of CH_3CN or CD_3CN for characterization.

5.1.1 ^{1}H NMR spectra

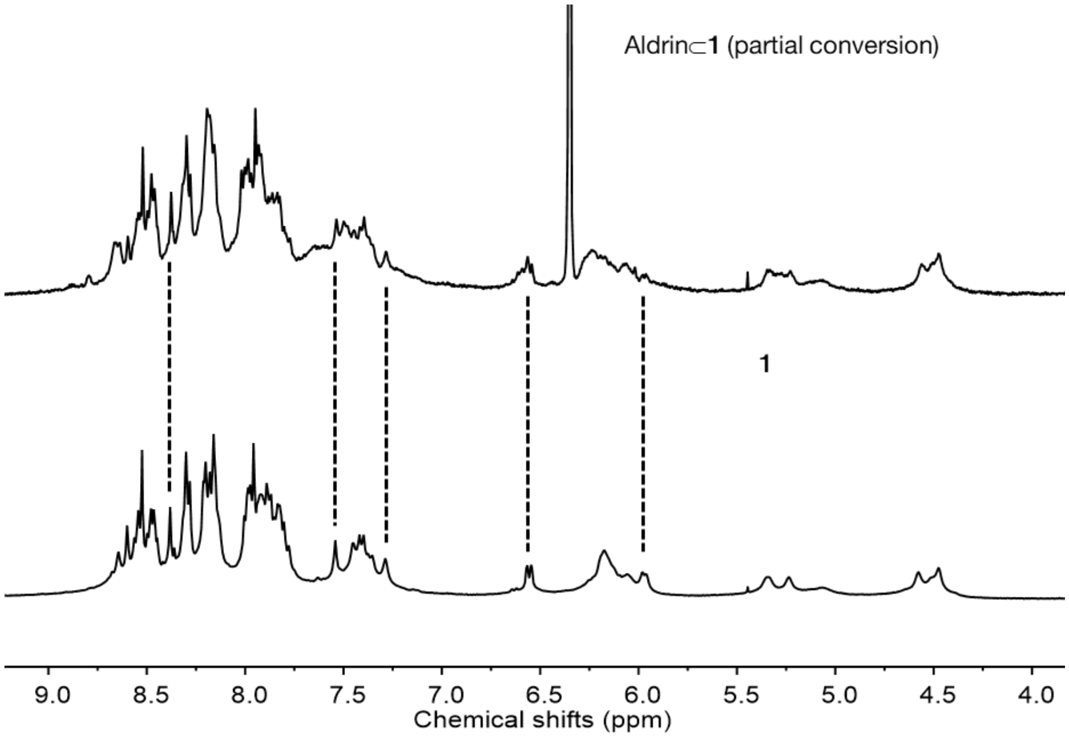

Supplementary Figure 20. An example of host conversion happening upon guest binding to **1**. 10 equivalents of aldrin and were added to a 1 mM solution of **1** and the ^{1}H NMR spectrum was recorded immediately, compared with the ^{1}H NMR spectrum of empty **1** (CD_3CN, 298 K, 400 MHz). A new set of host signals appeared and co-existed with the original host peaks (highlighted by black dashed lines), indicating a conversion process of the host taking place, yet the conversion was not complete unless the sample is heated at 70 °C for 16 h (Supplementary Figure 29). It is evident from the guest signals in Supplementary Figure 28b that the chemical exchange is faster than the NMR time scale, but two sets of host signals are still visible here.

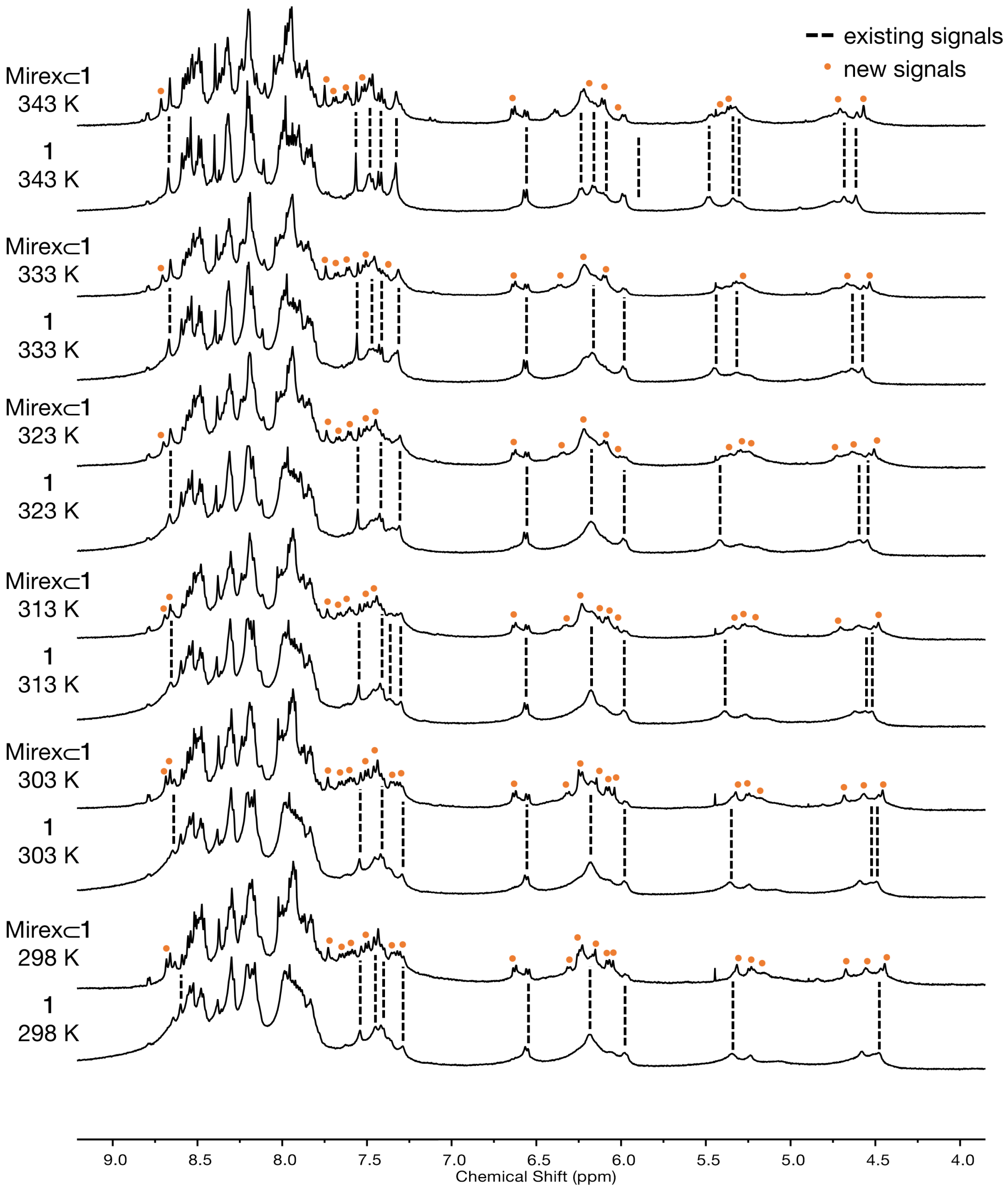

Supplementary Figure 21. Comparison of VT ^{1}H NMR spectra of mirex⊂**1** (prepared by adding 10 equivalents of mirex to a 1 mM solution of **1** and heating to 343 K for 16 h) and empty **1** (CD_3CN, 298 K, 500 MHz). A new set of host signals appeared and co-existed with the original host peaks. A conversion process of the host similar to that in Supplementary Fig. S20 is evident. A set of new host signals has appeared. Representative unchanged host signals have been highlighted with black dashed lines, while the new signals are highlighted with orange dots.

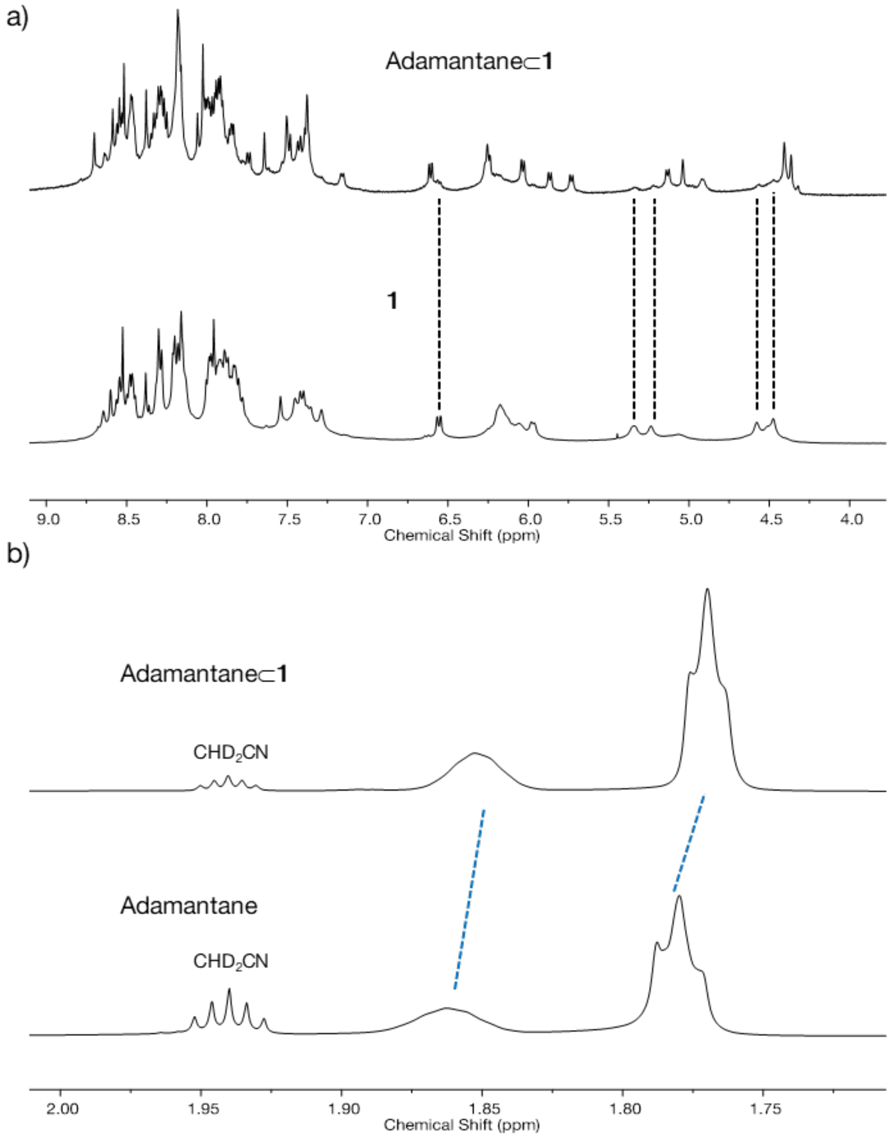

Supplementary Figure 22. ^{1}H NMR spectrum of adamantane⊂**1** (with ca. 10 equiv. of adamantane), with comparison to the ^{1}H NMR of a) empty cage **1** and b) the free guest (CD_3CN, 298 K, 400 MHz for the spectra of free adamantane and **1** and 500 MHz for that of adamantane⊂**1**). Note that a new set of host signals have emerged in addition to the signals of empty **1**. All the guest signals have shifted. Representative remaining empty host peaks and new host peaks are highlighted by black dashed lines and blue dashed lines, respectively.

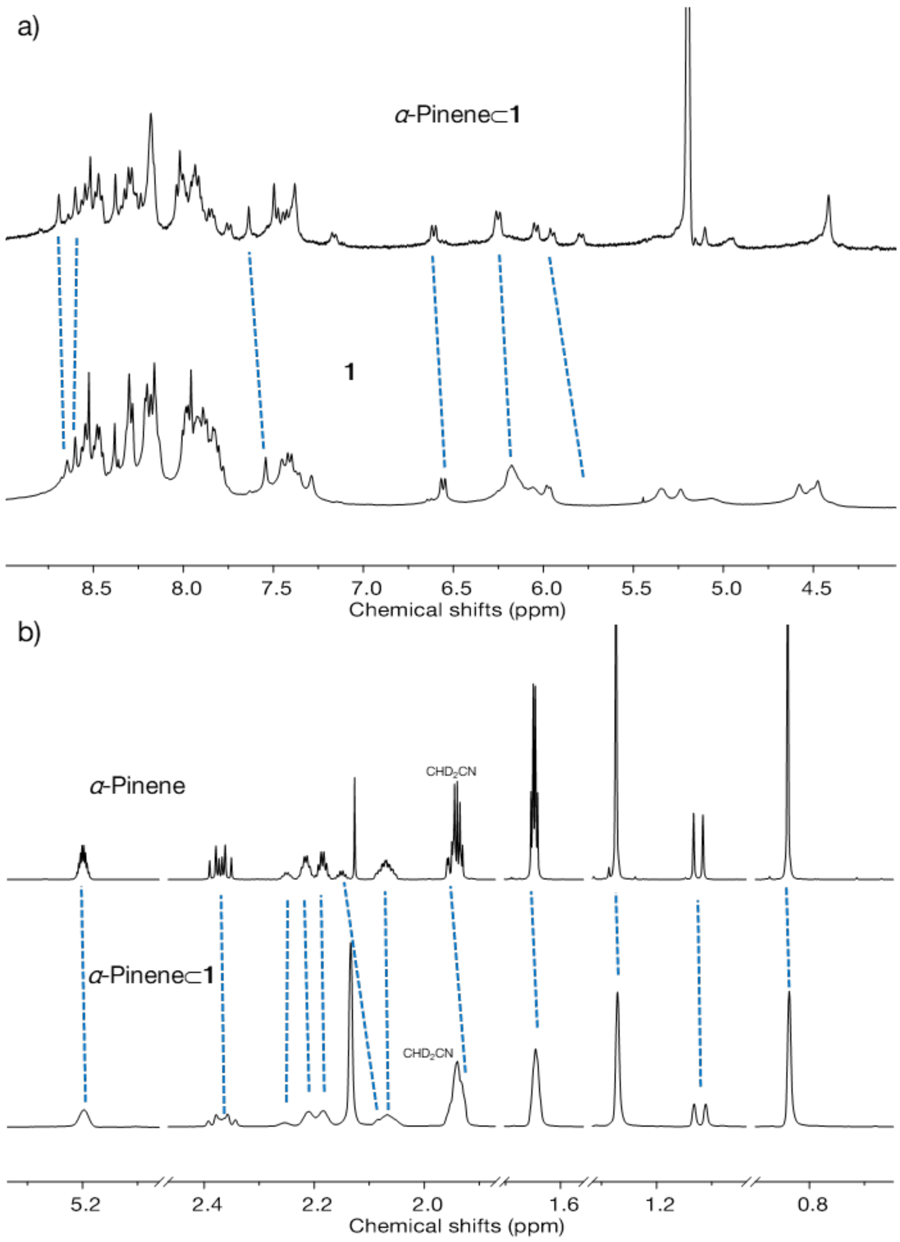

Supplementary Figure 23. ^{1}H NMR spectrum of *α*-pinene⊂**1** (with ca. 15 equiv. of *α*-pinene), with comparison to the ^{1}H NMR of a) empty cage **1** and b) the free guest (CD_3CN, 298 K, 400 MHz for the spectra of **1** and *α*-pinene⊂**1** and 500 MHz for free *α*-pinene). Note that a new set of host signals have emerged in addition to the signals of empty **1**. The shifts of guest signals are limited due to the large amount of guest used to convert **1** to *α*-pinene ⊂**1** for the sake of spectral clarity. Representative new host signals and shifted guest signals are highlighted by blue dashed lines.

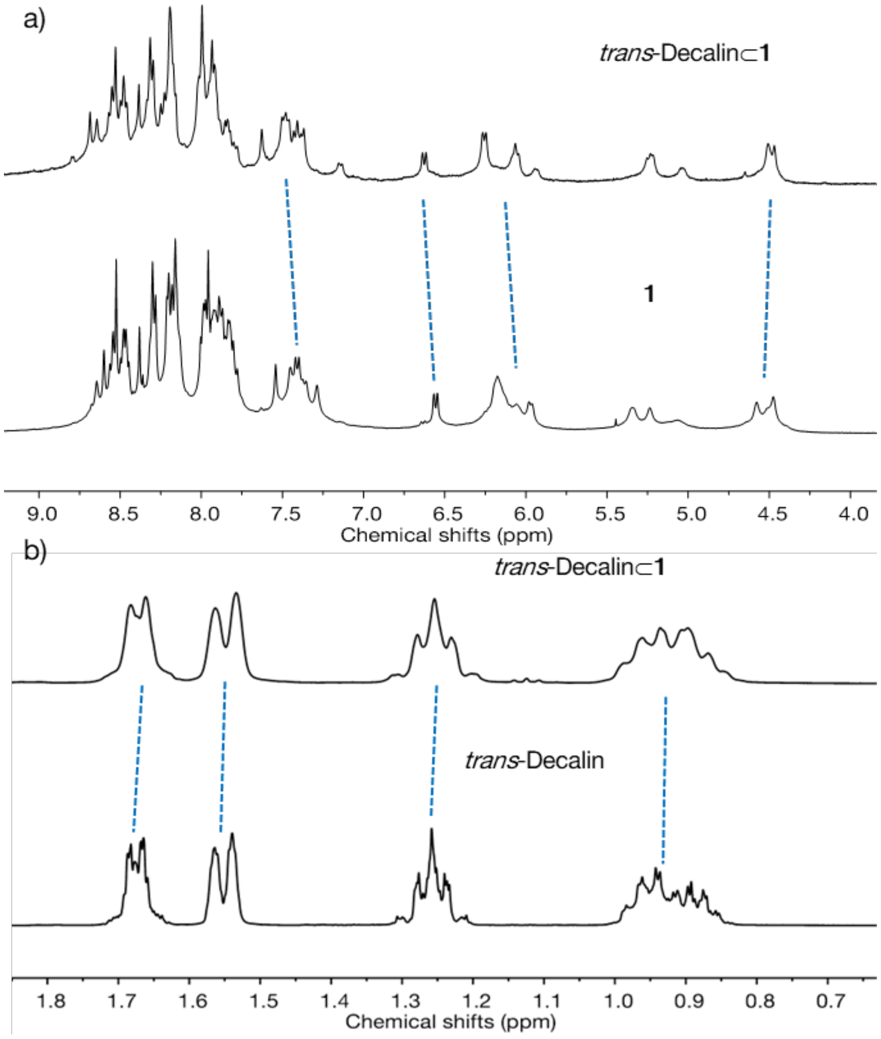

Supplementary Figure 24. ^{1}H NMR spectrum of *trans*-decalin⊂**1** (with ca. 15 equiv. of *trans*-decalin), with comparison to a) the empty cage **1** and b) the free guest (CD_3CN, 298 K). All the signals of **1** and the guest have shifted. The shifts of guest signals are limited partly due to the large amount of guest used to convert **1** to *trans*-decalin⊂**1** for the sake of spectral clarity. Representative shifted host and guest signals are highlighted by blue dashed lines.

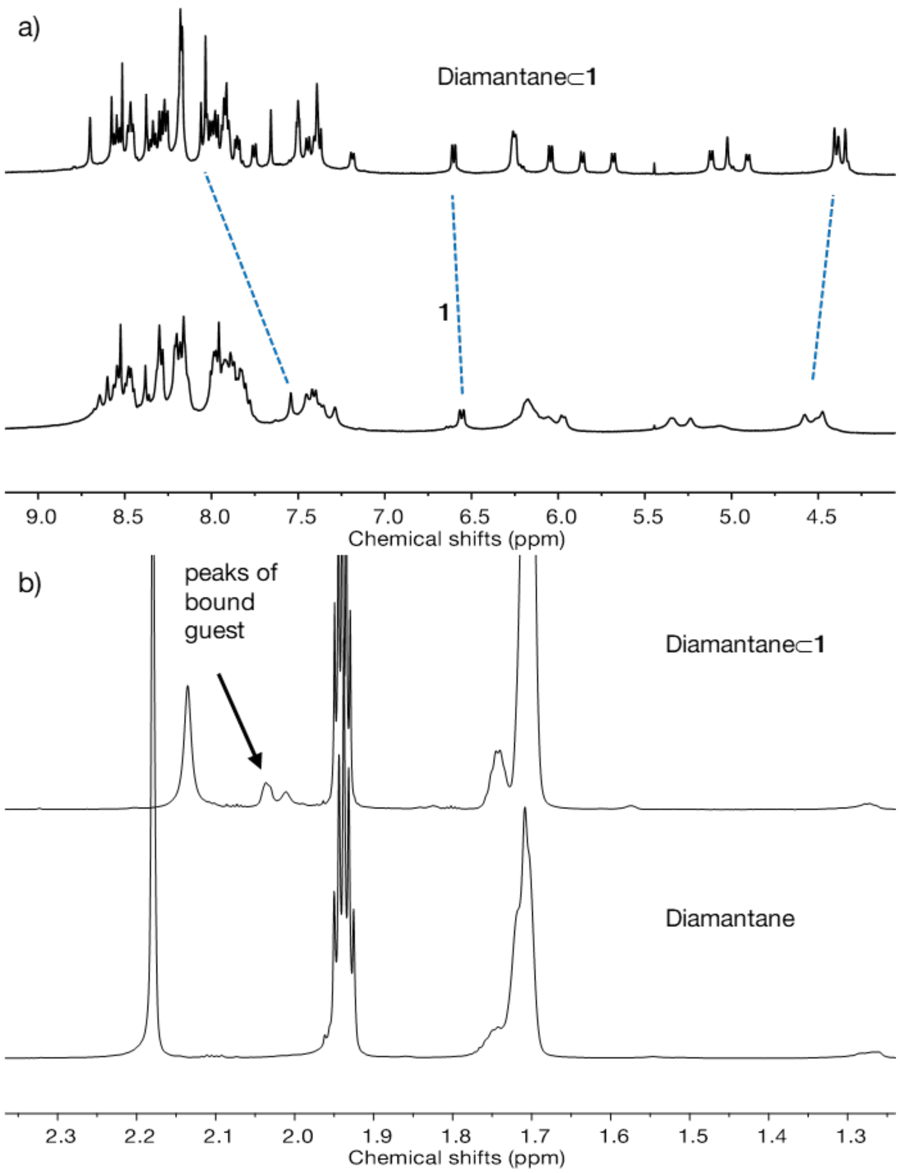

Supplementary Figure 25. ^{1}H NMR spectrum of diamantane⊂**1** (with ca. 10 equiv. of diamantane), with comparison to the ^{1}H NMR of a) empty cage **1** and b) the free guest (CD_3CN, 298 K, 400 MHz for the spectra of free diamantane and **1** and 500 MHz for that of diamantane⊂**1**). Note that a new set of host signals have emerged in addition to the signals of empty **1** and a new set for the guest, consistent with slow exchange binding on the NMR timescale. Representative new signals are highlighted by blue dashed lines.

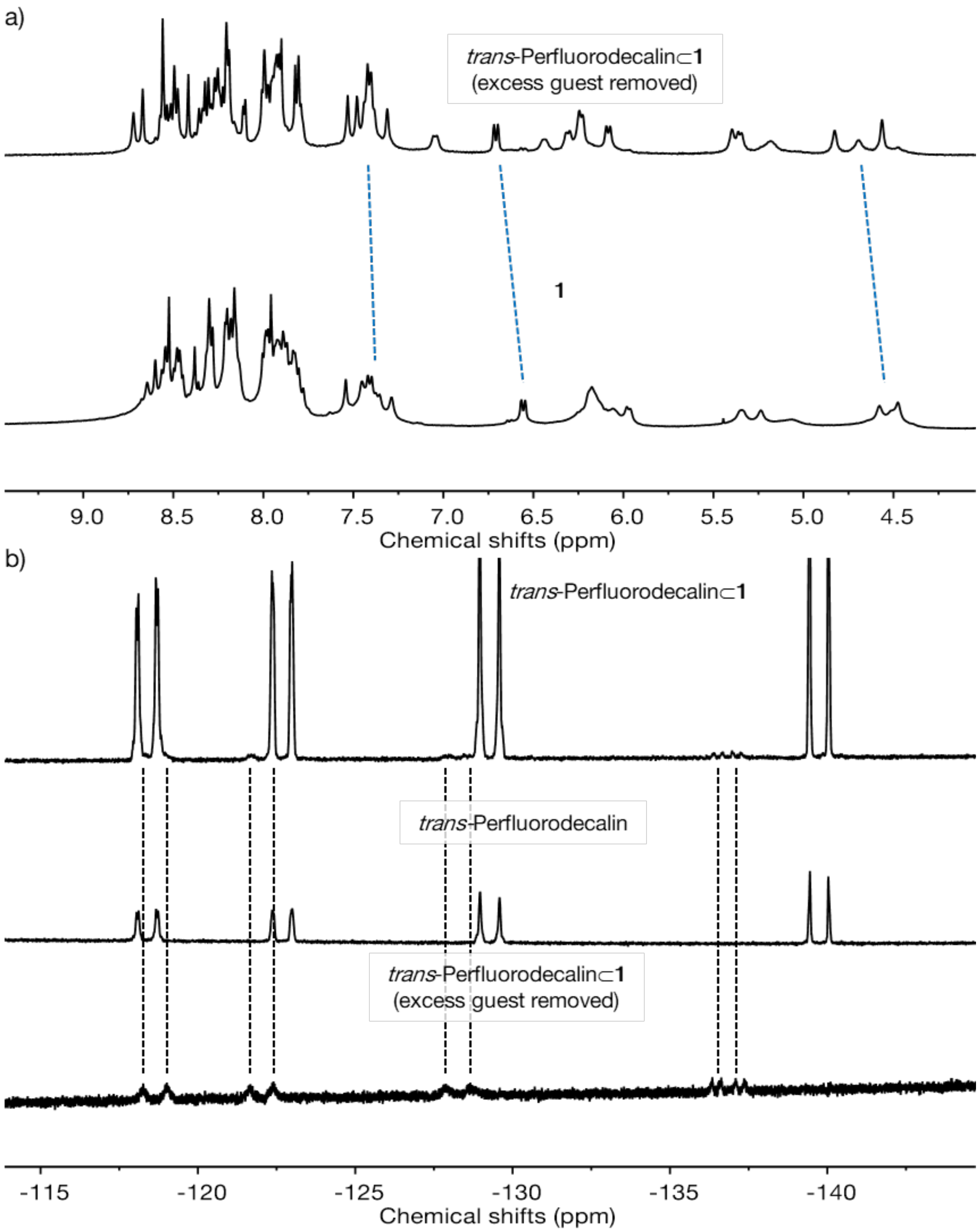

Supplementary Figure 26. a) ^{1}H NMR spectrum of *trans*-perfluorodecalin⊂**1** (prepared with ca. 20 equiv. of *trans*-perfluorodecalin, precipitated by addition of Et_2O and redissolved), in comparison with empty cage **1** and b) ^{19}F NMR spectrum of *trans*-perfluorodecalin⊂**1** with excess guest and with excess guest removed by precipitation with Et_2O and redissolving in CD_3CN, in comparison with the free guest. (CD_3CN, 298

K, 400 MHz for the spectrum of **1**, 500 MHz for *trans*-perfluorodecalin⊂**1**, and 470 MHz for all ^{19}F NMR spectra). Note that a new set of host signals and the guest have emerged. Bound guest peaks and representative new host peaks are highlighted by black dashed lines and blue dashed lines, respectively.

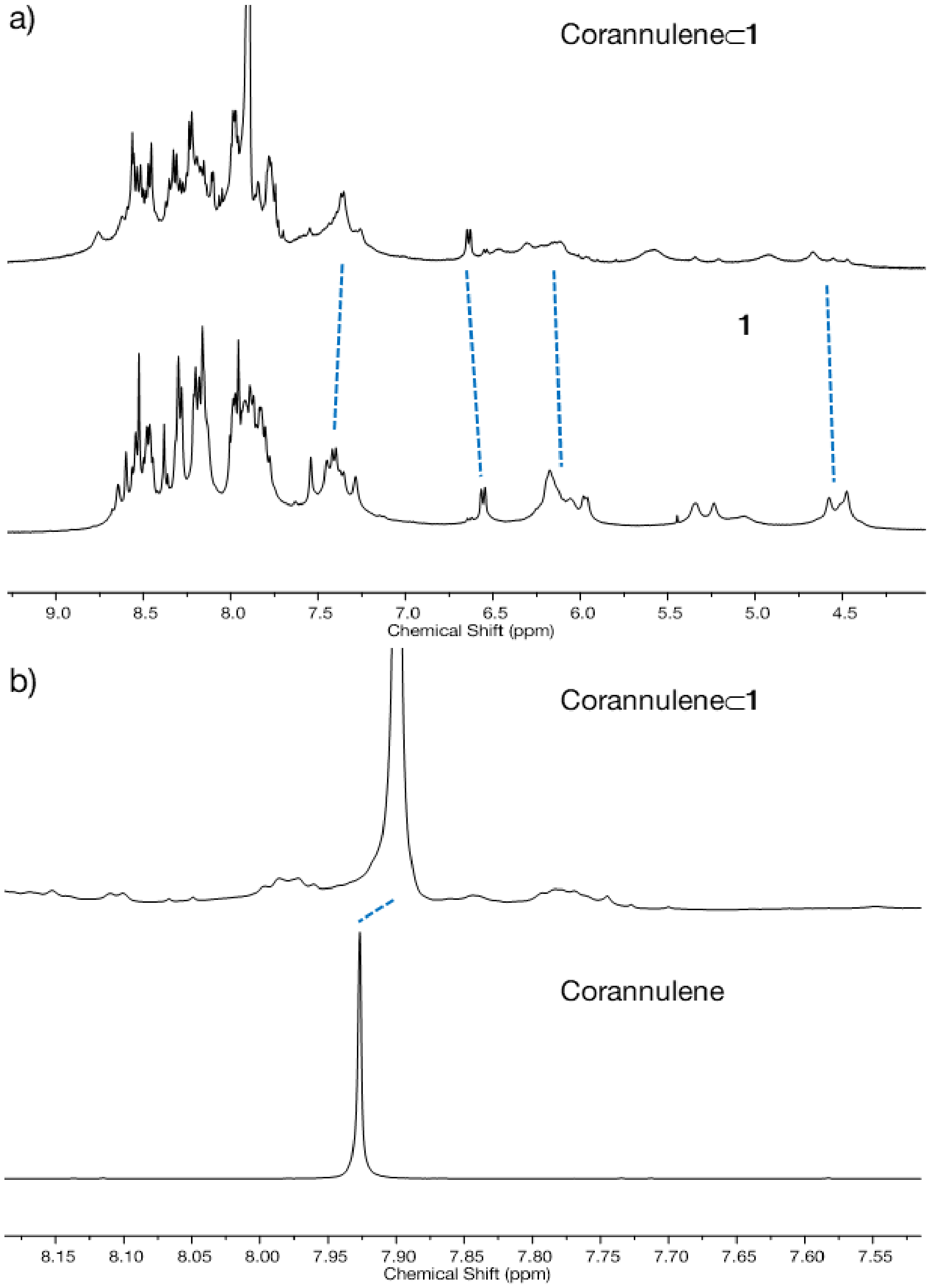

Supplementary Figure 27. ^{1}H NMR spectrum of corannulene⊂**1** (prepared with ca. 20 equiv. of corannulene), with comparison to the ^{1}H NMR of a) empty cage **1** and b) the free guest (CD_3CN, 298 K, 400 MHz for the spectra of free corannulene and **1** and 500 MHz for that of corannulene⊂**1**). Note that at least one set of new host signals have emerged in addition to the signals of empty **1**. The signal of the guest has shifted. Representative new host signals and shifted guest signals are highlighted by blue

dashed lines.

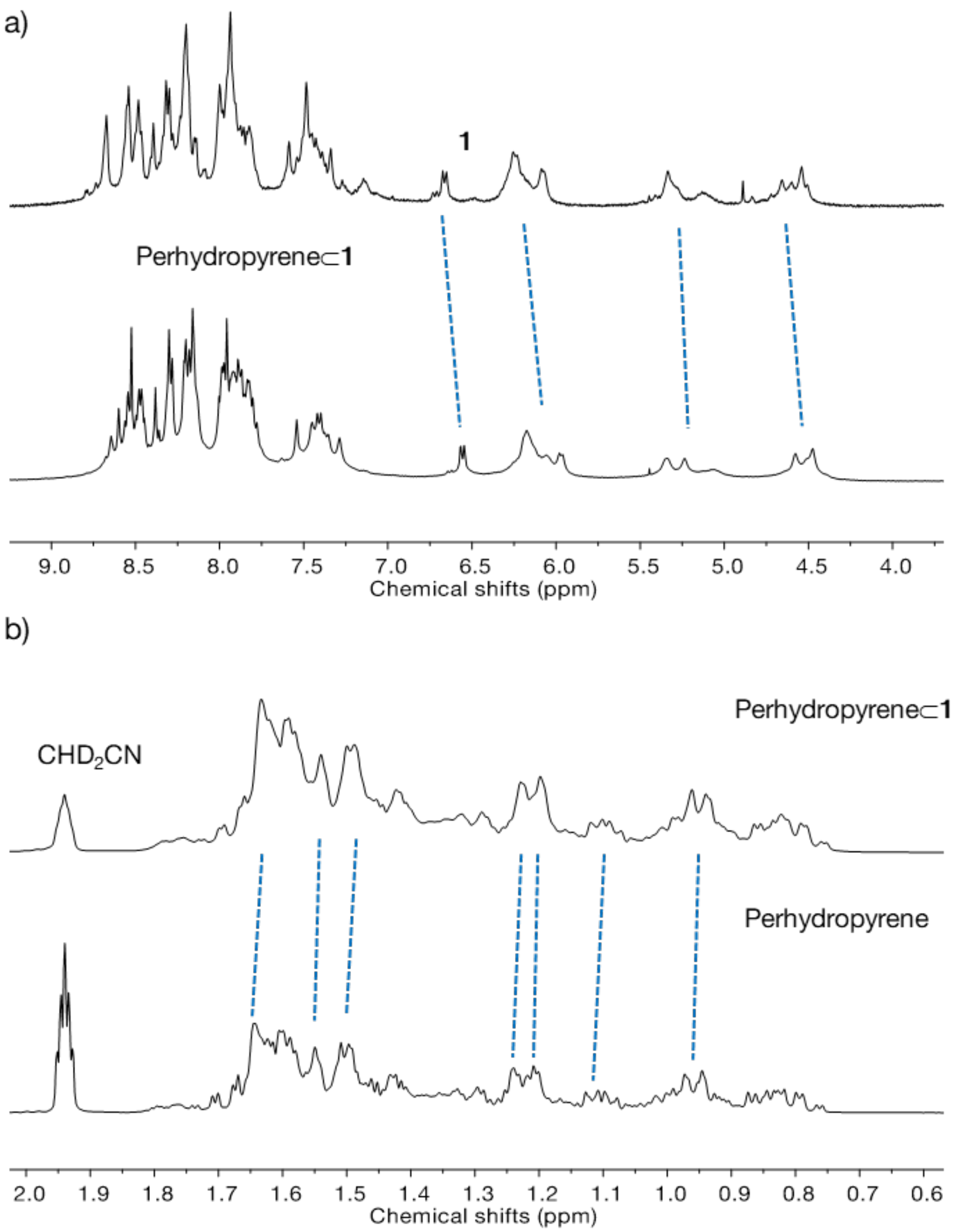

Supplementary Figure 28. ^{1}H NMR spectrum of perhydropyrene⊂**1**, (prepared with ca. 40 equiv. of perhydropyrene), with comparison to the ^{1}H NMR of a) empty cage **1** and b) the free guest (CD_3CN, 298 K, 400 MHz). Note that perhydropyrene consists of a mixture of stereoisomers. Note that a new set of host signals have emerged in addition to the signals of empty **1**. All guest signals have shifted. Representative new host signals and shifted guest signals are highlighted by blue dashed lines.

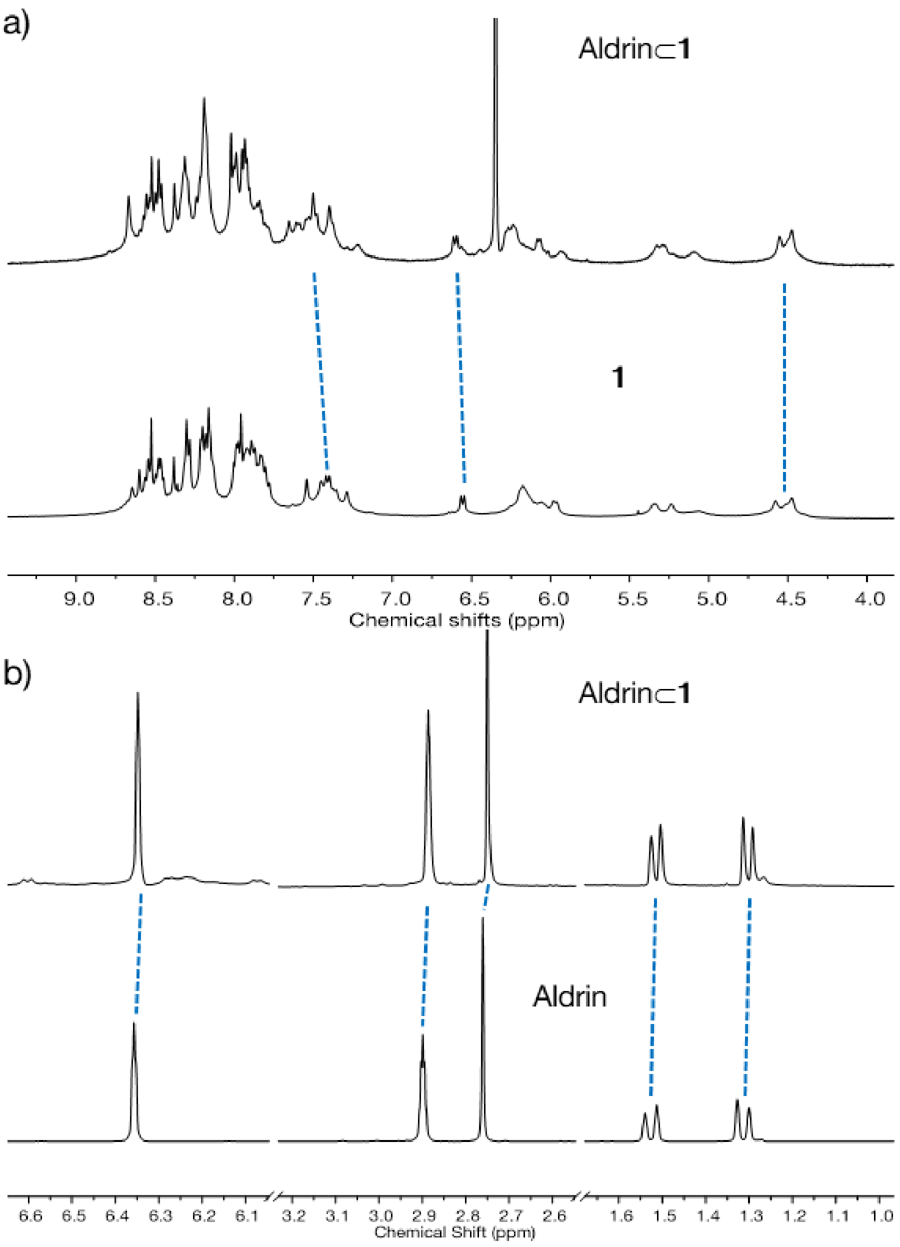

Supplementary Figure 29. ^{1}H NMR spectrum of aldrin⊂**1** (prepared with ca. 10 equiv. of aldrin), with comparison to the ^{1}H NMR of a) empty cage **1** and b) the free guest (CD_3CN, 298 K, 400 MHz for the spectra of free aldrin and **1** and 500 MHz for that of aldrin⊂1). Note that a new set of host signals have emerged in addition to the signals of empty 1. All the guest signals have shifted. Representative new host signals and shifted guest signals are highlighted by blue dashed lines.

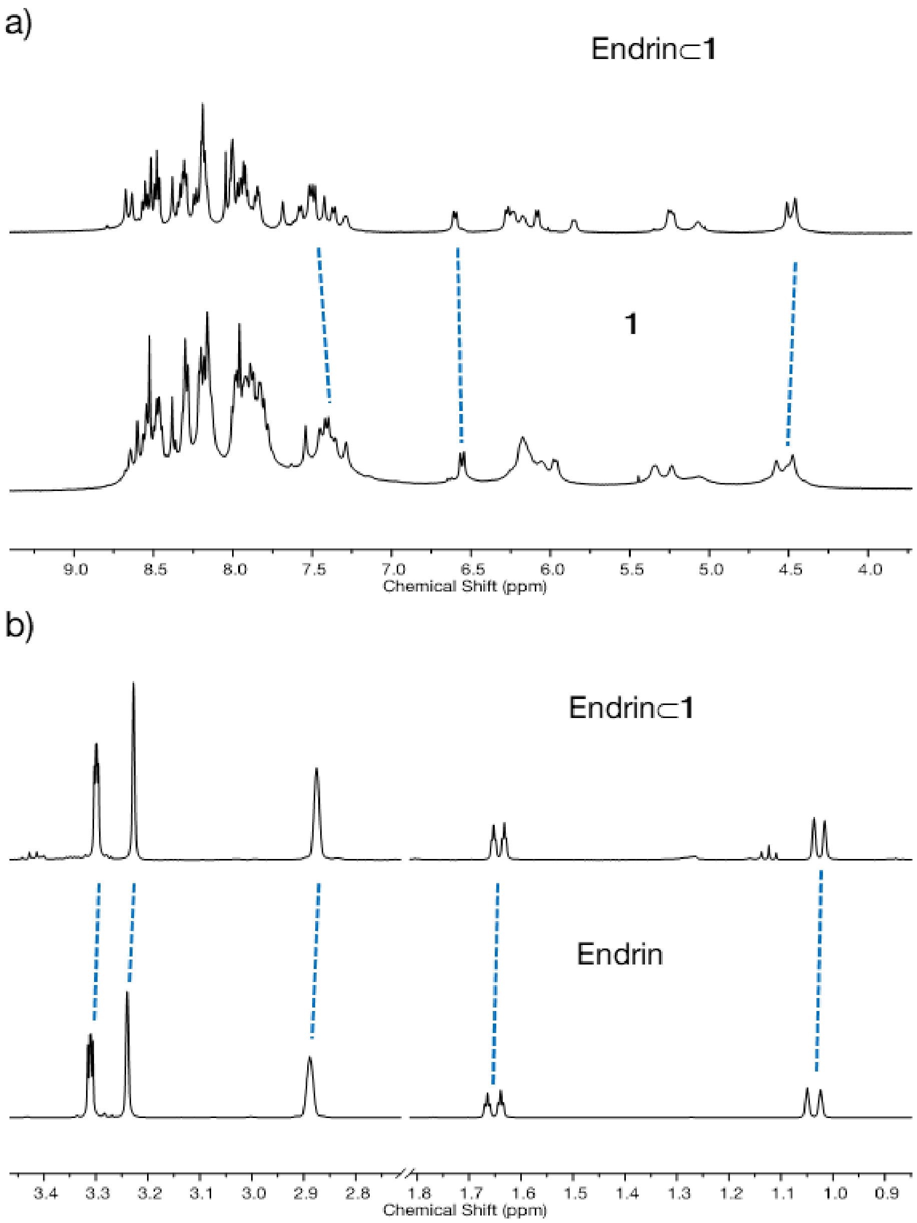

Supplementary Figure 30. ^{1}H NMR spectrum of endrin⊂**1** (prepared with ca. 10 equiv. of endrin), with comparison with the ^{1}H NMR of a) empty cage **1** and b) the free guest (CD_3CN, 298 K, 400 MHz for the spectra of free endrin and **1** and 500 MHz for that of endrin⊂**1**). Note that a new set of host signals have emerged in addition to the signals of empty **1**. All the guest signals have shifted. Representative new host signals and shifted guest signals are highlighted by blue dashed lines.

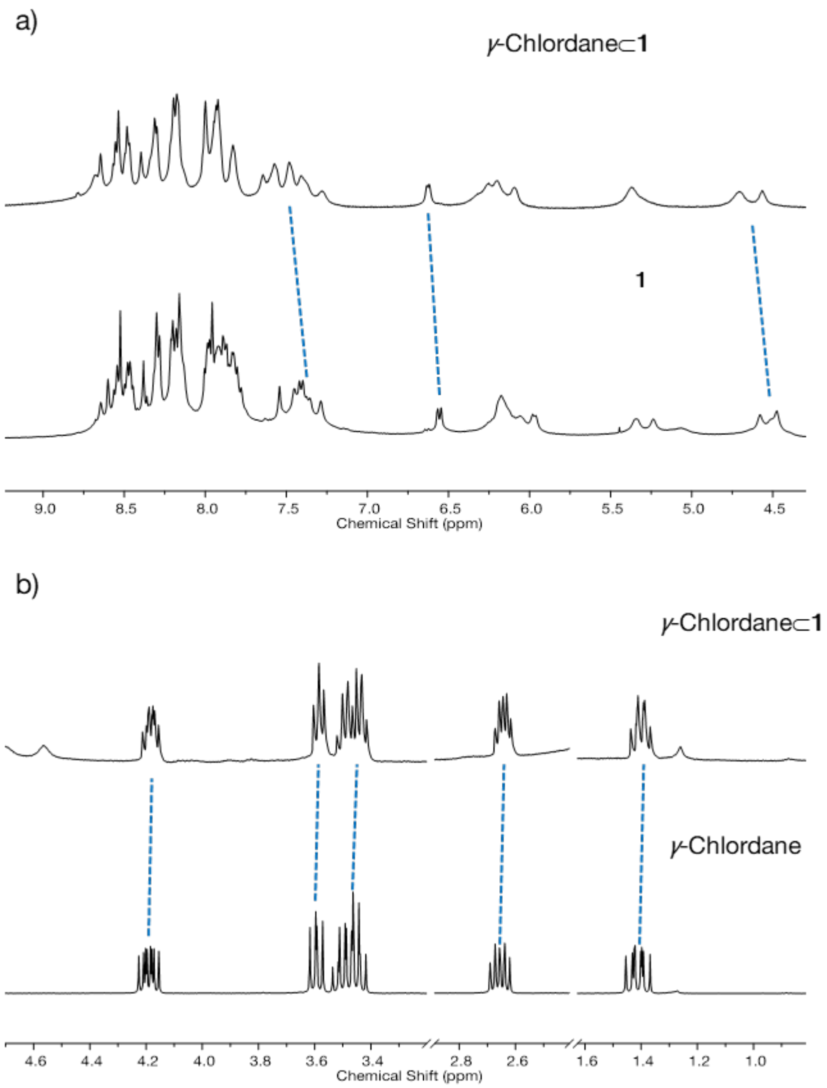

Supplementary Figure 31. ^{1}H NMR spectrum of γ-chlordane⊂**1** (prepared with ca. 10 equiv. of γ-chlordane), with comparison to the ^{1}H NMR of a) empty cage **1** and b) the free guest (CD_3CN, 298 K, 400 MHz for the spectra of free γ-chlordane and **1** and 500 MHz for that of γ-chlordane⊂**1**). A new set of host signals have emerged and all the guest signals have shifted. The shifts of guest signals are limited partly due to the high equivalents of guest used to convert **1** to γ-chlordane⊂**1** for clarity of the spectrum. Representative new host signals and shifted guest signals are highlighted by blue dashed lines.

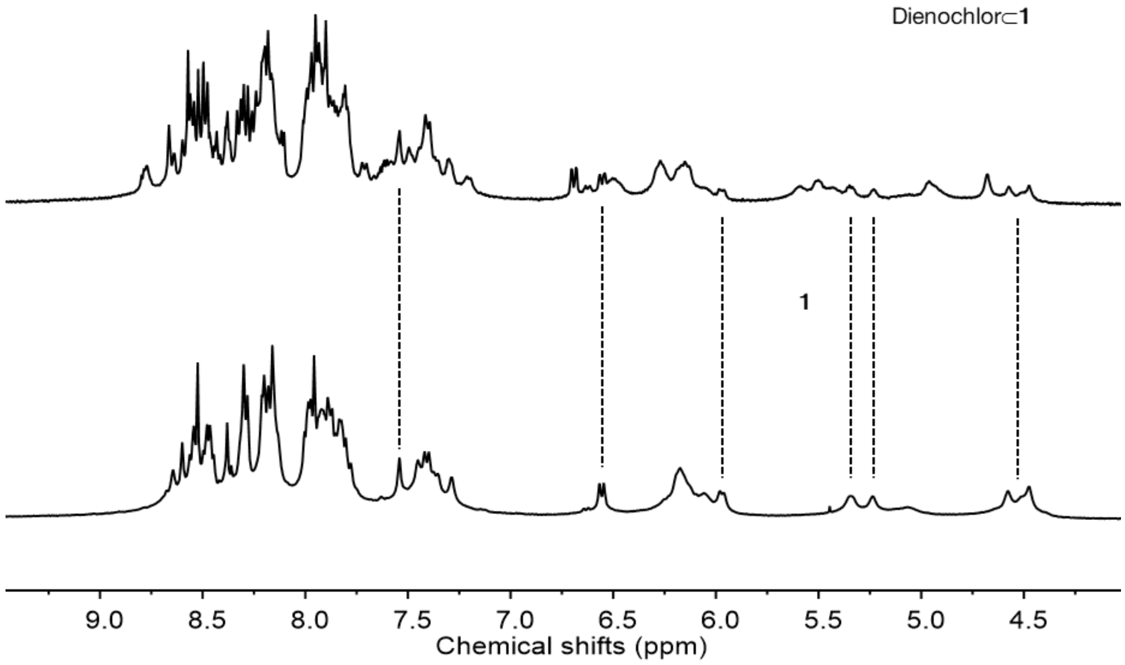

Supplementary Figure 32. ^{1}H NMR spectrum of dienochlor⊂**1** (prepared with ca. 10 equiv. of dienochlor), with comparison to the ^{1}H NMR of empty cage **1** and (CD_3CN, 298 K, 400 MHz for the spectra of free dienochlor and **1** and 500 MHz for that of dienochlor⊂**1**). Note that at least one new set of host signals have emerged in addition to the signals of empty **1**. Remaining signals for empty **1** are highlighted with black dashed lines.

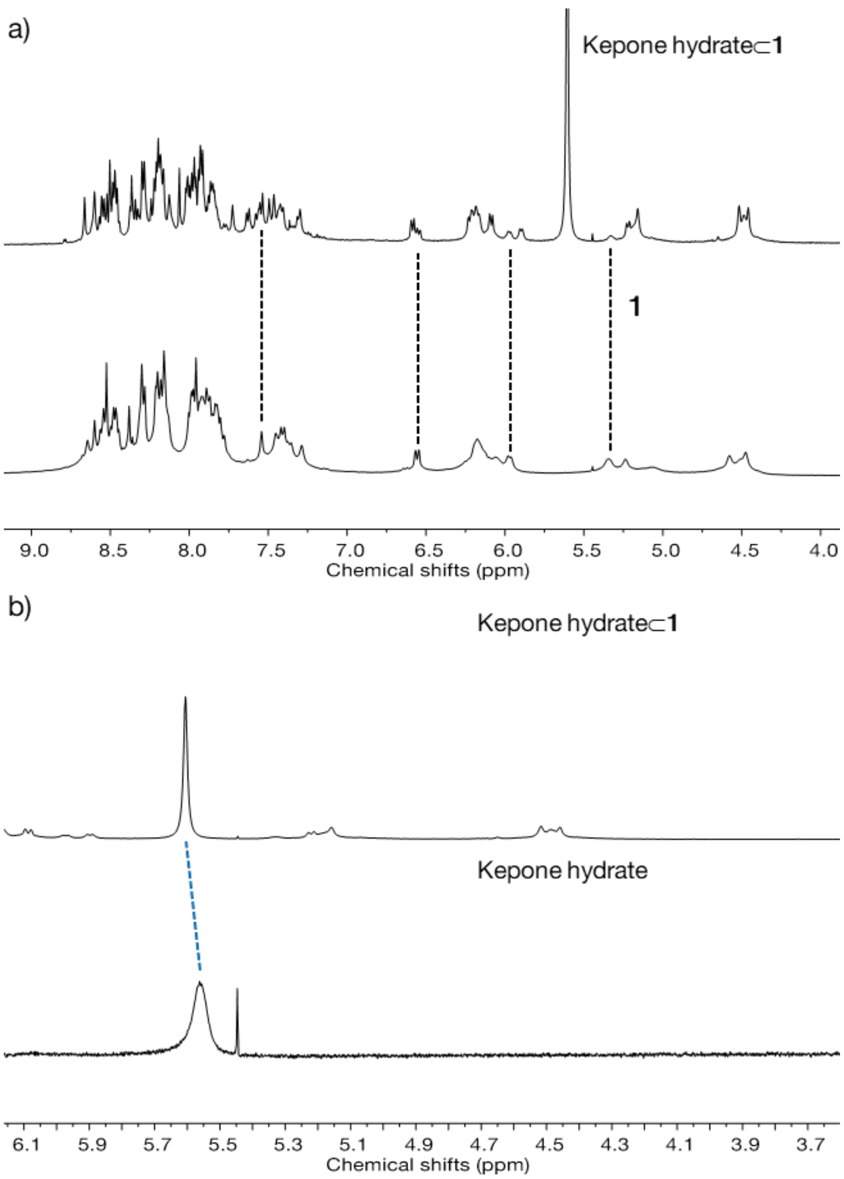

Supplementary Figure 33. ^{1}H NMR spectrum of Kepone hydrate⊂**1** (with ca. 20 equiv. of Kepone (a.k.a. Chlordecone) added)., with comparison with the ^{1}H NMR spectrum of empty cage **1** (CD_3CN, 298 K, 400 MHz for the spectrum of **1** and 500 MHz for that of Kepone hydrate⊂**1**). Under the experimental conditions Kepone hydrates and the hydrated product is bound by the cage, as revealed by mass spectrometry, no evidence was observed of Kepone bound by the cage. Note that a new set of host signals have emerged in addition to the signals of empty **1**. Representative remaining empty host peaks and the shifted guest peak are highlighted by black dashed lines and blue dashed lines, respectively.

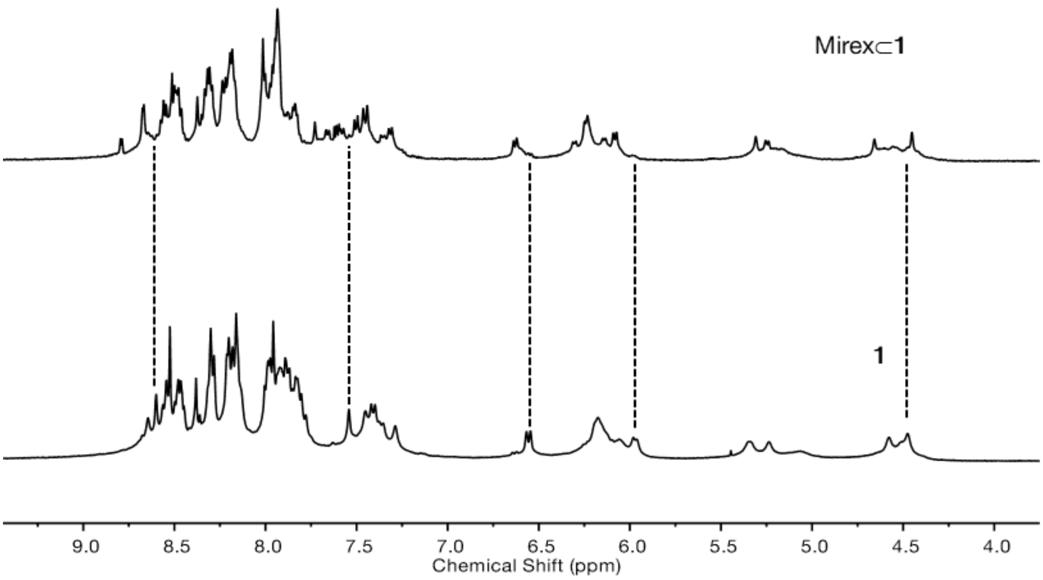

Supplementary Figure 34. ^{1}H NMR spectrum of mirex ⊂**1** (prepared with ca. 20 equiv. of mirex), with comparison to the ^{1}H NMR of empty cage **1** (CD_3CN, 298 K, 400 MHz for the spectrum of **1** and 500 MHz for that of mirex ⊂**1**). Note that at least one new set of host signals have emerged in addition to the signals of empty **1**. Remaining signals for empty **1** are highlighted with black dashed lines.

5.2 Mass spectra

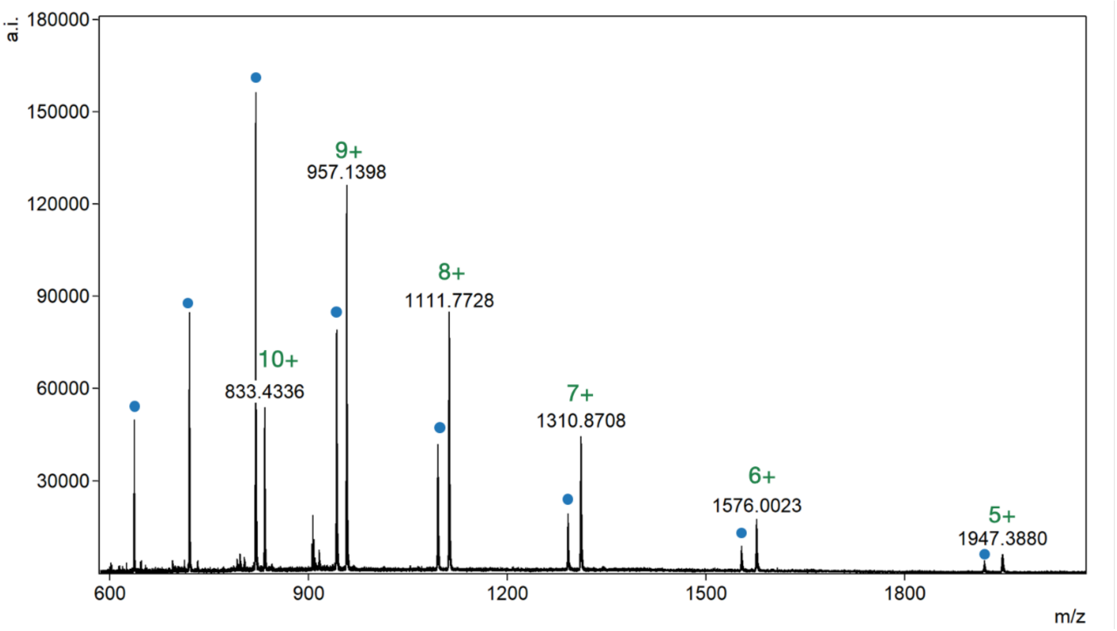

Supplementary Figure 35. ESI-HRMS spectrum of adamantane⊂**1** (with ca. 10 equiv. of adamantane). The peaks for the host-guest complex and for the empty cage are labelled with green text and blue dots respectively. Only signals corresponding to a 1:1 host guest complex have been observed.

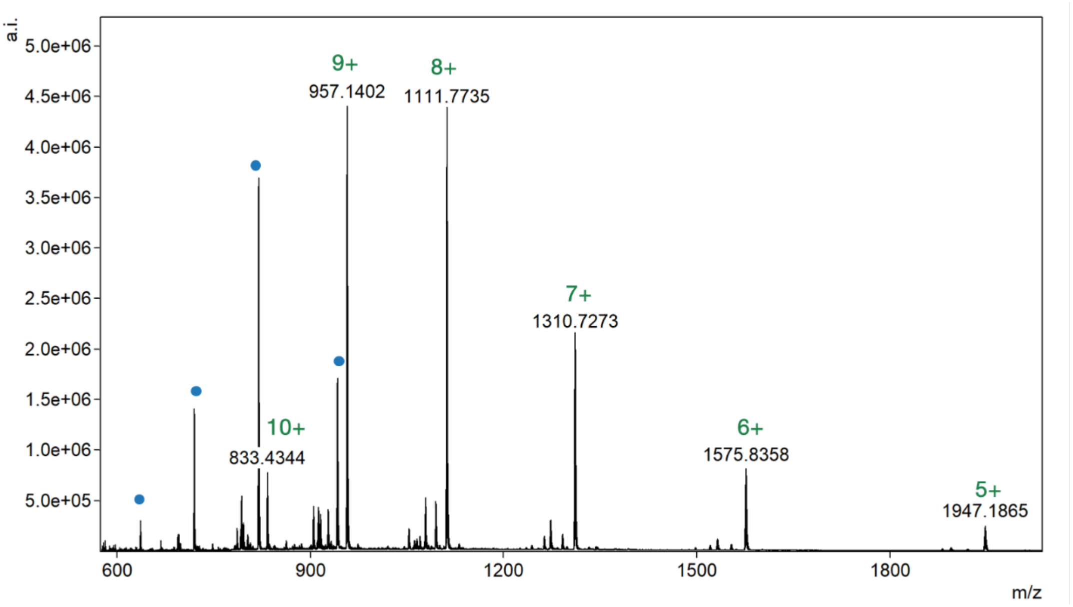

Supplementary Figure 36. ESI-HRMS spectrum of *α*-pinene⊂**1** (with ca. 15 equiv. of adamantane). The peaks for the host-guest complex and for the empty cage are labelled with green text and blue dots respectively. Only signals corresponding to a 1:1 host guest complex have been observed.

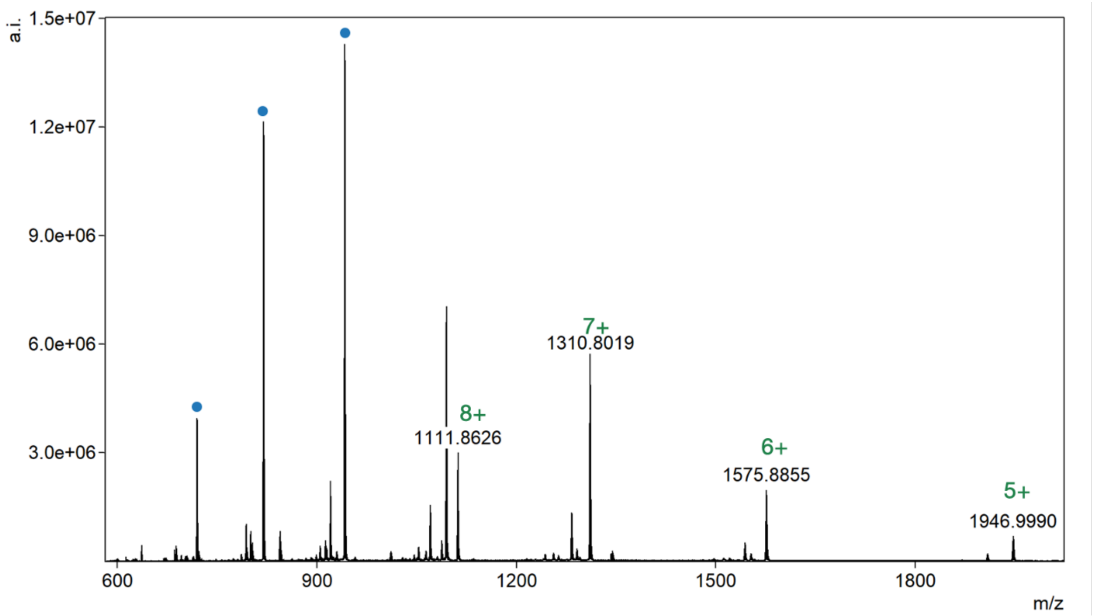

Supplementary Figure 37. ESI-HRMS spectrum of *trans*-decalin⊂**1** (with ca. 15 equiv. of *trans*-decalin). The peaks for the host-guest complex and for the empty cage are labelled with green text and blue dots respectively. Only signals corresponding to a 1:1 host guest complex were observed.

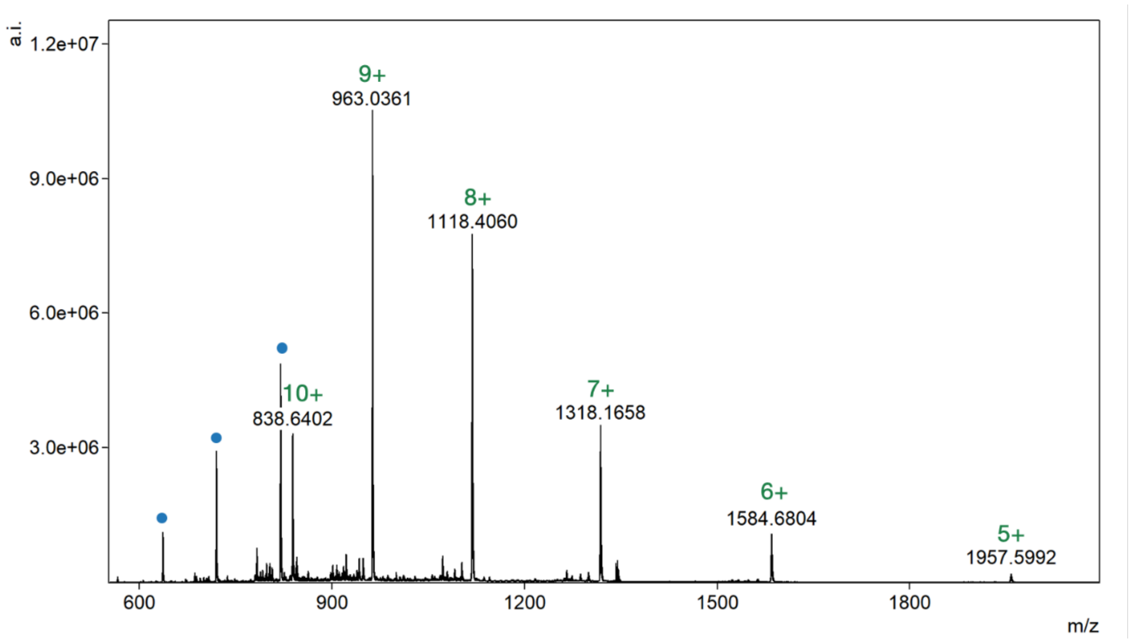

Supplementary Figure 38. ESI-HRMS spectrum of diamantane⊂**1** (with ca. 10 equiv. of diamantane). The peaks for the host-guest complex and for the empty cage are labelled with green text and blue dots respectively. Only signals corresponding to a 1:1 host guest complex were observed.

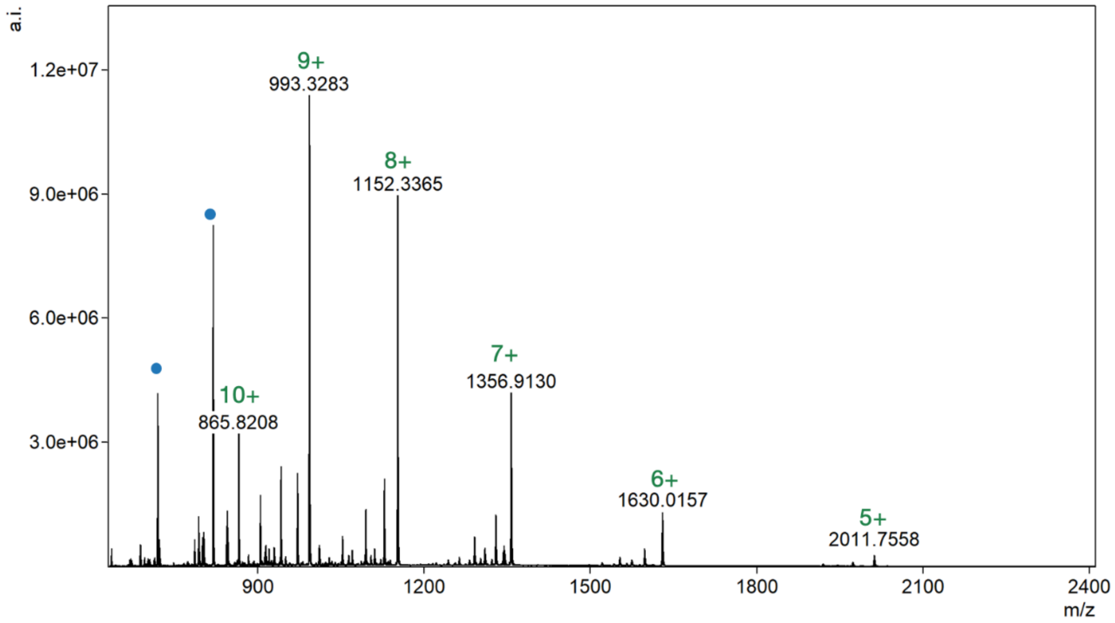

Supplementary Figure 39. ESI-HRMS spectrum of *trans*-perfluorodecalin⊂**1** (prepared with ca. 20 equiv. of *trans*-perfluorodecalin, precipitated by addition of Et_2O and redissolved). The peaks for the host-guest complex and for the empty cage are labelled with green text and blue dots respectively. Only signals corresponding to a 1:1 host guest complex were observed.

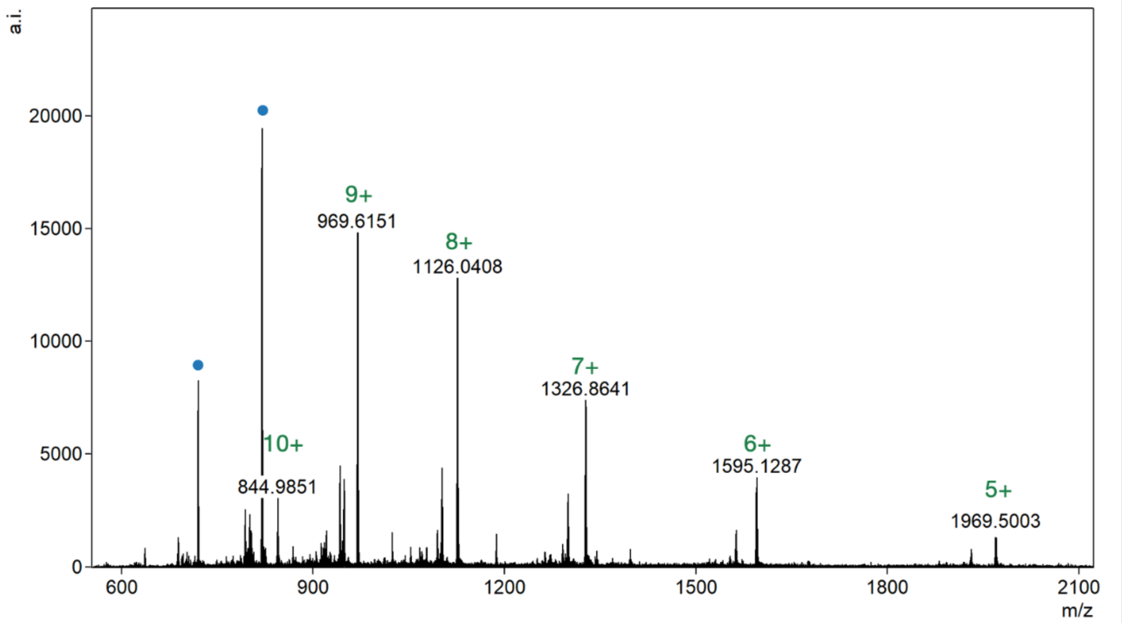

Supplementary Figure 40. ESI-HRMS spectrum of corannulene ⊂**1** (prepared with ca. 20 equiv. of corannulene). The peaks for the host-guest complex and for the empty cage are labelled with green text and blue dots respectively. Only signals corresponding to a 1:1 host guest complex were observed.

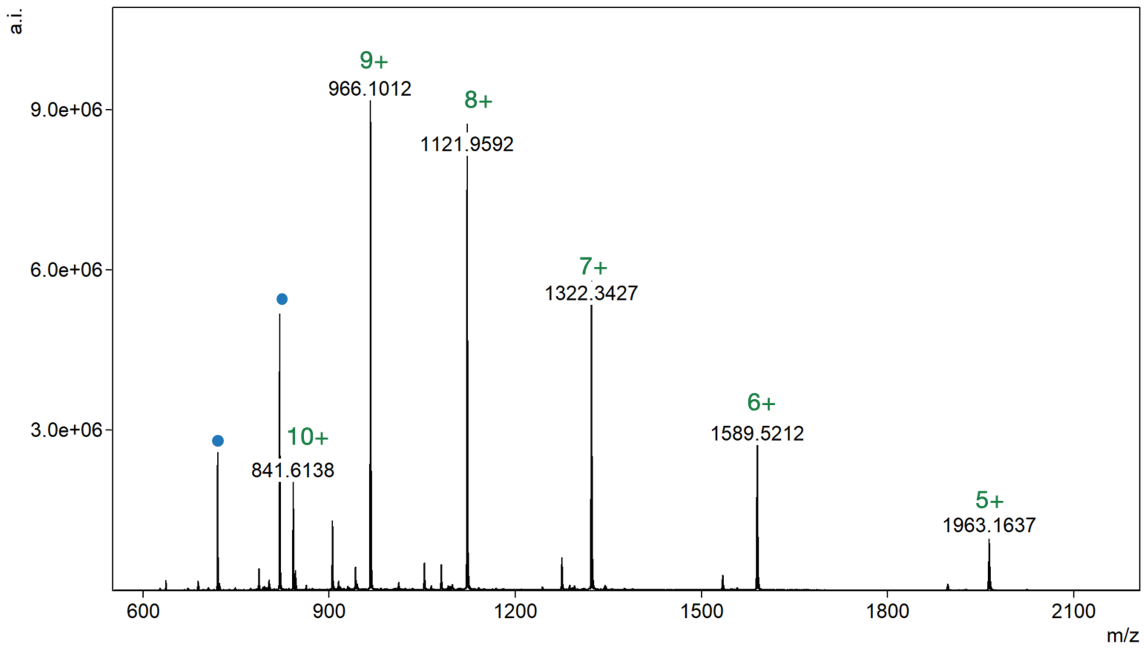

Supplementary Figure 41. ESI-HRMS spectrum of perhydropyrene⊂**1** (prepared with ca. 40 equiv. of perhydropyrene). The peaks for the host-guest complex and for the empty cage are labelled with green text and blue dots respectively. Only signals corresponding to a 1:1 host guest complex were observed.

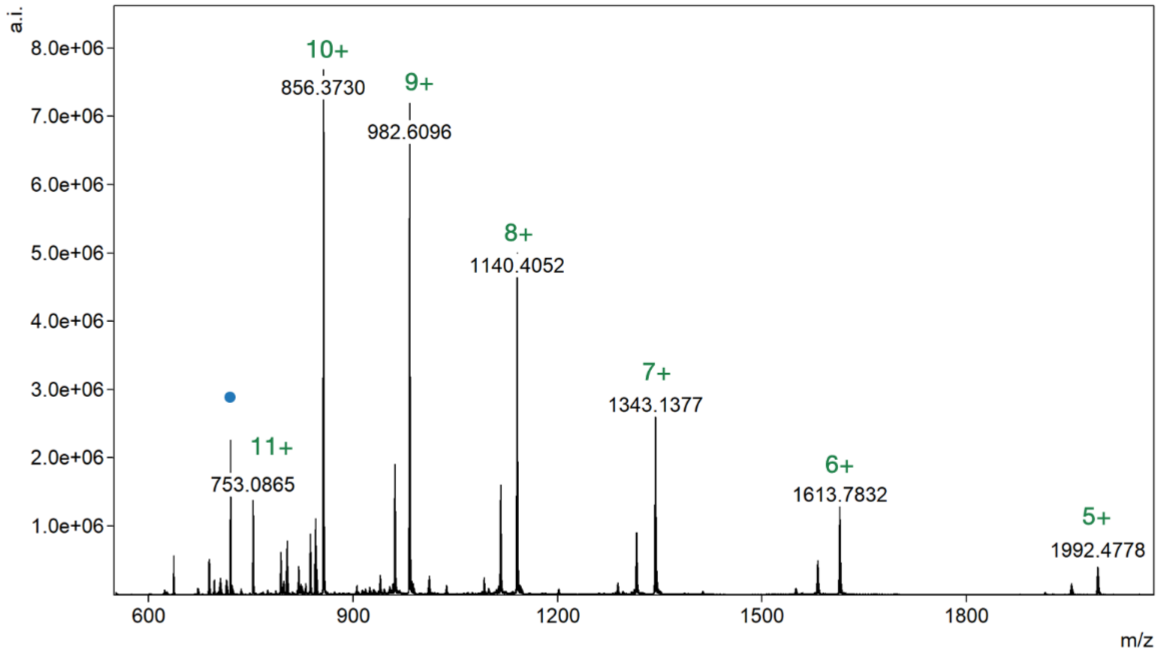

Supplementary Figure 42. ESI-HRMS spectrum of aldrin⊂**1** (prepared with ca. 10 equiv. of aldrin). The peaks for the host-guest complex and for the empty cage are labelled with green text and a blue dot respectively. Only signals corresponding to a 1:1 host guest complex were observed.

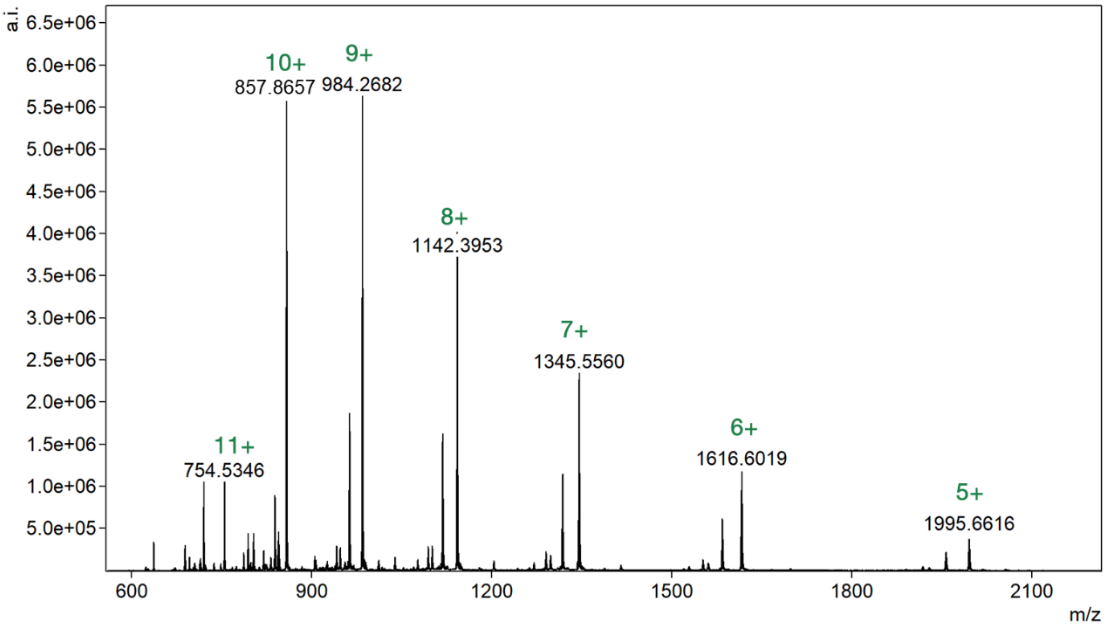

Supplementary Figure 43. ESI-HRMS spectrum of endrin⊂**1** (prepared with ca. 10 equiv. of endrin). The peaks for the host-guest complex and for the empty cage are labelled with green text. Only signals corresponding to a 1:1 host guest complex were observed, with no signals detected corresponding to empty **1**.

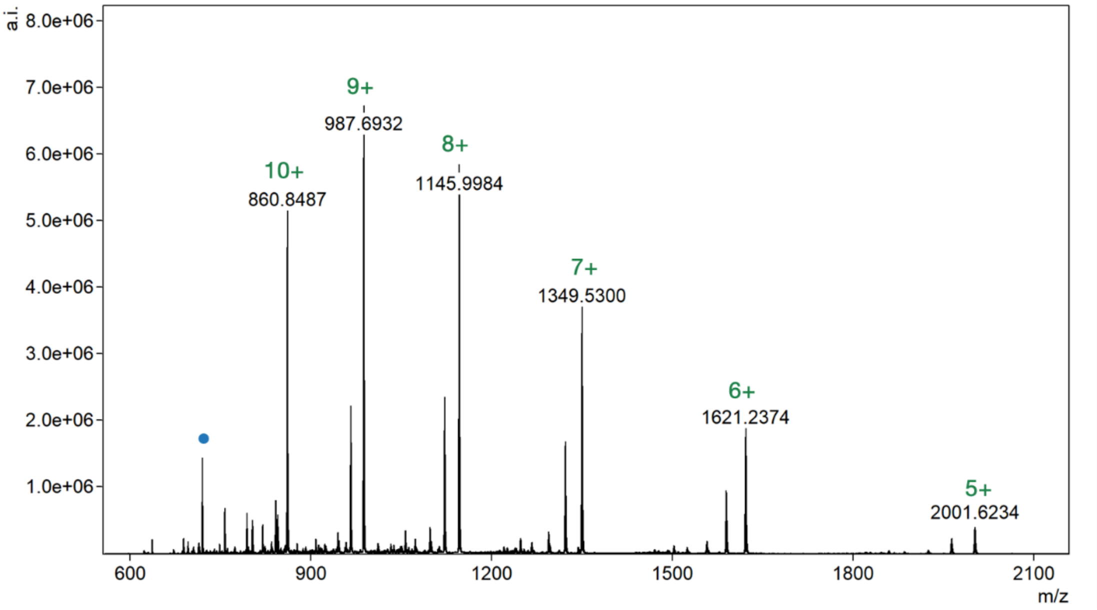

Supplementary Figure 44. ESI-HRMS spectrum of *γ*-chlordane⊂**1** (prepared with ca. 10 equiv. of *γ*-chlordane). The peaks for the host-guest complex and for the empty cage are labelled with green text and blue dots respectively. Only signals corresponding to a 1:1 host guest complex were observed.

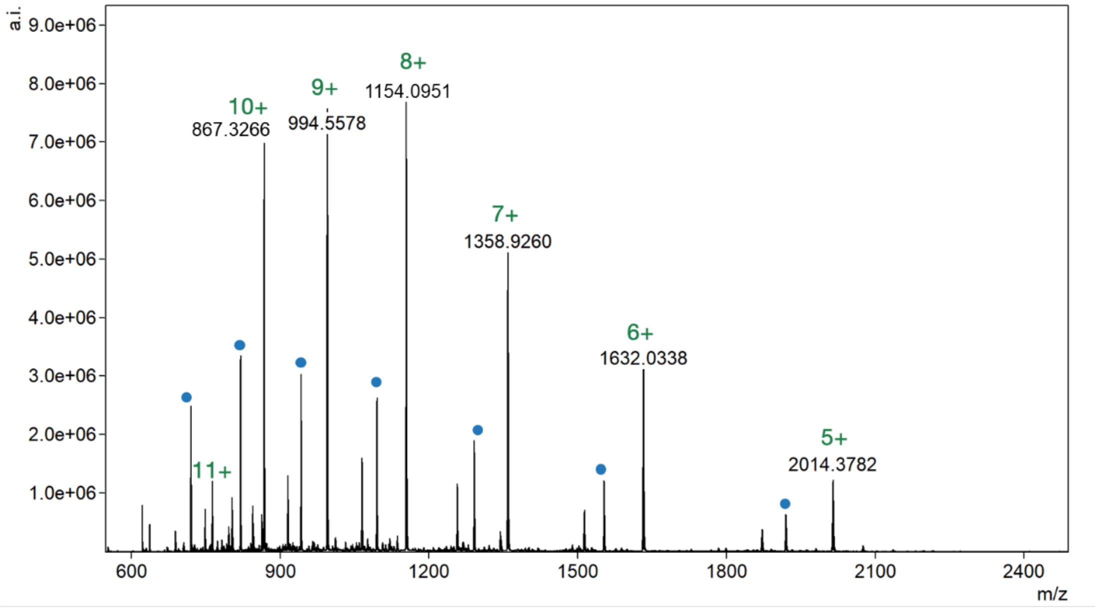

Supplementary Figure 45. ESI-HRMS spectrum of dienochlor⊂**1** (prepared with ca. 10 equiv. of dienochlor). The peaks for the host-guest complex and for the empty cage are labelled with green text and blue dots respectively. Only signals corresponding to a 1:1 host guest complex were observed.

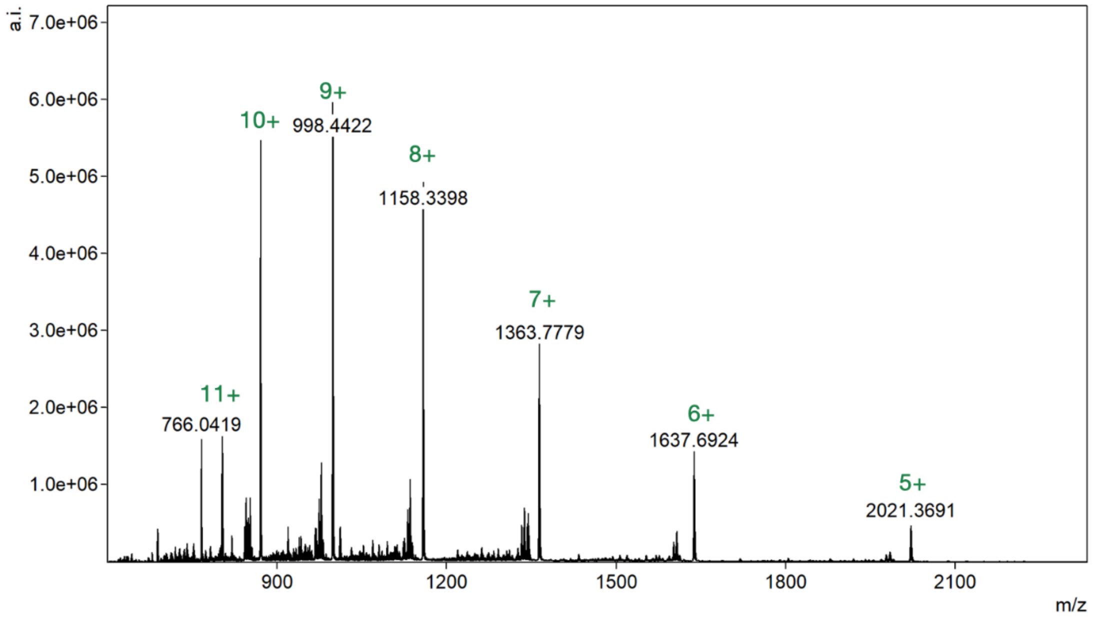

Supplementary Figure 46. ESI-HRMS spectrum of Kepone hydrate⊂**1**. (20 equiv of Kepone a.k.a. chlordecone) was added to prepare the host-guest complex, only the hydrated product was found to be bound by the cage, while no evidence of Kepone bound by the cage) The peaks for the host-guest complex are labelled with green text. Only signals corresponding to a 1:1 host guest complex were observed, with no

signals detected corresponding to empty **1**.

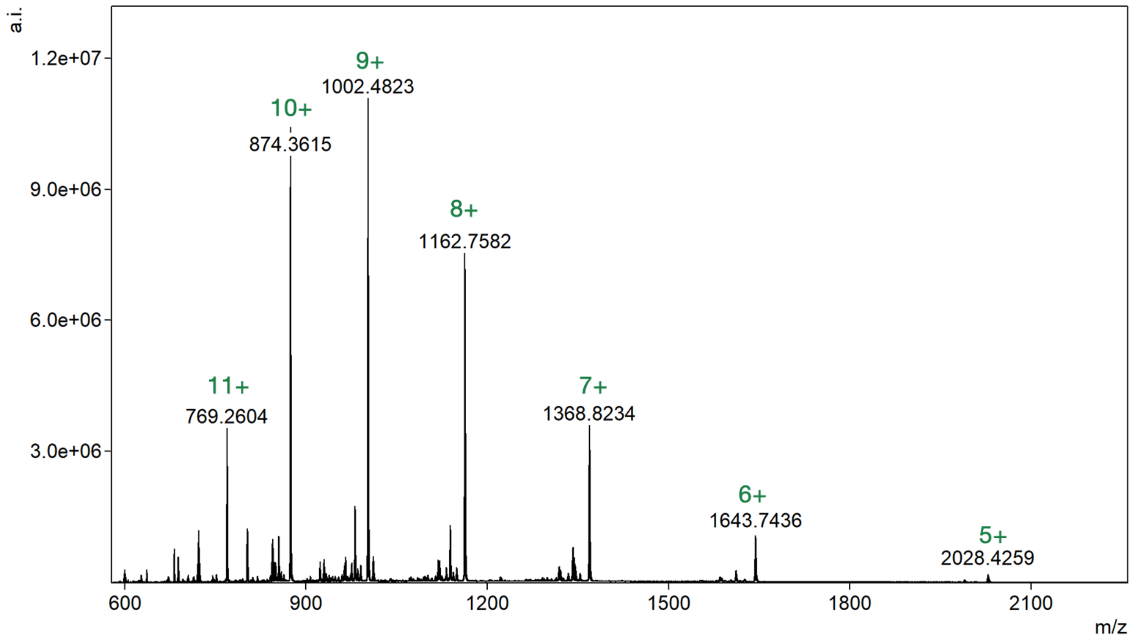

Supplementary Figure 47. ESI-HRMS spectrum of mirex⊂**1** (prepared with ca. 20 equiv. of mirex). The peaks for the host-guest complex and for the empty cage are labelled with green text. Only signals corresponding to a 1:1 host guest complex were observed, with no signals detected corresponding to empty **1**..

5.2 Anionic guests

Three anionic borate guests were considered in the guest binding studies of **1**. While it is not possible to characterize the binding by mass spectrometry methods, NMR titrations have been performed to determine the strength and binding stoichiometries of these guest molecules in **1**. In NMR titration experiments, 1,4-dimethoxybenzene was used as an internal standard, and the binding isotherm generated was fitted using BindFit v0.5.[17] The equations used for these analyses had been summarized in the review by Thordarson.[18] The covariance of the fit (variance of the residuals divided by the variance in the data), along with a visual inspection of the residuals from the fit, was used to conclude that a 1:1 binding stoichiometry best describes the binding of **1** and all anionic guests in this study.

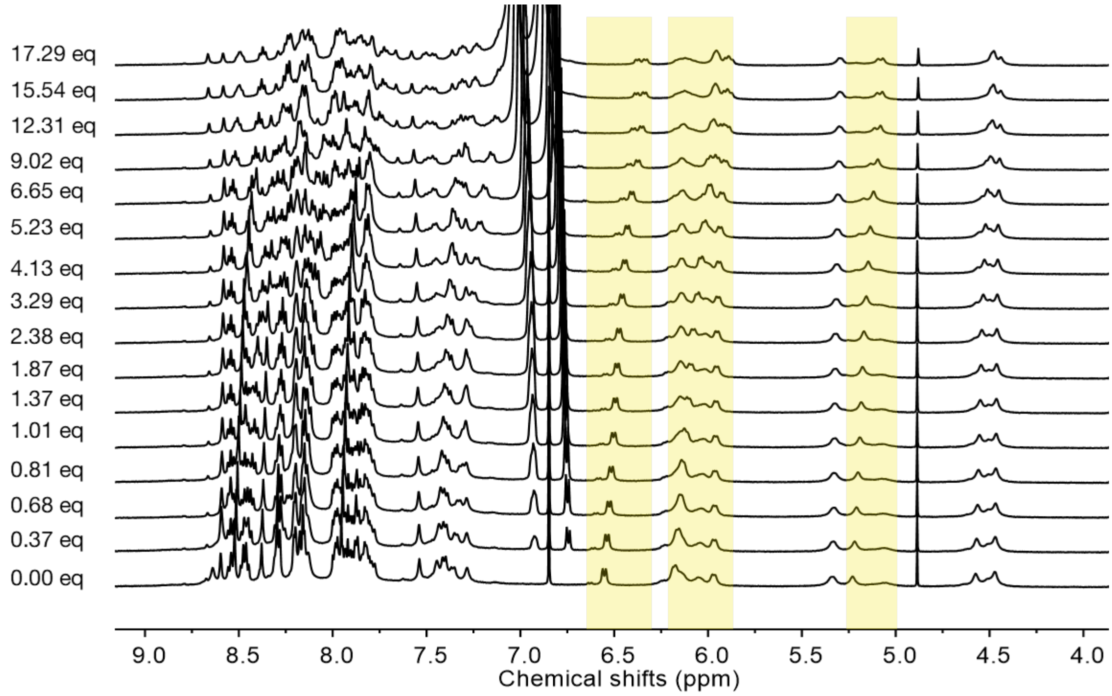

Supplementary Figure 48. Stack plot of the ^{1}H NMR spectra generated in the NMR titration of potassium *tetrakis*(4-chlorophenyl)borate to **1**, with protons with the most notable shifts highlighted in yellow. In this titration the concentration of **1** was calibrated as 0.93 mM, while a 1.00 M solution of the guest in CD_3CN was used.

Parameter (bounds)	Optimised	Error	Initial
K (0 → ∞)	260.53 M^{-1}	± 5.3260 %	100.00 M^{-1}

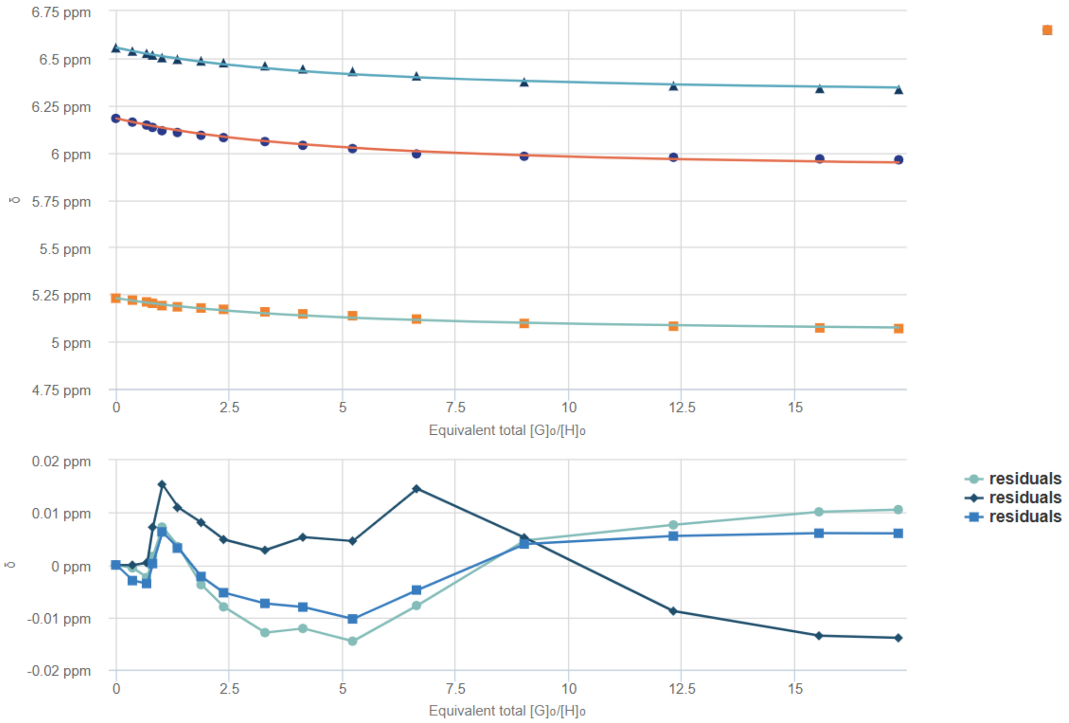

Supplementary Figure 49. NMR titration isotherm (1:1 system) fitted to the chemical shifts of the proton signals at 6.55, 6.18 and 5.23 ppm vs. the equivalents of potassium *tetrakis*(4-chlorophenyl)borate added to determine the binding affinity (K_a = 260±14 M^{-1}, top right); and the residual plot from the fit (bottom).

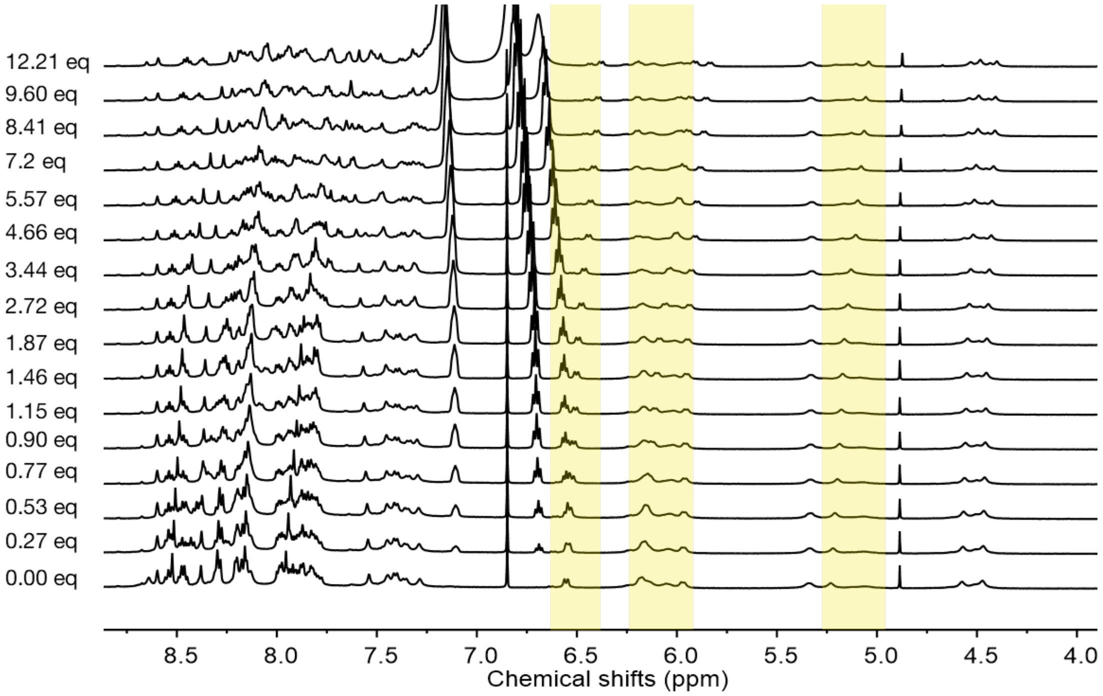

Supplementary Figure 50. Stack plot of the ^{1}H NMR spectra generated in the NMR titration of sodium tetraphenylborate to **1**, with protons with the most notable shifts highlighted in yellow. In this titration the concentration of **1** was calibrated as 0.95 mM, while a 1.00 M solution of sodium tetraphenylborate in CD_3CN was used.

Parameter (bounds)	Optimised	Error	Initial
K (0 → ∞)	207.08 M^{-1}	± 2.1454 %	100.00 M^{-1}

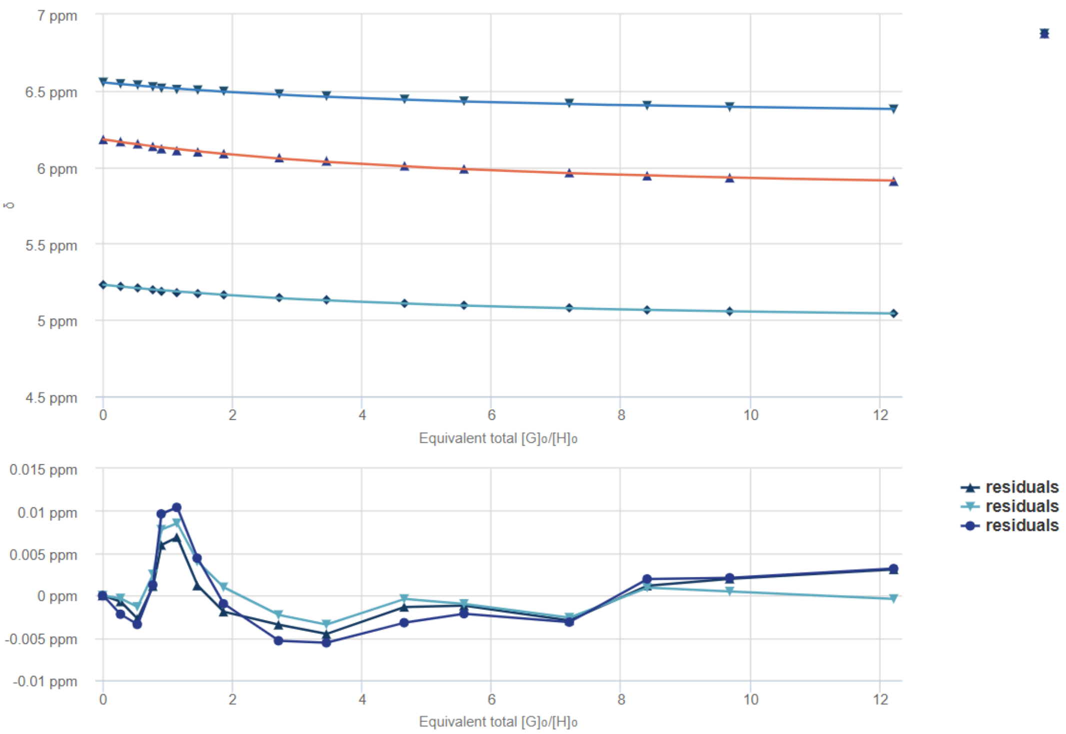

Supplementary Figure 51. NMR titration isotherm (1:1 system) fitted to the chemical shift of the proton signals at 6.55, 6.18 and 5.23 ppm vs. the equivalents of sodium tetraphenylborate added to determine the binding affinity (K_a = 207±4 M^{-1}, top right); and the residual plot from the fit (bottom).

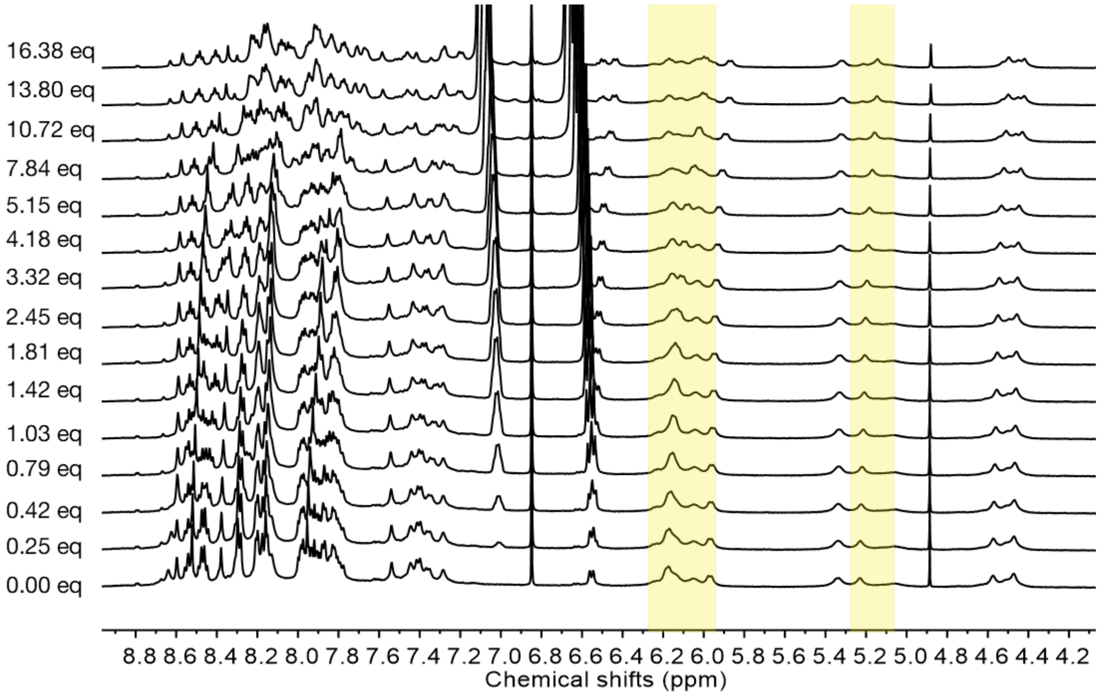

Supplementary Figure 52. Stack plot of the ^1H NMR spectra generated in the NMR titration of sodium *tetrakis*(4-fluorophenyl)borate to **1**, with protons with the most notable shifts highlighted in yellow. In this titration the concentration of **1** was calibrated as 0.94 mM, while a 1.00 M solution of the guest in CD_3CN was used.

Parameter (bounds)	Optimised	Error	Initial
K (0 → ∞)	117.77 M^{-1}	± 3.4130 %	100.00 M^{-1}

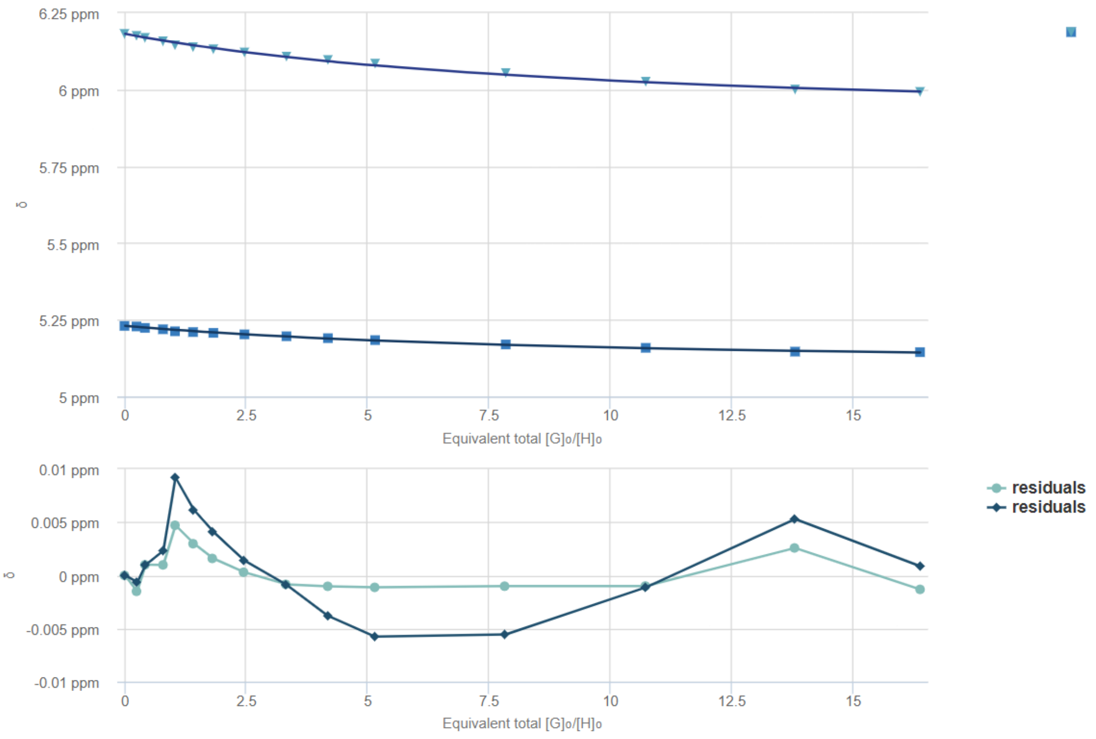

Supplementary Figure 53. NMR titration isotherm (1:1 system) fitted to the chemical shift of the proton signals at 6.55, 6.18 and 5.23 ppm vs. the equivalents of sodium *tetrakis*(4-fluorophenyl)borate added to determine the binding affinity (K_a = 118±4 M^{-1}, top); and the residual plot from the fit (bottom).

5.3 Further NMR evidence of the face-flipping of 1 upon guest binding

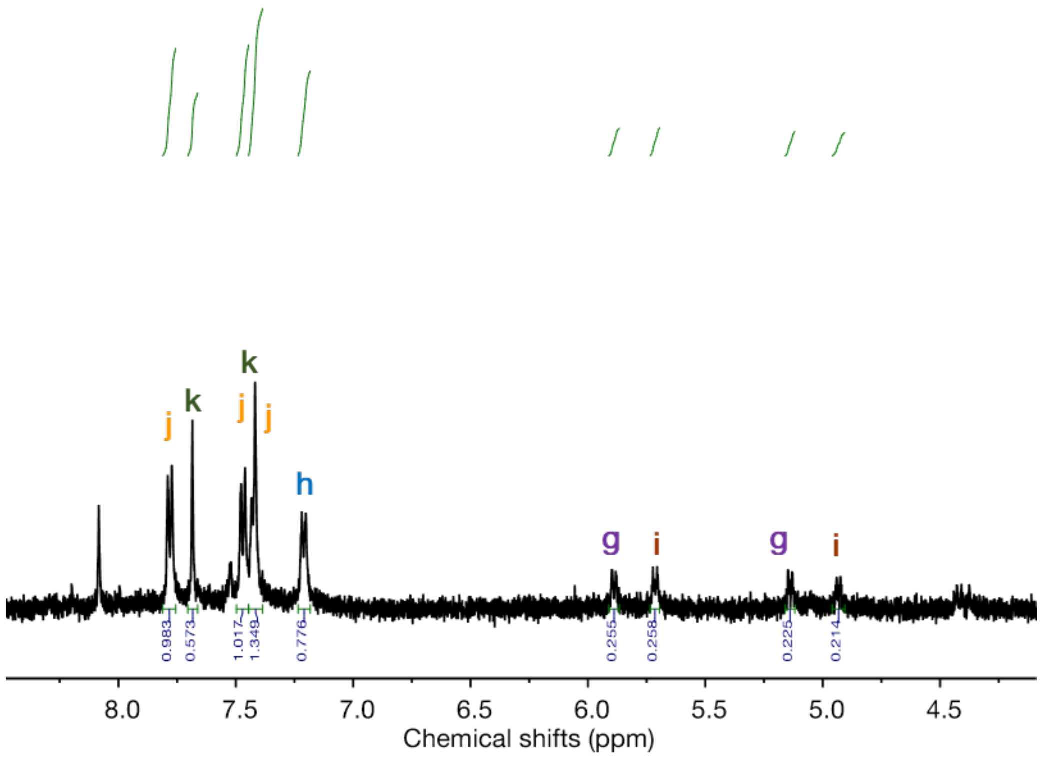

Supplementary Figure 54. Integration of the 1D NOESY spectrum in Fig. 4e as an approximate comparison between the population of *endo* and *exo* faces: the sum of integration of the **j**, **k** and **i** protons (4.394), indicating *exo* faces, exceeds the sum of integration of protons **g** and **h** (1.256), that indicate *endo* faces.

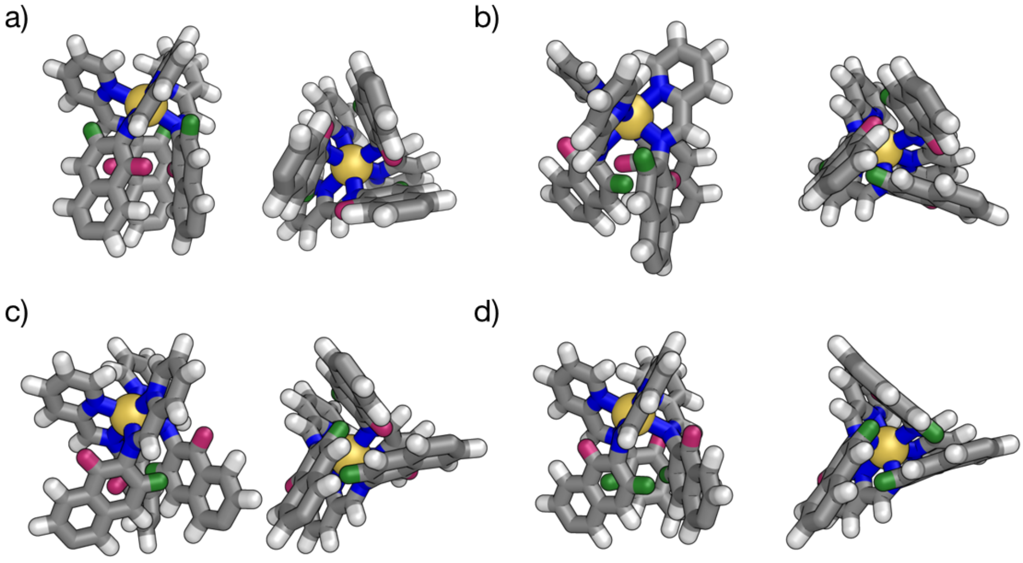

Supplementary Figure 55. Visualization of the relative positions of protons **f** (colored pink) and **i** (colored green) within a vertex of cube **1**. a) a vertex with three concave faces, all the proton **f**s are in close proximity to one another. Vertices with b) one c)

two d) three convex faces, as the naphthalenes rotate protons **f** and **i** start to gain adjacency instead of the **f**s.

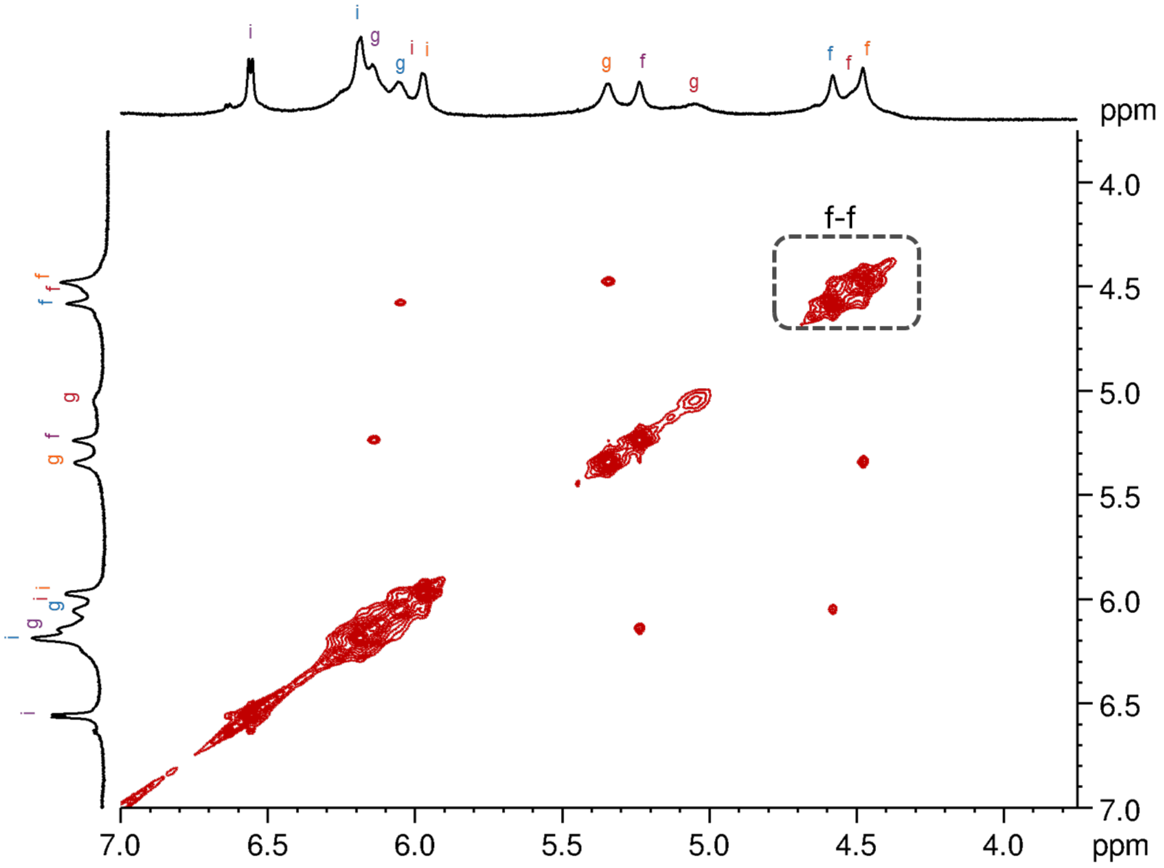

Supplementary Figure 56. Selected region of the ^1H-^1H NOESY spectrum (700 MHz, CD_3CN, 298K) of **1**. Three NOE cross peaks between the protons **f**s have been identified.

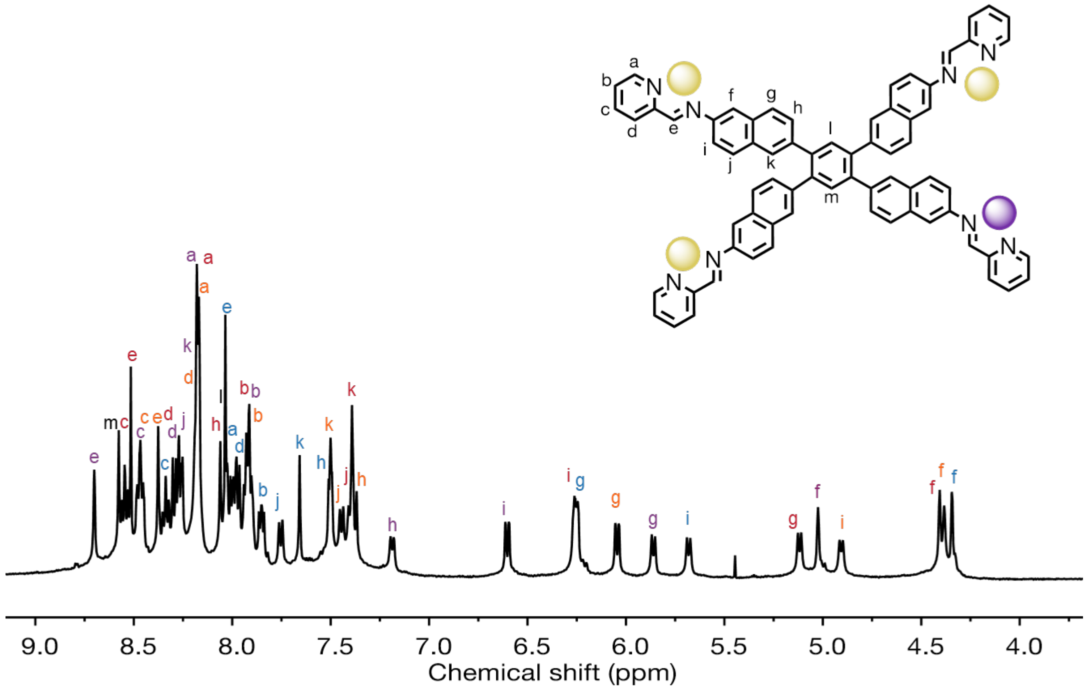

Supplementary Figure 57. Aromatic region of the ^{1}H NMR spectrum (500 MHz, CD_3CN, 298 K) of diamantane⊂**1** with signal assignments. The signals associated with each distinct ligand arm could be distinguished and marked with different colors; however, it was not possible to definitively attribute each set to a particular ligand arm within the structure.

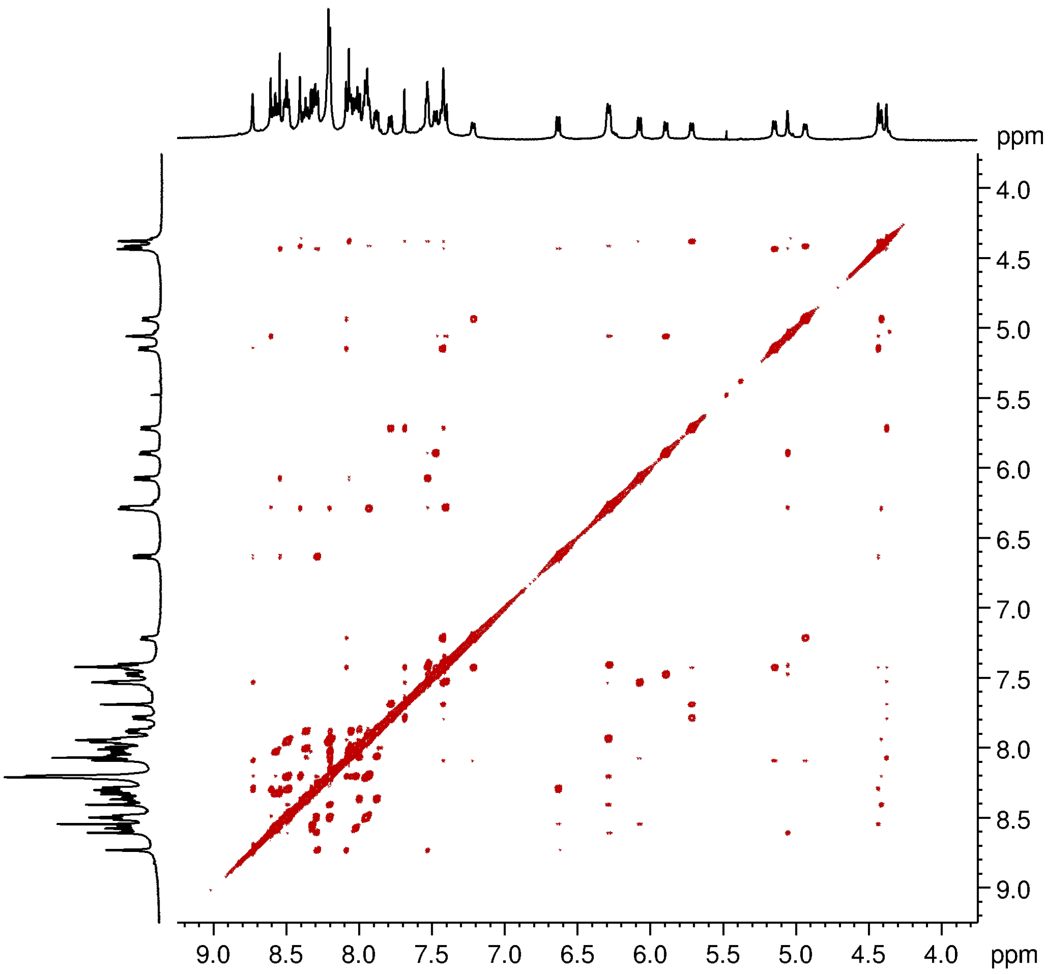

Supplementary Figure 58. Aromatic region of the ^{1}H-^{1}H NOESY spectrum (500 MHz, CD_3CN, 298K) of diamantane⊂**1**.

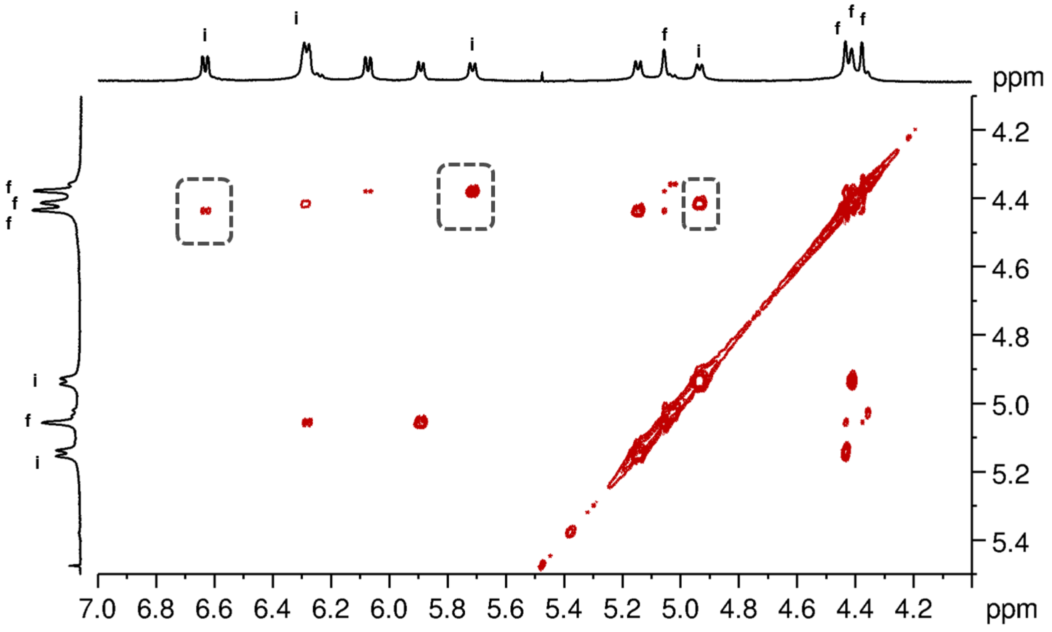

Supplementary Figure 59. Selected region of the ^1H-^1H NOESY spectrum (500 MHz, CD_3CN, 298K) of diamantane⊂**1**. Three NOE cross peaks between the protons **f**-**i** have been identified, which indicates the rotation of the naphthalene units.

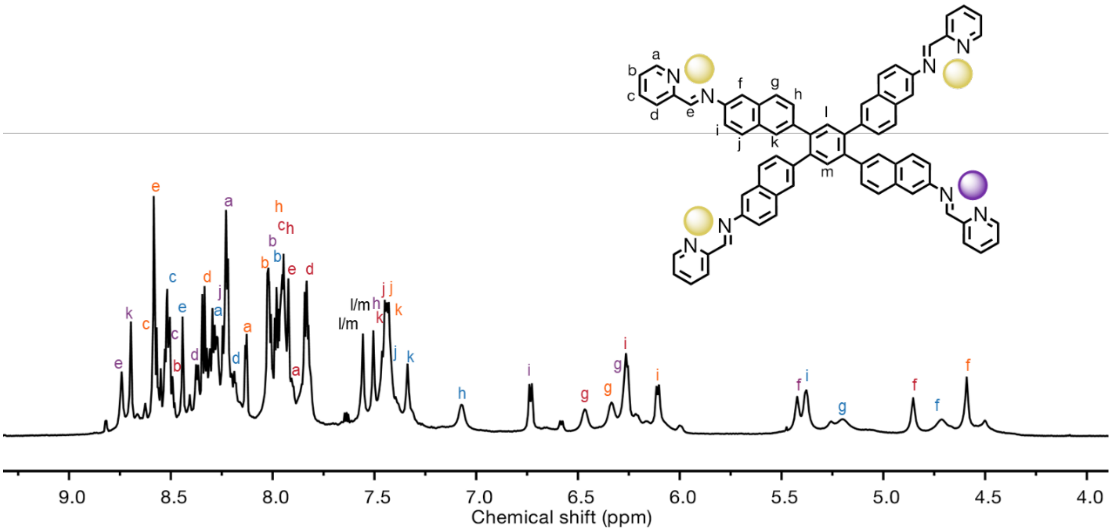

Supplementary Figure 60. Aromatic region of the ^1H NMR spectrum (500 MHz, CD_3CN, 298 K) of *trans*-Perfluorodecalin⊂**1** with signal assignments. More than one set of host signals are present in this case, only the most intense set of signals have been assigned. The signals associated with each distinct ligand arm could be distinguished and marked with different colors; however, it was not possible to definitively attribute each set to a particular ligand arm within the structure.

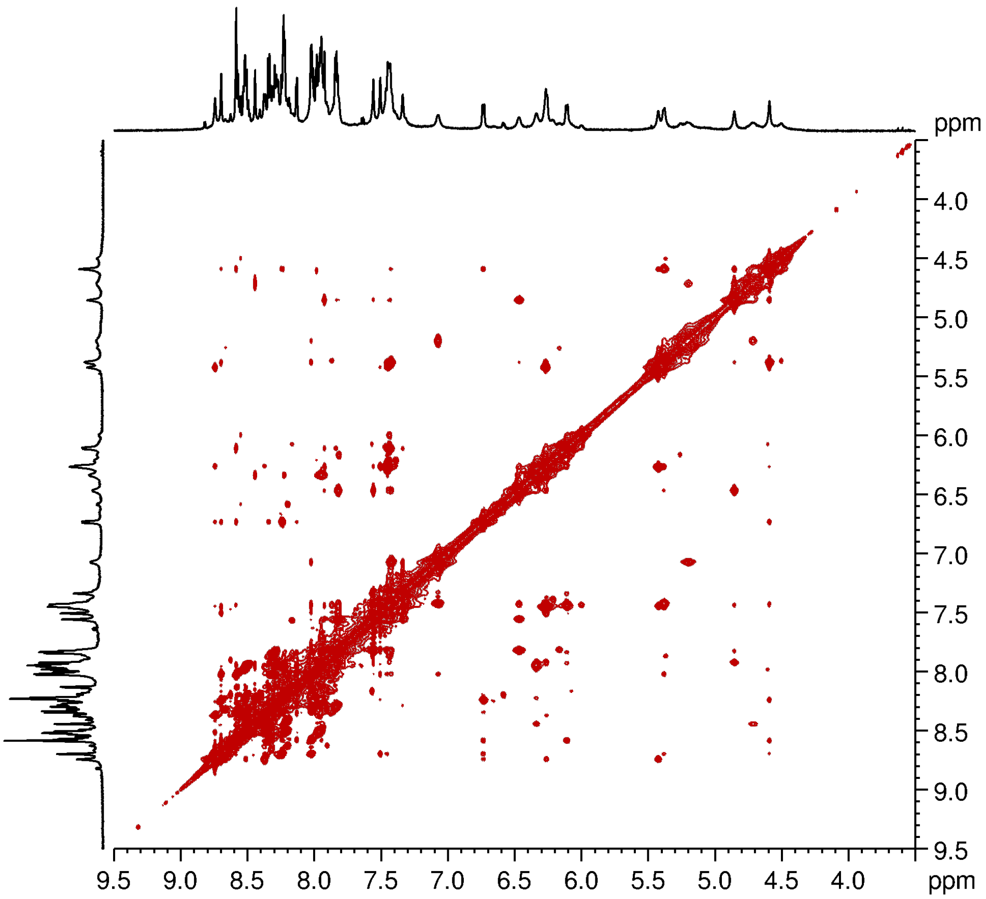

Supplementary Figure 61. Aromatic region of the ^{1}H-^{1}H NOESY spectrum (500 MHz, CD_3CN, 298K) of *trans*-Perfluorodecalin⊂**1**.

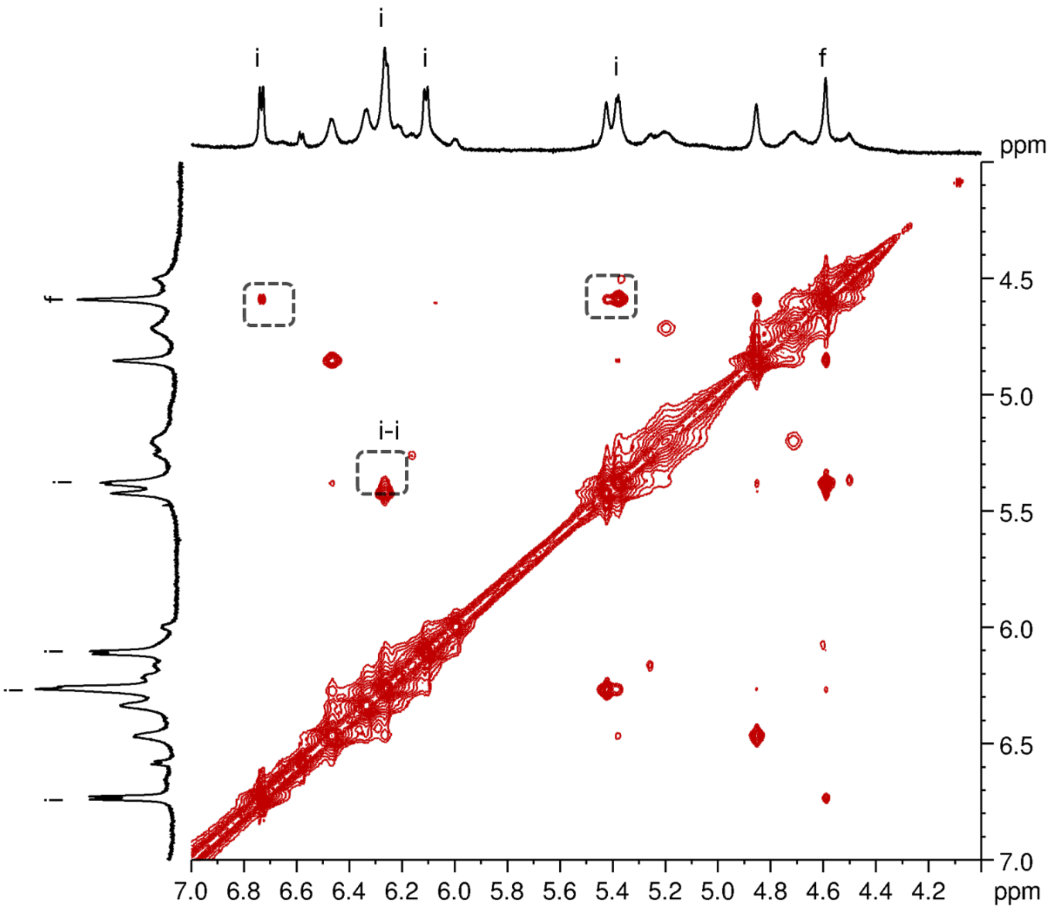

Supplementary Figure 62. Selected region of the ^{1}H-^{1}H NOESY spectrum (700 MHz, CD_3CN, 298K) of *trans*-perfluorodecalin⊂**1**. Three NOE cross peaks between the protons **f-i** and **i-i** have been identified, which indicates the rotation of the naphthalene units.

5.4 Ion mobility mass spectrometry (IMS) studies

Ion mobility measurements were performed on a Waters Synapt G2-Si HDMS mass spectrometer. All ions were generated by electrospray ionization (ESI) in the positive mode. Soft ionization conditions were used throughout the experiments: Capillary voltage 3 kV, sampling cone voltage 40 V, source offset 80 V, source temperature 80 °C, desolvation temperature 20 °C, cone gas flow rate 61 L/h, desolvation gas flow rate 600 L/h, nebulizer pressure 4.1 bar, ion mobility wave velocity 721 m/s, wave height 33.5 V. All other parameters were used as default. Collisional cross section (CCS) area was calculated using Waters Driftscope, calibrated relative to an aqueous solution of ubiquitin (1.0 mg·mL^{-1}). All datasets are recorded as one replicate with 300 scans. The source data of the spectra recorded are provided as the source data file of Figure 5.

Sample preparation

Samples of **1** and its corresponding host-guest complexes for ion mobility studies have been prepared and diluted to 1.0 mg·mL^{-1} in acetonitrile. Samples were then

injected into the ESI source with a syringe pump at an initial flow rate of 421 μL·h^{-1}.

Data processing

Data from ion mobility measurements in this study were processed with Waters Driftscope according to the following[19-21]:

- Flight time (t_C): corrected dt in ms at a determined wave height (i.e. 40V)

$$t_C = \frac{t_D - C \times m/z^{\frac{1}{2}}}{1000} \qquad \text{(S2)}$$

where t_D can be calculated from the drift-time function in Masslynx when displaying the chromatogram in time (i.e. 14 ms), not scans (200 bins) and C is the EDC coefficient of the instrument (i.e. $\frac{1.4}{1.8}$).

- Reduced mass (m_{red})

$$m_{red} = \frac{m_1 \times m_2}{m_1 + m_2} \qquad \text{(S3)}$$

where m_1 is the MW of the analyte of interest and m_2 is the mass of the mobility gas (28.01 for N_2).

- Calibration corrected CCS (Ω_C) in Å^2

$$\Omega_C = A \times t_c^{B} \qquad \text{(S4)}$$

where A and B values are from calibration chart [ln Ω_C = B × ln t_C + ln A for a linear relationship]

- Charge adjusted CCS (Ω_D) in Å^2

$$\Omega_D = \Omega_C \times \frac{z}{m_{red}^{\frac{1}{2}}} \qquad \text{(S5)}$$

Ion Mobilograms

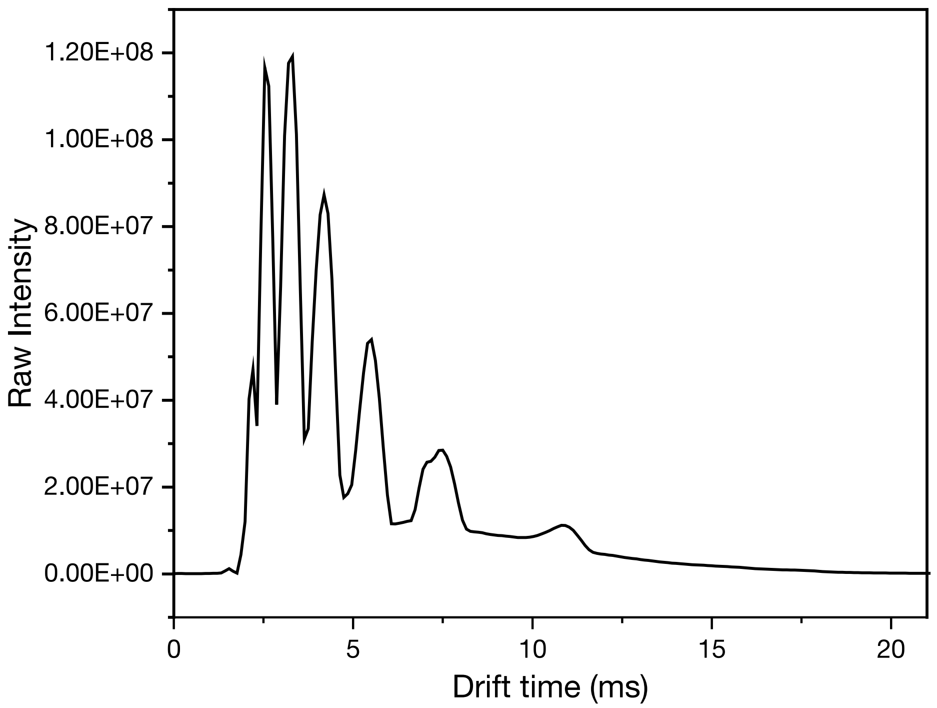

Supplementary Figure 63. Ion mobilogram of pseudo cube **1** (0.2 mM in acetonitrile).

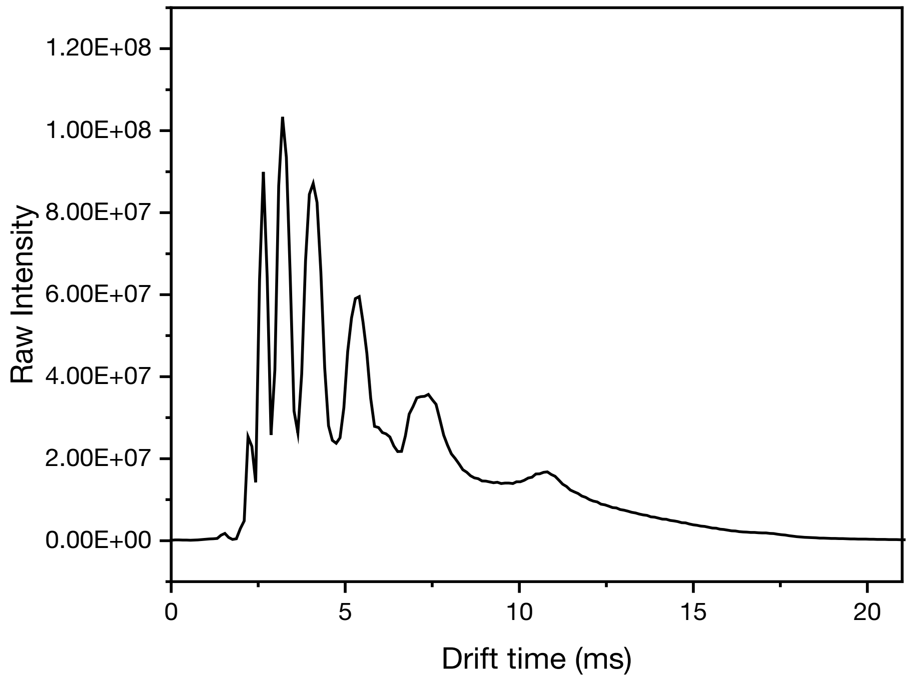

Supplementary Figure 64. Ion mobilogram of α-pinene⊂**1** (0.2 mM in acetonitrile).

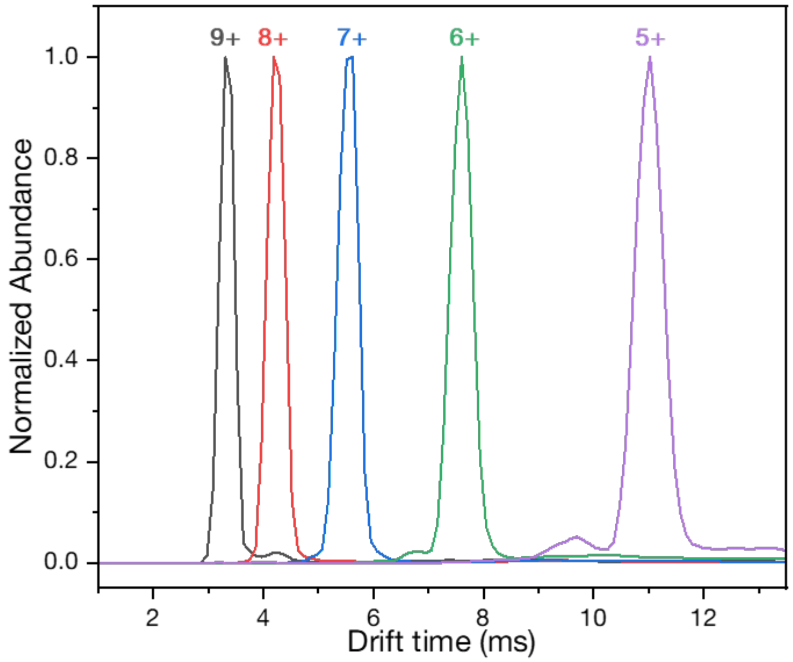

Supplementary Figure 65. Normalized ion peaks with different charges of α-pinene⊂**1**, as deconvolved from IMS data (0.2 mM in acetonitrile).

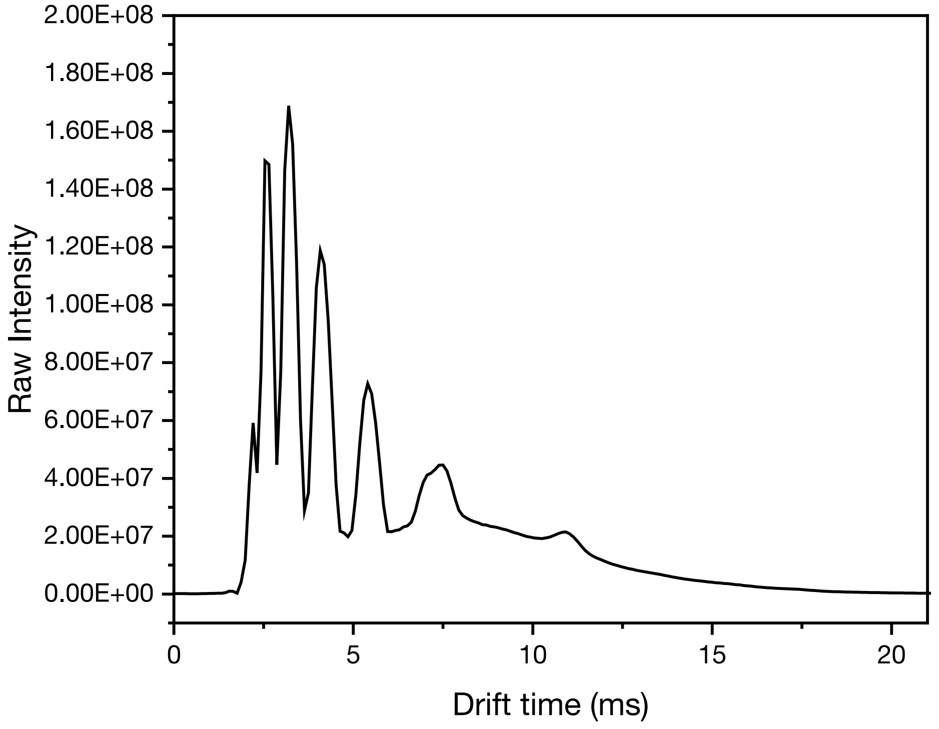

Supplementary Figure 66. Ion mobilogram of *trans*-decalin⊂**1** (0.2 mM in acetonitrile).

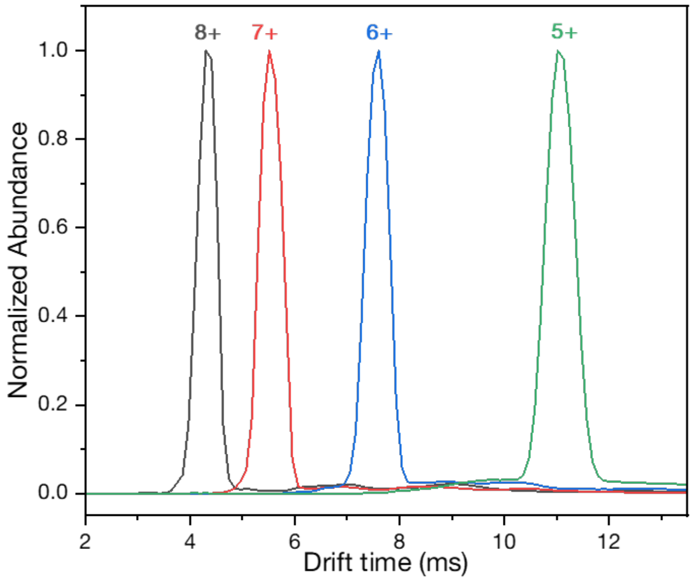

Supplementary Figure 67. Normalized ion peaks with different charges of *trans*-decalin⊂1, as deconvolved from IMS data (0.2 mM in acetonitrile).

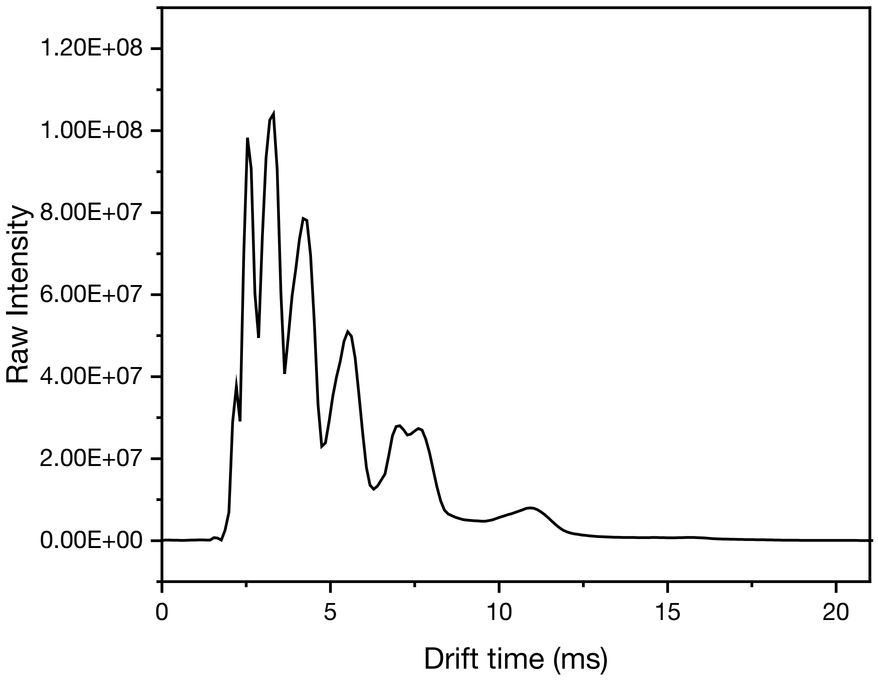

Supplementary Figure 68. Ion mobilogram of diamantane⊂**1** (0.2 mM in acetonitrile).

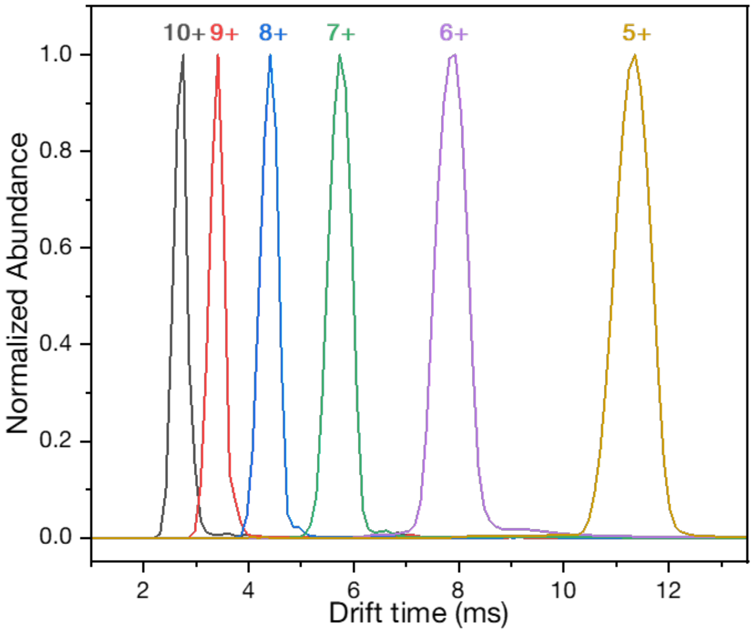

Supplementary Figure 69. Normalized ion peaks with different charges of diamantane⊂**1**, as deconvolved from IMS data (0.2 mM in acetonitrile).

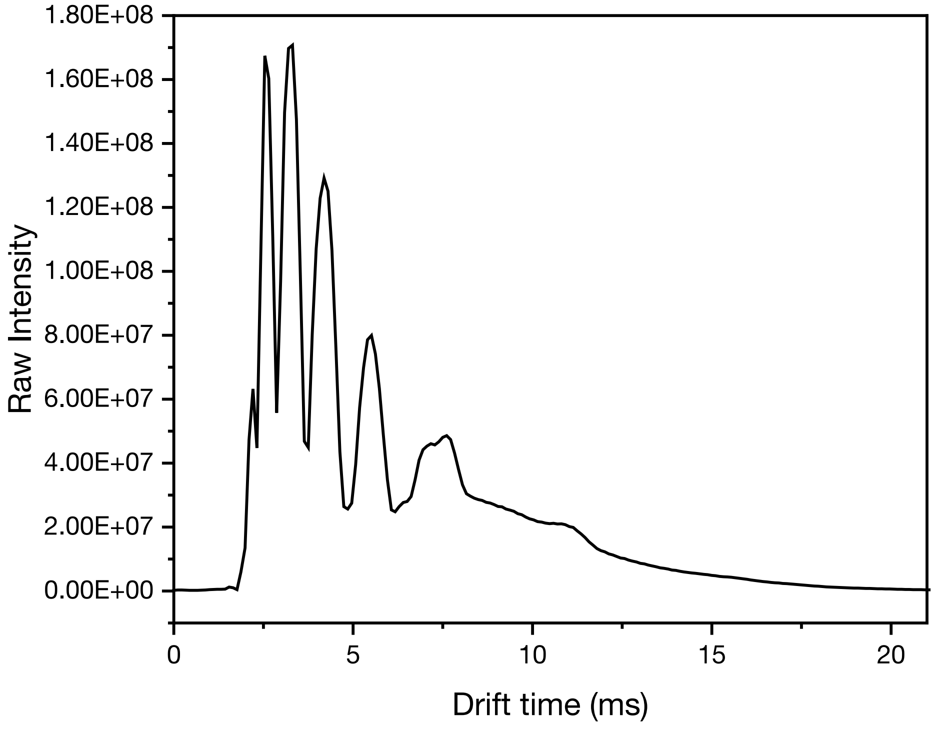

Supplementary Figure 70. Ion mobilogram of *trans*-perfluorodecalin⊂**1** (0.2 mM in acetonitrile).

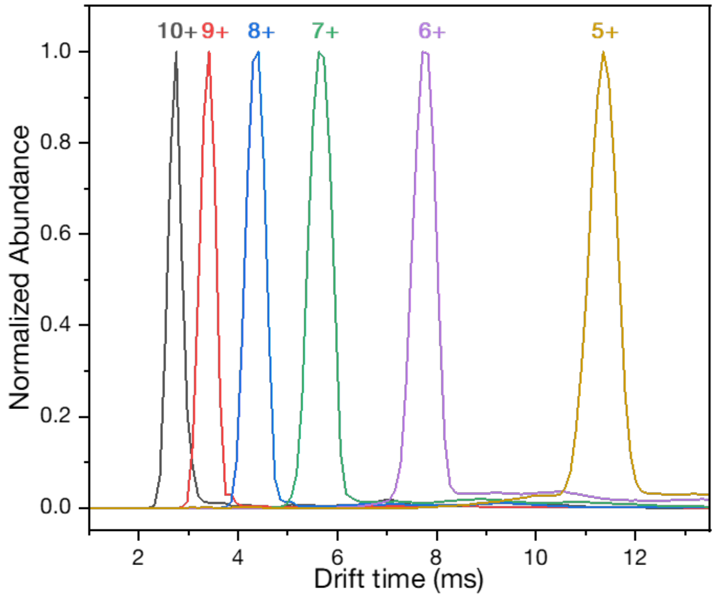

Supplementary Figure 71. Normalized ion peaks with different charges of *trans*-perfluorodecalin⊂**1**, as deconvolved from IMS data (0.2 mM in acetonitrile).

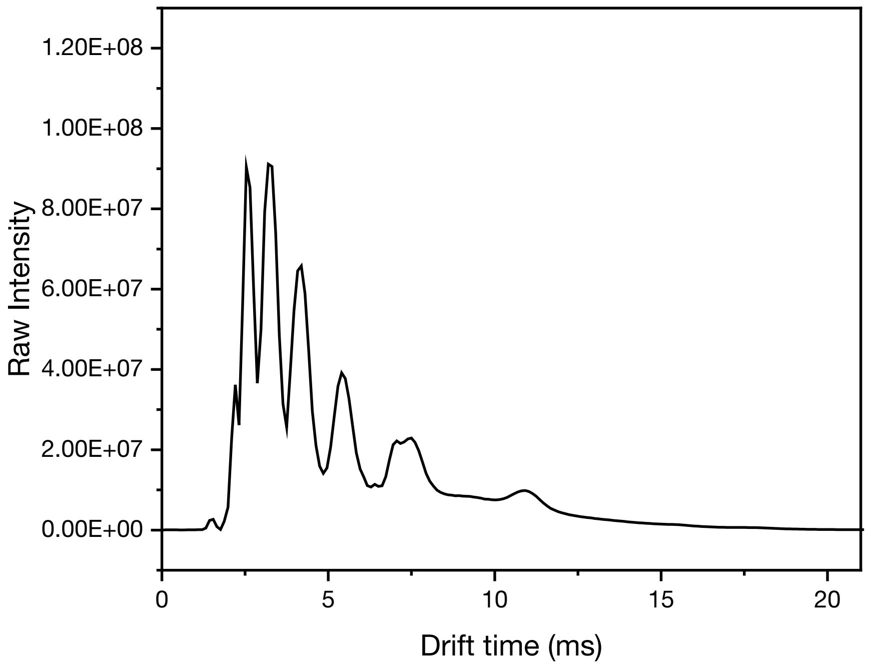

Supplementary Figure 72. Ion mobilogram of corannulene⊂**1** (0.2 mM in acetonitrile).

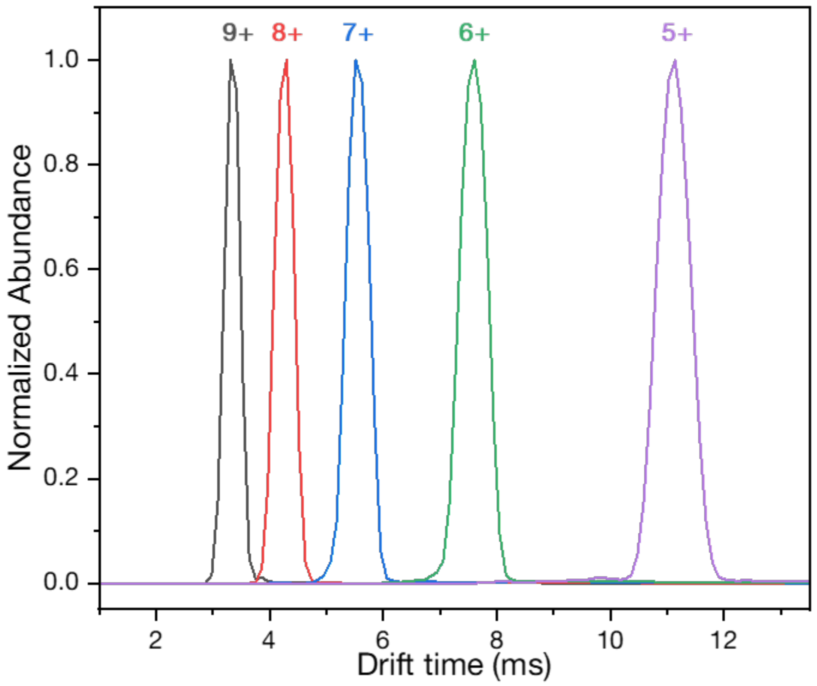

Supplementary Figure 73. Normalized ion peaks with different charges of corannulene⊂**1**, as deconvolved from IMS data (0.2 mM in acetonitrile).

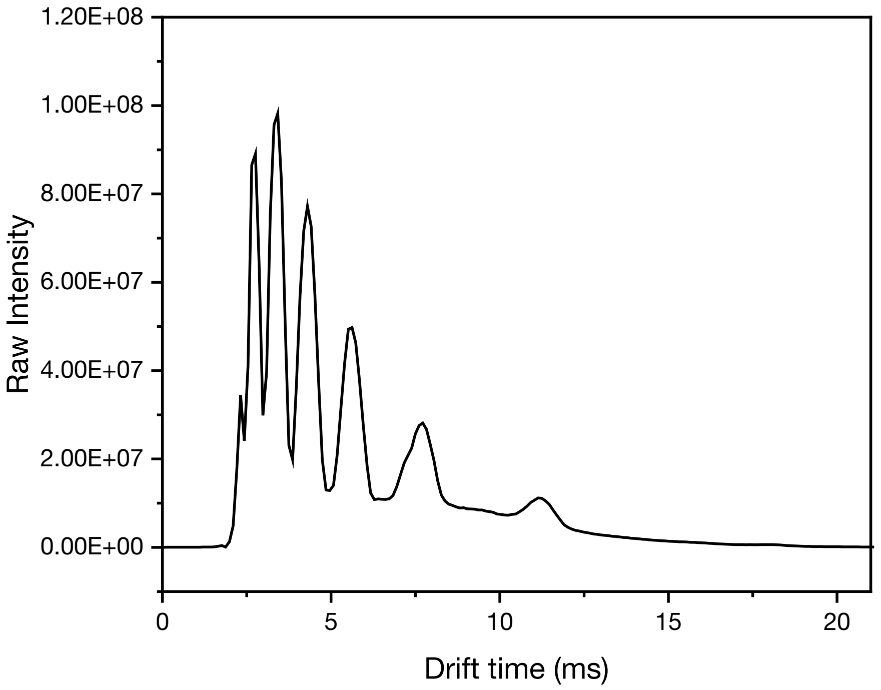

Supplementary Figure 74. Ion mobilogram of perhydropyrene⊂**1** (0.2 mM in acetonitrile).

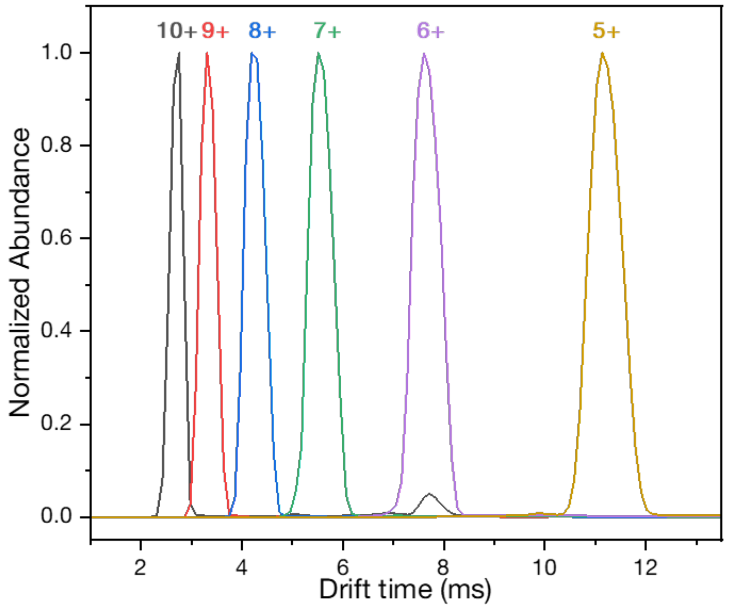

Supplementary Figure 75. Normalized ion peaks with different charges of perhydropyrene⊂**1**, as deconvolved from IMS data (0.2 mM in acetonitrile).

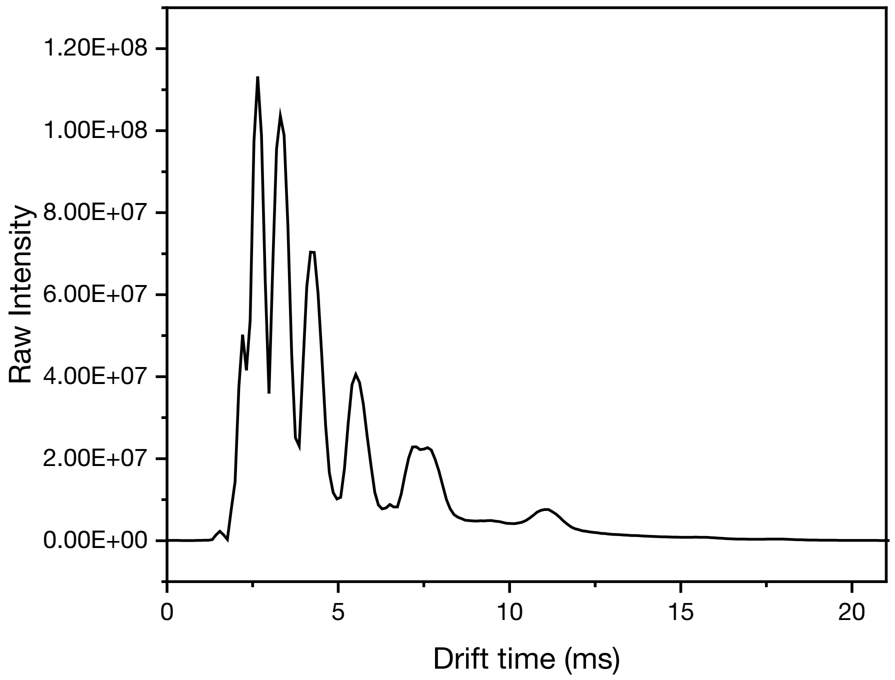

Supplementary Figure 76. Ion mobilogram of Aldrin⊂**1** (0.2 mM in acetonitrile).

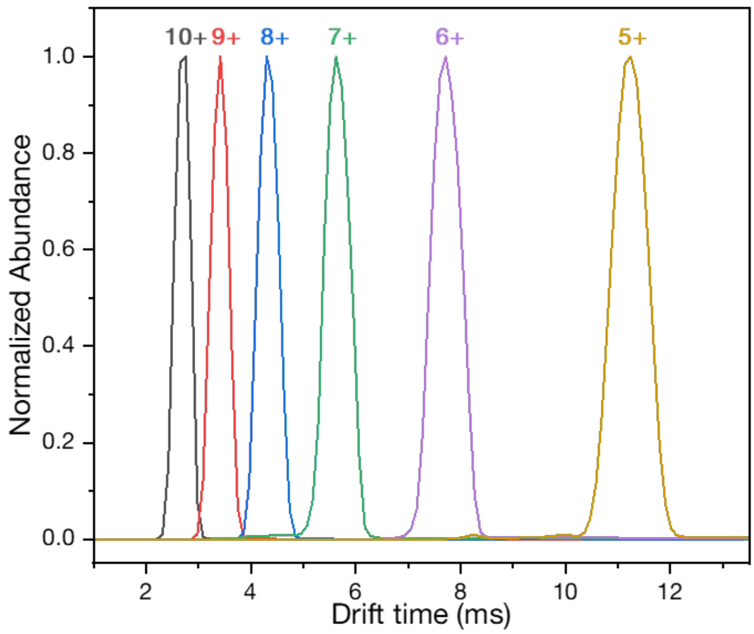

Supplementary Figure 77. Normalized ion peaks with different charges of Aldrin⊂**1**, as deconvolved from IMS data (0.2 mM in acetonitrile).

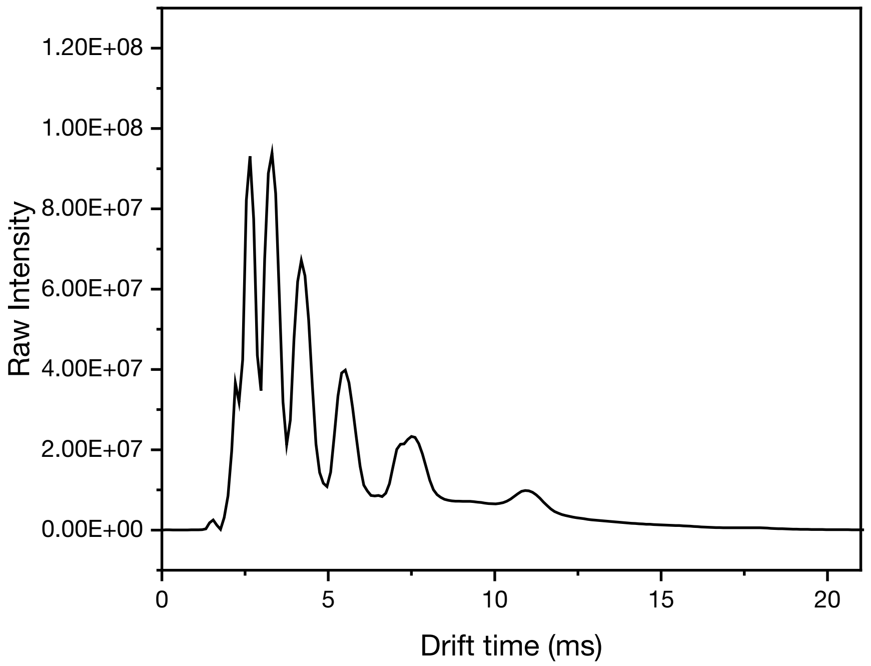

Supplementary Figure 78. Ion mobilogram of Endrin⊂**1** (0.2 mM in acetonitrile).

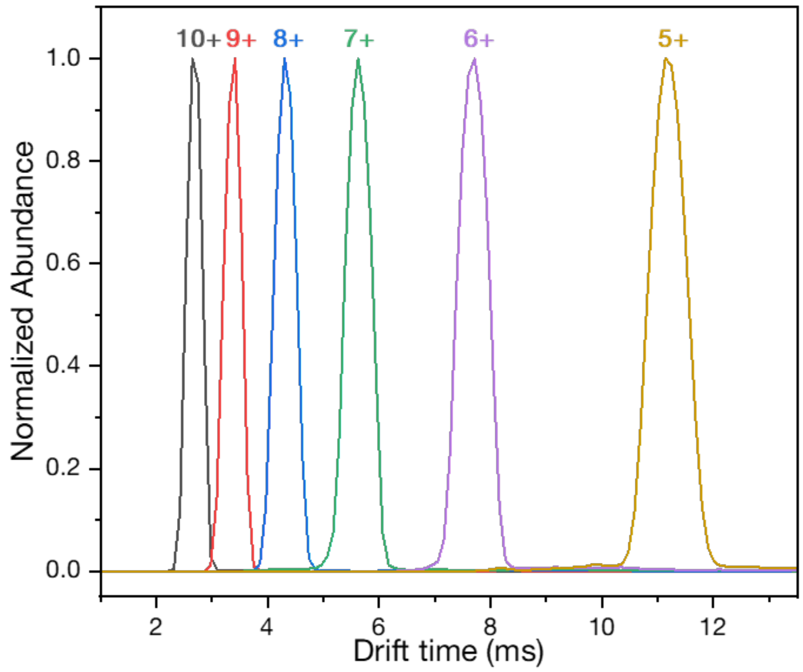

Supplementary Figure 79. Normalized ion peaks with different charges of Endrin⊂**1**, as deconvolved from IMS data (0.2 mM in acetonitrile).

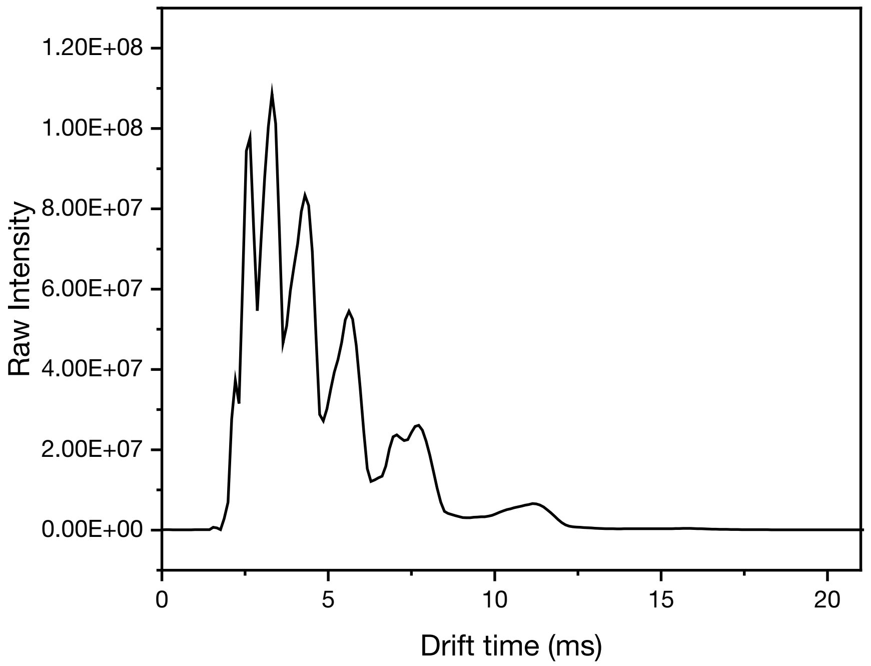

Supplementary Figure 80. Ion mobilogram of γ-chlordane⊂**1** (0.2 mM in acetonitrile).

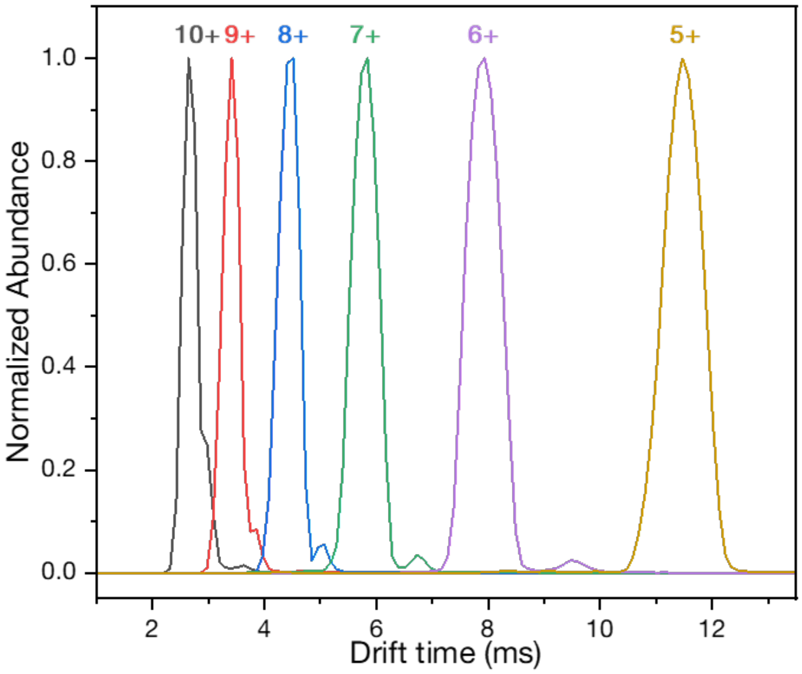

Supplementary Figure 81. Normalized ion peaks with different charges of the host-guest complex of pseudo cube **1** and γ-chlordane⊂**1**, as deconvolved from IMS data (0.2 mM in acetonitrile).

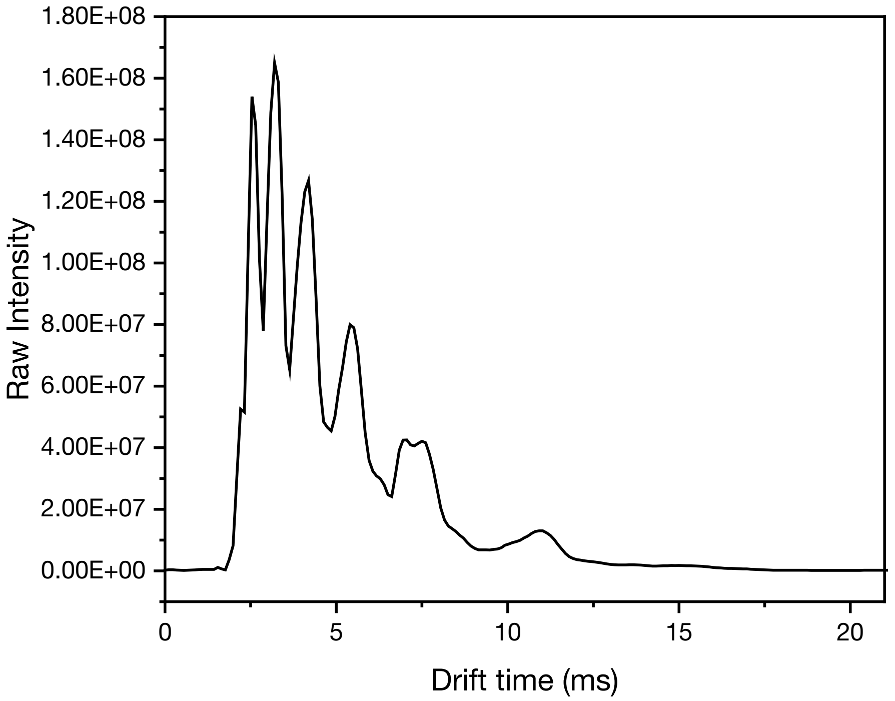

Supplementary Figure 82. Ion mobilogram of Kepone hydrate⊂**1** (0.2 mM in acetonitrile).

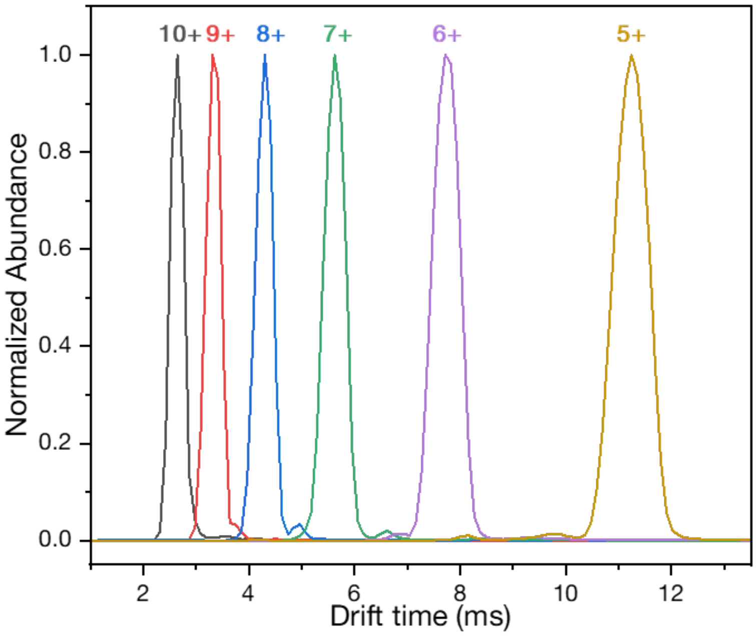

Supplementary Figure 83. Normalized ion peaks with different charges of Kepone hydrate⊂**1**, as deconvolved from IMS data (0.2 mM in acetonitrile).

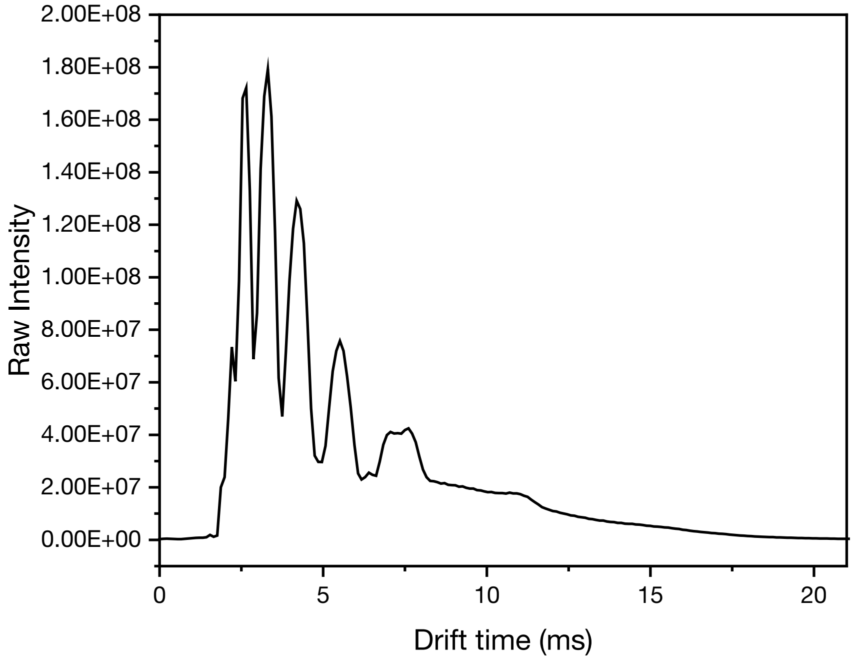

Supplementary Figure 84. Ion mobilogram of Mirex⊂**1** (0.2 mM in acetonitrile).

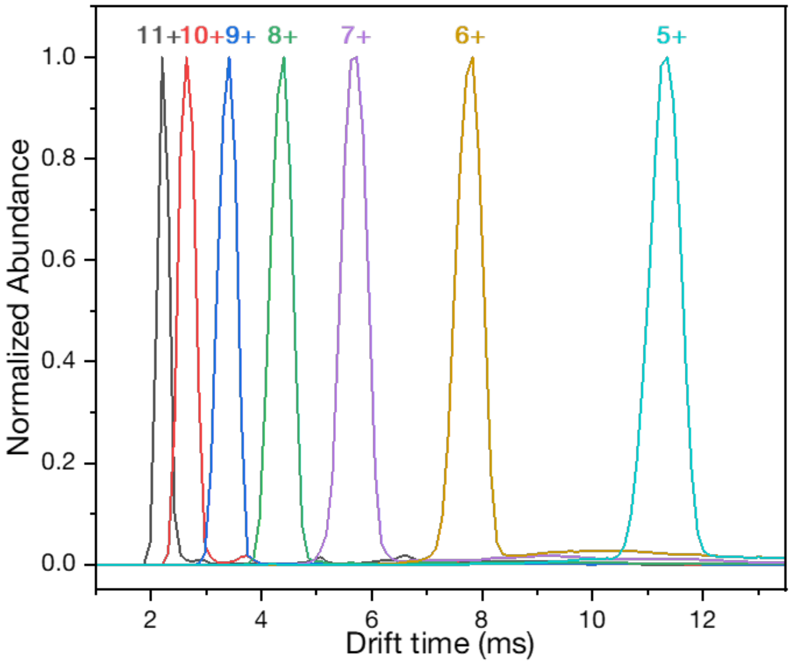

Supplementary Figure 85. Normalized ion peaks with different charges of Mirex⊂**1**, as deconvolved from IMS data (0.2 mM in acetonitrile).

Supplementary Table 3. Calculated average CCS values of distinct ion peaks from 1 and corresponding host-guest complexes (Å^2). Data are presented as mean values ± standard deviations from the CCS values of ~1000-3000 distinct ion peaks.

Peak charge \ guest name	11+	10+	9+	8+	7+	6+	5+
Empty cage 1	1034 ± 4	1059 ± 3	1115 ± 4	1166 ± 4	1212 ± 4	1267 ±3	1346 ± 2
α-Pinene			1116 ± 3	1157 ± 3	1204 ± 2	1266 ±2	1342 ± 2
trans-Decalin				1172 ± 3	1206 ± 2	1266 ± 2	1349 ± 2
Diamantane	1037 ± 3	1078 ± 4	1130 ± 6	1187 ± 4	1233 ± 3	1295 ± 2	1366 ± 2
trans-Per-fluorodecalin		1085 ± 3	1129 ± 4	1179 ± 4	1221 ± 3	1284 ± 2	1369 ± 2
Corannulene			1117 ± 3	1162 ± 3	1207 ± 3	1268 ± 2	1352 ± 2
Perhydropy-rene		1081 ± 2	1116 ± 3	1163 ± 3	1210 ± 2	1275 ± 2	1359 ± 1
Aldrin		1080 ± 3	1131 ± 5	1176 ± 4	1219 ± 4	1281 ± 3	1359± 2
Endrin		1078 ± 3	1127 ± 7	1173 ± 4	1218 ± 3	1279 ± 2	1358 ± 2
γ-Chlordane		1078 ± 16	1131 ± 4	1193 ± 3	1242 ± 3	1303 ± 3	1379 ± 2
Chlordecone	1050 ± 5	1067 ± 7	1116 ± 6	1168 ± 5	1216 ± 4	1283 ± 3	1360 ± 3
Mirex	1048 ± 3	1070 ± 6	1130 ± 5	1182 ± 4	1226 ± 4	1287 ± 4	1368 ± 4

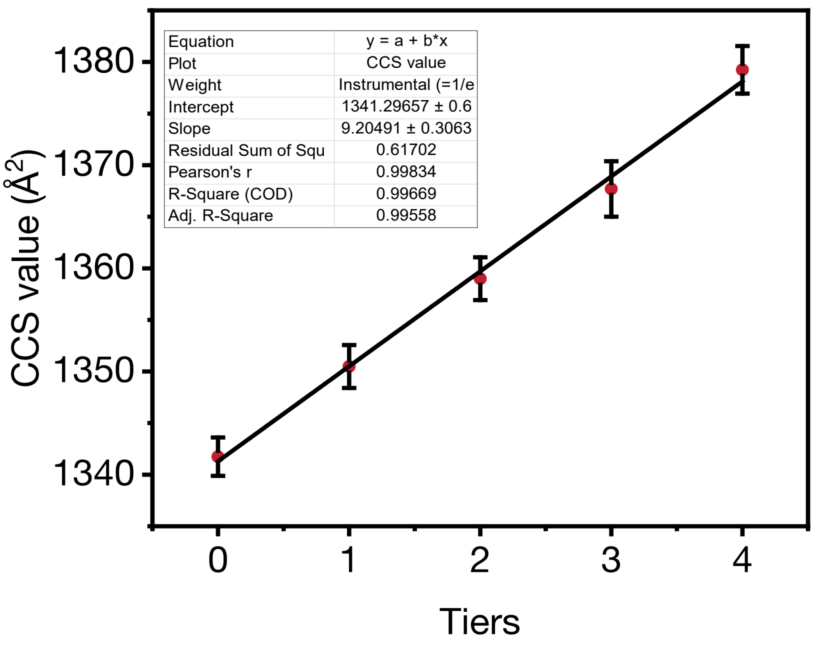

Supplementary Figure 86. Linear fitting of the CCS values in Fig. 5c to determine the quantum of cavity size increase corresponding to one tier. Data are presented as mean values ± standard deviation calculated from data in Fig. 5c.

Here the CCS values were fitted to the following equation:

$$CCS_{guest\subset 1} = 1341.3\ Å^2 + (n)\cdot 9.2\ Å^2 \qquad (S7)$$

$$CCS_{guest\subset 1} = CCS^0_{guest\subset 1} + n\cdot \Delta CCS \qquad (S8)$$

Linear least-squares fitting provided a tier increment value of 9.2 Å^2 and a smallest CCS guest⊂**1** value of 1341 Å^2. The R^2 value was 0.997, suggesting a good linear correlation, with the states of the cage faces well-described by quanta of volume corresponding to the tiers. The intercept, indicating the value of CCS value as the first tier or smallest CCS value of guest⊂**1**, was fitted as 1341.3 Å^2, while the increment of CCS value per tier was determined as 9.2 Å^2.

5.5 DOSY spectra

Data were processed using Bruker Dynamics Center 2.8.0.1 and diffusion coefficients are given as the average value of the cage signals from the list provided by the report generated. Errors of diffusion coefficients are reported as calculated in Bruker Dynamics Center 2.8.0.1 from Monte Carlo simulations. Errors of the calculated solvodynamic diameters reported as the standard deviations of the values calculated from the individual cage signals in the list, and are summarized in Supplementary Table 4. The detailed solvodynamic diameters of individual peaks are also provided in

the form of the source data for Figure 5. All datasets are recorded as one replicate with 48 scans per increment.

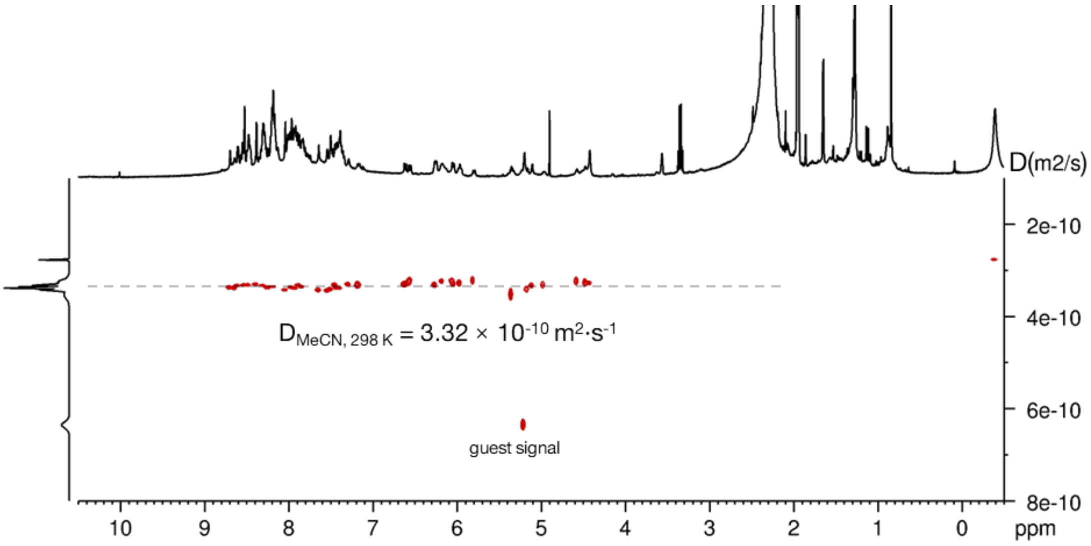

Supplementary Figure 87. ^{1}H-DOSY spectrum (400 MHz, CD_3CN, 298K) of *α*-pinene⊂**1** (with ca. 15 equiv. of *α*-pinene).

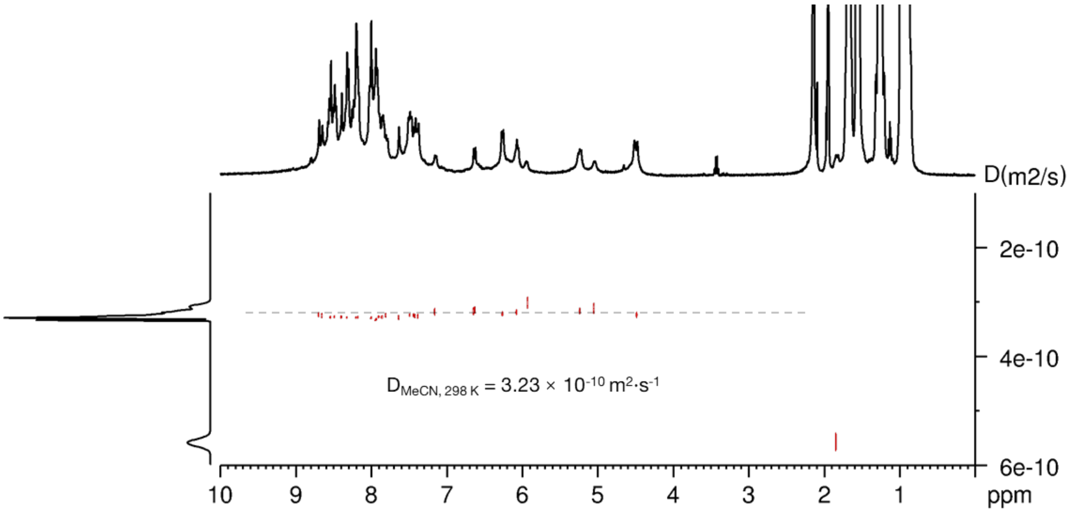

Supplementary Figure 88. ^{1}H-DOSY spectrum (400 MHz, CD_3CN, 298K) of *trans*-decalin⊂**1** (with ca. 15 equiv. of *trans*-decalin).

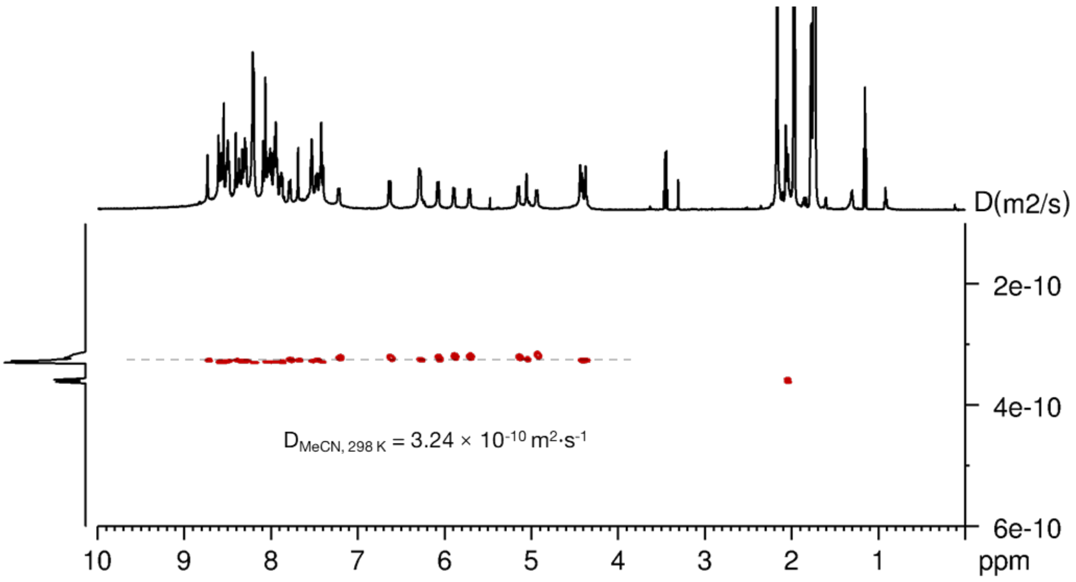

Supplementary Figure 89. ^{1}H-DOSY spectrum (400 MHz, CD_3CN, 298K) of diamantane⊂**1** (with ca. 10 equiv. of diamantane).

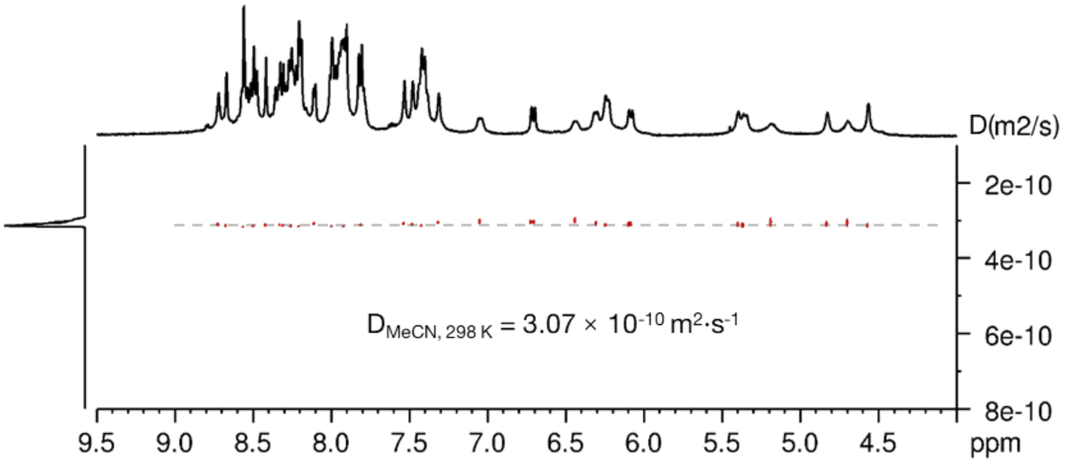

Supplementary Figure 90. ^{1}H-DOSY spectrum (400 MHz, CD_3CN, 298K) of *trans*-perfluorodecalin⊂**1** (prepared with ca. 20 equiv. of *trans*-perfluorodecalin, precipitated by Et_2O and redissolved).

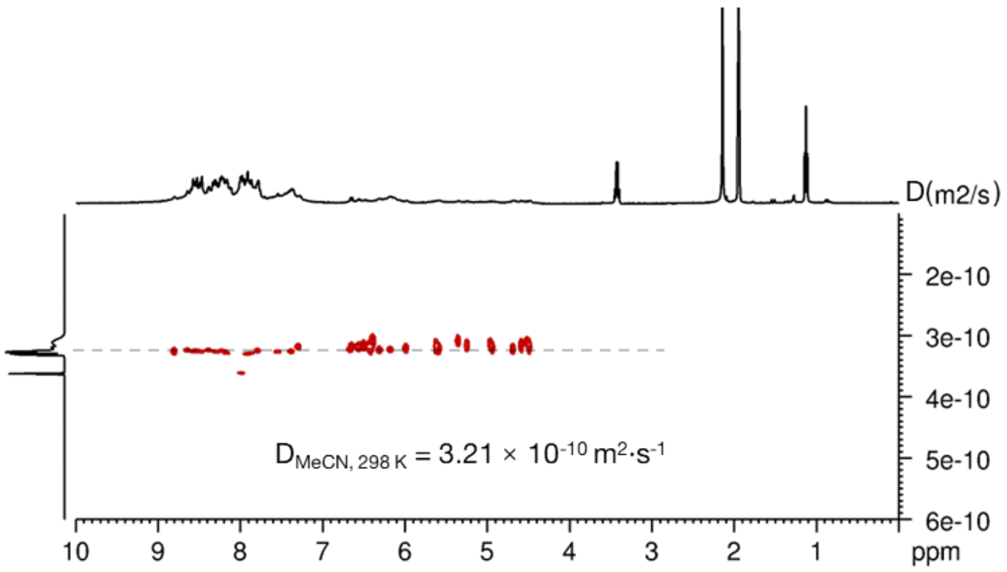

Supplementary Figure 91. ^{1}H-DOSY spectrum (400 MHz, CD_3CN, 298K) of corannulene⊂**1** (prepared with ca. 20 equiv. of corannulene, after Et_2O washing purification).

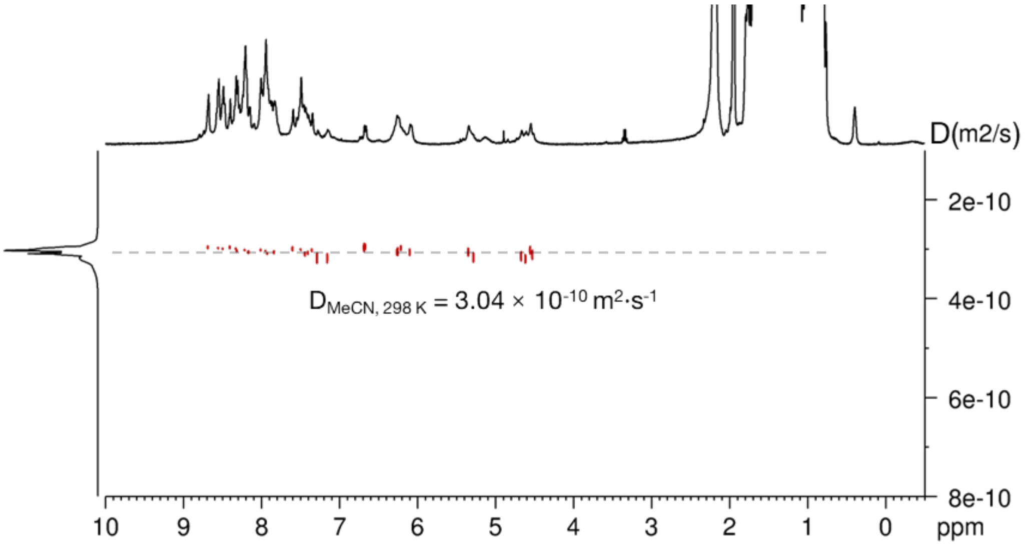

Supplementary Figure 92. ^{1}H-DOSY spectrum (400 MHz, CD_3CN, 298K) of perhydropyrene⊂**1** (prepared with ca. 40 equiv. of perhydropyrene).

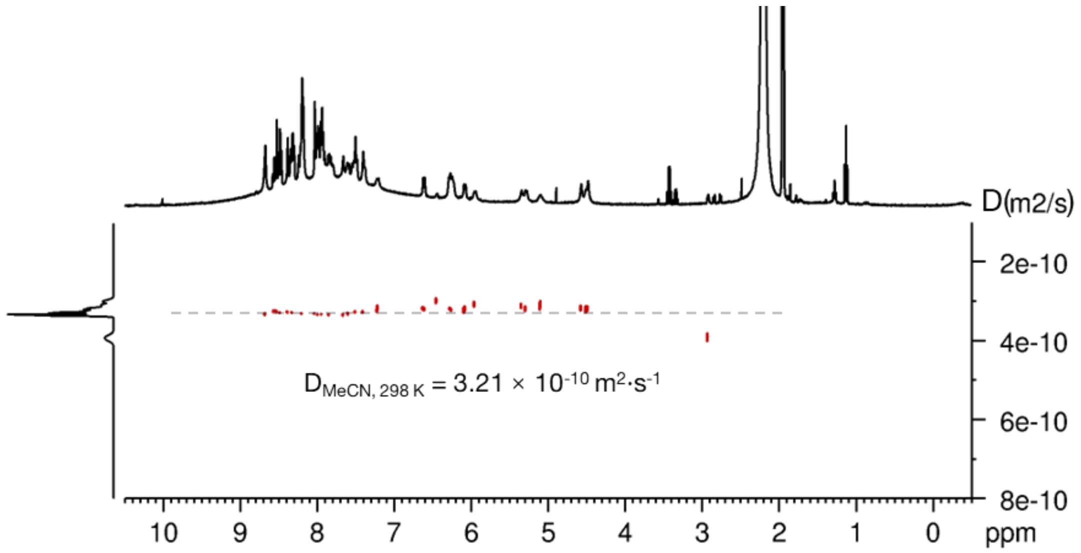

Supplementary Figure 93. ^{1}H-DOSY spectrum (400 MHz, CD_3CN, 298K) of Aldrin⊂**1** (prepared with ca. 10 equiv. of Aldrin after Et_2O washing purification).

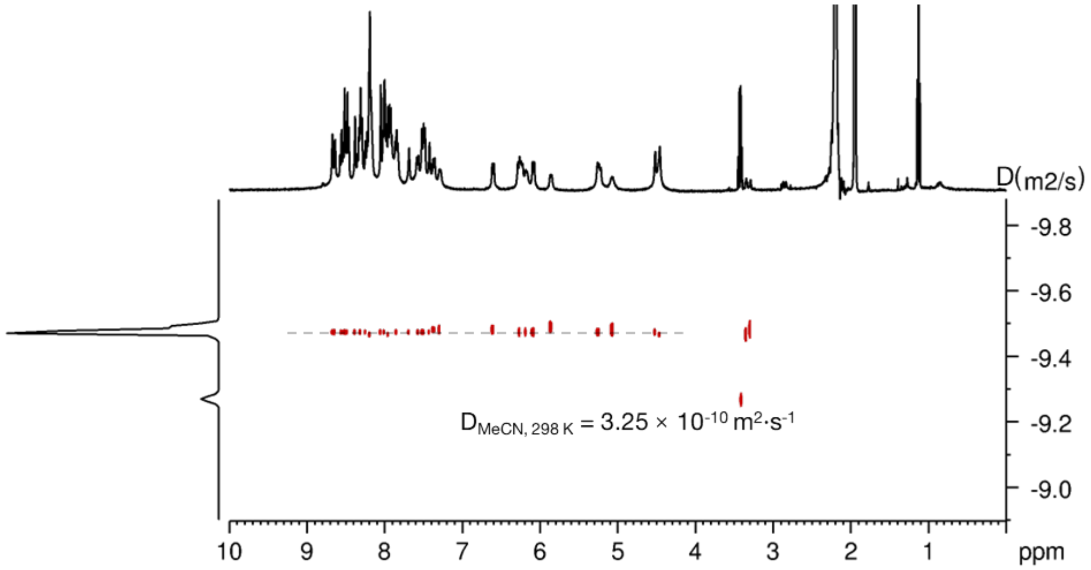

Supplementary Figure 94. ^{1}H-DOSY spectrum (400 MHz, CD_3CN, 298K) of Endrin⊂**1**(prepared with ca. 10 equiv. of Endrin after purification by washing with Et_2O).

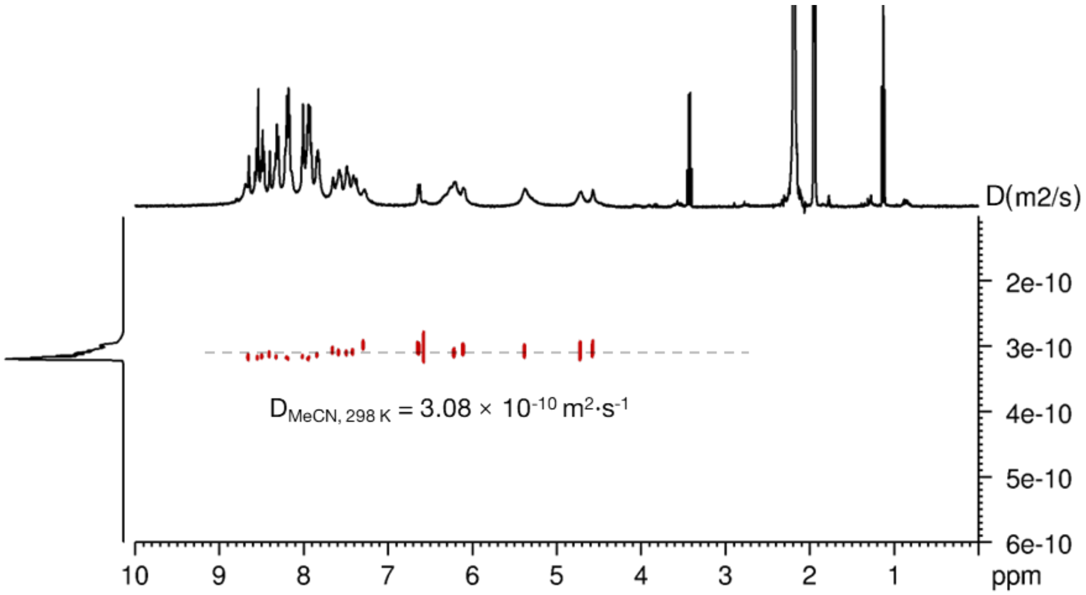

Supplementary Figure 95. ^{1}H-DOSY spectrum (400 MHz, CD_3CN, 298K) of γ-Chlordane⊂**1** (prepared with ca. 10 equiv. of γ-Chlordane after purification by washing with Et_2O).

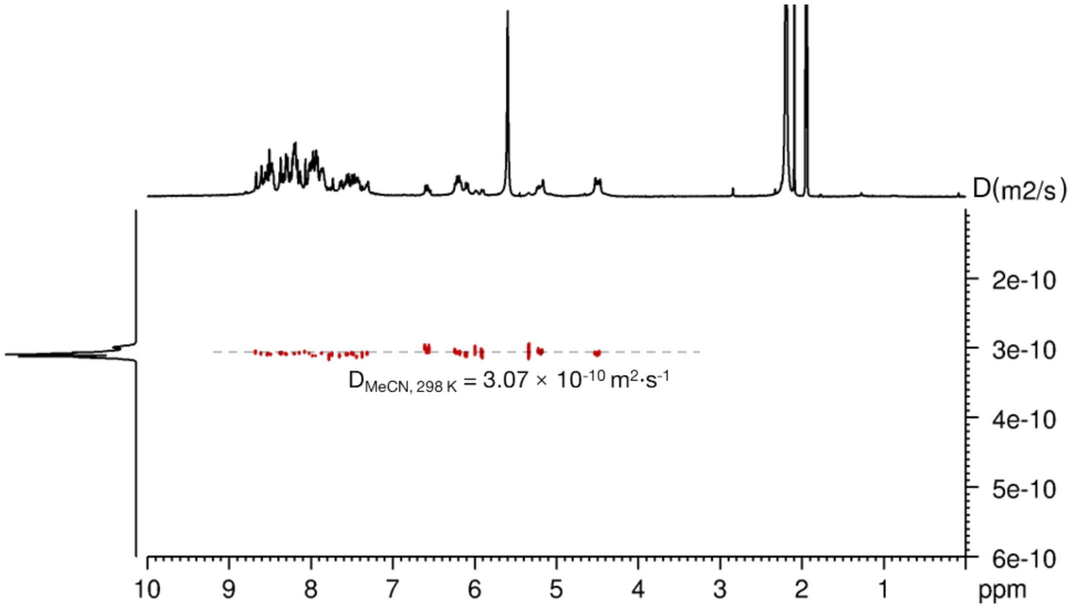

Supplementary Figure 96. ^{1}H-DOSY spectrum (400 MHz, CD_3CN, 298K) of Kepone hydrate⊂**1** (with ca. 20 equiv. of Kepone (a.k.a. Chlordecone)). Under the experimental conditions Kepone hydrates and the hydrated product is bound by the cage, as revealed by mass spectrometry, no evidence was observed of Kepone bound by the cage.

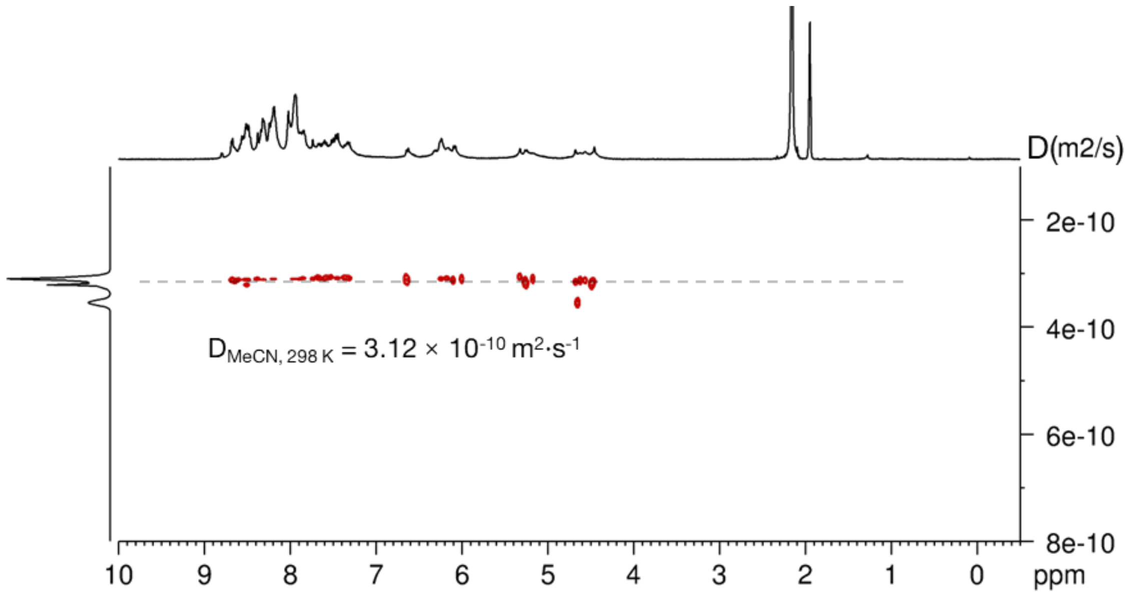

Supplementary Figure 97. ^{1}H-DOSY spectrum (400 MHz, CD_3CN, 298K) of Mirex⊂**1** (prepared with ca. 20 equiv. of Mirex).

Supplementary Table 4. Diffusion coefficients calculated solvodynamic diameters of cage **1** and corresponding host-guest complexes

	Diffusion coefficient (10^{-10} $m^2 \cdot s^{-1}$)	Standard Deviation of diffusion coefficient (10^{-10} $m^2 \cdot s^{-1}$)	Solvodynamic diameter (Å)	Standard Deviation of solvodynamic diameter (Å)
Empty cage 1	3.34	0.03	38.4	0.4
α-Pinene	3.32	0.07	38.6	0.8
trans-Decalin	3.23	0.05	39.6	0.6
Diamantane	3.24	0.03	39.6	0.4
trans-Perfluorodecalin	3.07	0.04	41.8	0.6
Corannulene	0.00	0.00	40.0	0.7
Perhydropyrene	3.04	0.07	42.2	1.0
Aldrin	3.21	0.09	39.9	1.1
Endrin	3.25	0.04	39.4	0.5
γ-Chlordane	3.08	0.08	41.6	1.2
Kepone hydrate	3.07	0.03	41.7	0.4
Mirex	3.12	0.10	41.1	1.2

6 Computational studies

6.1 Structure modelling of B(p-Cl-C_6H_4)$^-$⊂1

Semi-empirical quantum mechanical calculations of the guest encapsulated MOC were carried out in ORCA (version 5.0.4 with OpenMPI 4.1.1) through an interface to the xtb package (version 6.1.1)[22, 23]. The xtb package includes the semiempirical quantum mechanical (SQM) methods GFN*n*-xTB. These methods were parameterized for elements up to Z=86 and were shown to give reasonably reliable results for the optimization of large transition metal-containing structures[24].

The structure was initially modelled using the MM3 force field in Scigress[25-29]. The resulting structure was used as an input for a geometry optimization performed at the GFN2-xTB level of theory[29]. A charge of 15 was assigned to the structure. A well converged minimum was obtained for the gas phase. A vibrational analysis of the optimized geometry indicated that no negative frequencies were observed and thus a correct minimum was obtained.

The atomic coordinates of this structure are provided in Supplementary Data 3.

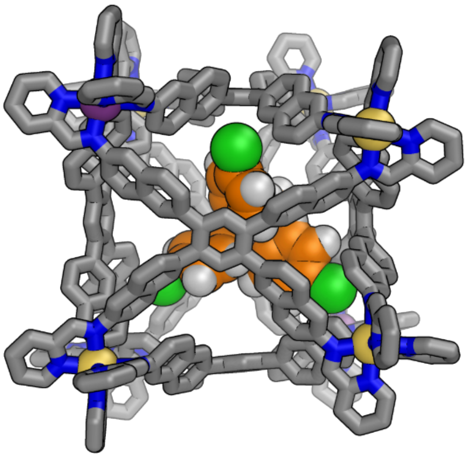

Supplementary Figure 98. GFN2-xTB model of B(p-Cl-C_6H_4)$^-$⊂1[29], suggesting *exo* facial conformations. Color codes: C = grey or orange (guest), N = blue, *fac*-Δ-Zn = yellow orange, *fac*-Λ-Zn = purple, Cl = green, H = white. Hydrogens of the cage have been omitted for clarity.

6.2 Structural transformation simulations

Pathway calculations for the structural transformation from the all-*endo* to the all-*exo* pseudocube were performed at the GFN-FF[30] level of theory, using the PATHSAMPLE[31] program to organize parallel OPTIM jobs[32]. GFN-FF is a semi-polarizable force-field related to the GFN*n*-xTB methods in purpose and design.[23] Like the xTB methods it is parametrized for all elements up to radon and was chosen for the pathway calculations due to the high computational cost, which would make the calculation unfeasible, even at a SQM level of theory. Energies for the local minima

and transition states in the resulting 40-step pathway are shown relative to the *endo* starting point in Supplementary Table 5 and illustrated in Supplementary Fig. 101, respectively. The atomic coordinates of every step of this pathway are provided in Supplementary Data 4. A disconnectivity graph[33,34] corresponding to the system and the pathway is shown in Figure S118, where minima corresponding to the *endo* and *exo* endpoint have been marked accordingly.

The minima and transition states along the pathway were taken as input for further single point energy evaluations, as discussed in the manuscript. At the density functional theory (DFT) level, single point evaluations were performed at the r^2SCAN-3c level[35], using the ORCA program. This DFT functional of the meta-GGA type has previously performed very well for molecular and periodic systems and was chosen for our evaluations due to a balanced cost-to-accuracy ratio. Nonetheless, we note that each DFT single point evaluation, with a computational wall-time of roughly 6 hours (using 6 cores of a 2.50 GHz Intel Xeon Gold 6248 CPU), is several orders of magnitude more expensive than GFN2-xTB or GFN-FF based calculations. Finally, to estimate the influence of solvation, single point calculations along the pathway were performed with CREST (version 3.0)[36] at the GFN-FF level including ALPB implicit solvation[37], parametrized for acetonitrile. Relative energies for r^2SCAN-3c and GFN-FF/ALPB (acetonitrile) are included in Supplementary Table 5.

Supplementary Table 5. The relative energies (in kJ/mol) of the structures in the transformation pathway of **1** from fully *endo* to fully *exo*.

Step	GFN-FF	r^2SCAN-3c	GFN-FF/ALPB(acetonitrile)
0	0.0	0.0	0.0
1	1.5	15.0	-0.4
2	-2.7	-14.9	5.6
3	-0.4	2.6	4.9
4	-9.0	-37.8	18.8
5	21.3	21.1	34.8
6	14.0	24.6	41.8
7	18.4	33.3	46.3
8	-5.8	-33.9	28.7
9	7.8	6.4	42.0
10	-19.3	6.3	20.5
11	14.6	49.7	50.1
12	9.1	47.9	55.0
13	34.4	99.1	77.0
14	8.7	36.0	56.5
15	16.2	66.0	75.9
16	15.5	68.8	77.3
17	15.8	69.4	79.3
18	14.2	63.1	79.1
19	24.6	84.9	93.5
20	8.0	68.8	74.0
21	9.9	86.8	84.6
22	0.8	75.5	73.4
23	8.5	109.4	76.7
24	-13.5	92.7	52.7
25	11.9	136.2	74.2
26	1.0	148.8	76.5
27	2.0	159.8	81.8
28	-14.2	147.5	66.4
29	-9.5	141.8	74.7
30	-27.1	90.4	47.9
31	-24.5	105.8	49.9
32	-37.0	92.8	40.1
33	-36.5	97.9	44.6
34	-38.0	98.0	47.4

35	-20.3	117.2	68.9
36	-60.1	97.6	35.1
37	-55.3	113.0	44.9
38	-62.8	94.6	40.4
39	-59.9	108.8	47.3
40	-81.3	82.1	22.6
41	-52.0	128.0	58.7
42	-94.1	106.6	18.1
43	-71.4	136.0	37.5
44	-73.9	147.7	40.7
45	-70.3	153.8	46.3
46	-89.4	112.9	15.9
47	-80.3	138.1	34.3
48	-90.2	110.0	29.2
49	-86.3	130.4	35.0
50	-113.8	102.5	17.4
51	-88.1	115.1	30.4
52	-88.2	119.2	32.4
53	-83.1	129.0	40.5
54	-105.6	96.7	6.6
55	-103.7	115.6	10.3
56	-119.7	105.0	-8.3
57	-106.8	139.1	16.0
58	-137.9	68.6	-17.6
59	-113.5	113.0	8.1
60	-123.4	92.8	-13.8
61	-120.7	91.4	-10.6
62	-125.3	65.1	-15.8
63	-100.9	79.7	10.9
64	-124.6	84.3	-3.5
65	-112.5	117.8	20.8
66	-135.9	85.7	5.1
67	-129.2	113.6	14.6
68	-130.5	107.9	8.7
69	-117.2	121.5	22.5
70	-118.2	133.2	24.4
71	-117.1	144.1	29.7
72	-130.4	106.9	12.6
73	-127.4	97.2	13.2
74	-134.5	93.4	7.1

75	-101.3	144.7	33.6
76	-109.2	125.7	25.2
77	-89.0	132.1	34.1
78	-103.3	95.3	20.1
79	-100.5	123.3	25.7
80	-106.4	129.2	10.5

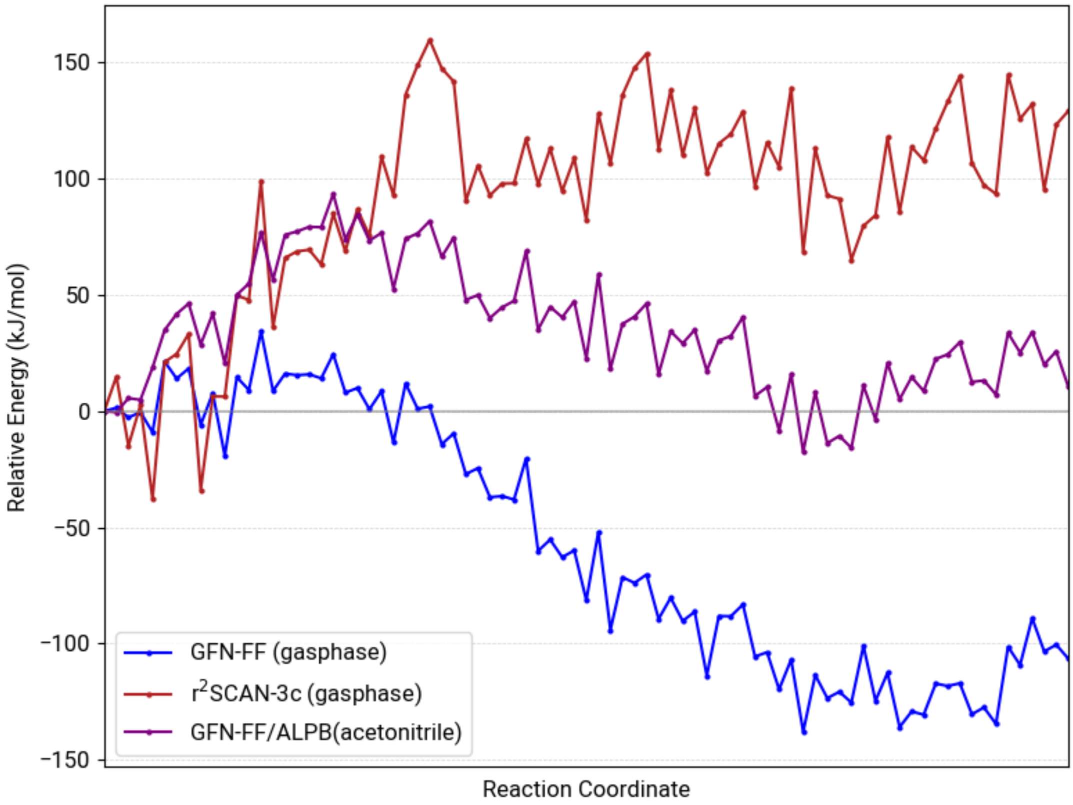

Supplementary Figure 99. The relative energy curves for the transformation of **1** from fully *endo* to fully *exo*, calculated at different levels.

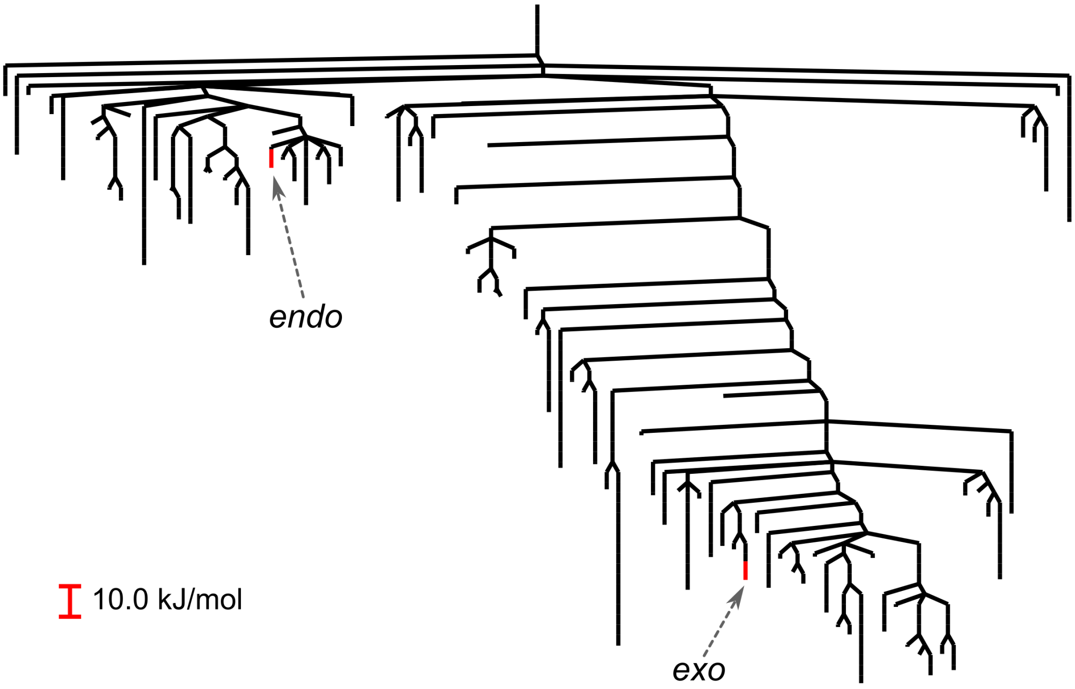

Supplementary Figure 100. Disconnectivity graph corresponding to the GFN-FF pathway shown in Figure S99.

6.3 Comparison of the energy barrier for the structural conversion of 1

The results above suggest that the structural exchange of **1** involves a forty distinct transition states, in which the highest energy barrier is 33.9-58.9 kJ·mol^{-1}, according to calculations at different levels. In order to compare this energy barrier with the fast-exchange NMR observed for the host-guest complexes of **1** at room temperature, variable temperature (VT) NMR experiments have been run to estimate the energy barrier for the structural transformation of **1** upon encapsulation of certain guest molecules.

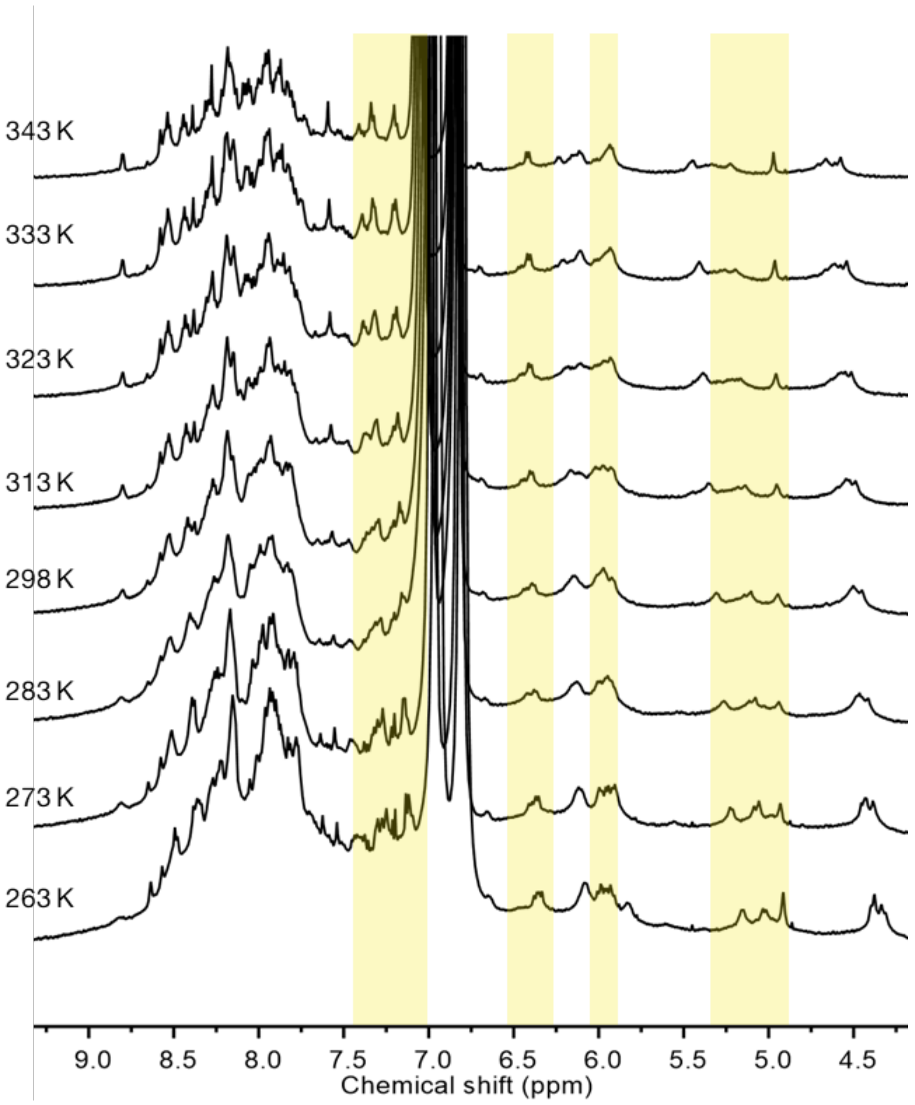

Supplementary Figure 101. Aromatic region of the variable-temperature NMR of B(*p*-Cl-C_6H_4)$^-$⊂**1** ([**1**] = 1.00 mM, [KB(*p*-Cl-C_6H_4)] = 10.0 mM).

It is evident from Supplementary Figure 101 that, the NMR signals of certain zones of B(*p*-Cl-C_6H_4)$^-$⊂**1** split to some degree under decreased temperature. This result suggests that the degree of dynamics of the naphthalene rotational units are more restricted at lower temperatures. The splitting of the peak at 5.12 ppm at 298 K has been taken as a representative signal for analysis. The data had been processed with Global Spectral Deconvolution (GSD) of MestreNova 12.0.0 and the FWHMs were read directly from MestreNova. The exchange rate constants were calculated with formulas below:

Intermediate temperatures:

$$k = \frac{\pi}{\sqrt{2}}[(\Delta v_A)^{e}_{\frac{1}{2}} - (\Delta v_A)^{o}_{\frac{1}{2}}] \quad \text{(S9)}$$

Coalescence:

$$k = \frac{\pi \Delta v_o}{\sqrt{2}} \qquad \text{(S10)}$$

Fast exchange temperatures:

$$k = \frac{\pi {\Delta v_o}^2}{2} \frac{1}{[(\Delta v_A)^e_{\frac{1}{2}} - (\Delta v_A)^o_{\frac{1}{2}}]} \qquad \text{(S11)}$$

$(\Delta v_A)^e_{\frac{1}{2}}$ is the FWHM of the chemical signal (or one of the chemical signals) in the given scenario, $(\Delta v_A)^o_{\frac{1}{2}}$ is the FWHM of one of the chemical signals at the slow exchange temperature, Δv_o is the FWHM of the signal at coalescence.

Chemical exchange rate constants for the estimation of the energy barrier for the structural transformation of B(*p*-Cl-$C_6H_4)^-$⊂**1** have been summarized in Supplementary Table 6.

Supplementary Table 6. Chemical exchange rate constants for the estimation of the energy barrier for the structural transformation of B(*p*-Cl-$C_6H_4)^-$⊂**1.**

T/K	T^{-1}/K^{-1}	k	lnk
343	0.002915	210.42695	5.34914
333	0.003003	31.81476	3.45993
323	0.003096	22.03674	3.09271
313	0.003195	85.96978	4.454
298	0.003356	52.47045	3.96025
283	0.003534	8.86355	2.18195
273	0.003663	0.1555	-1.8611

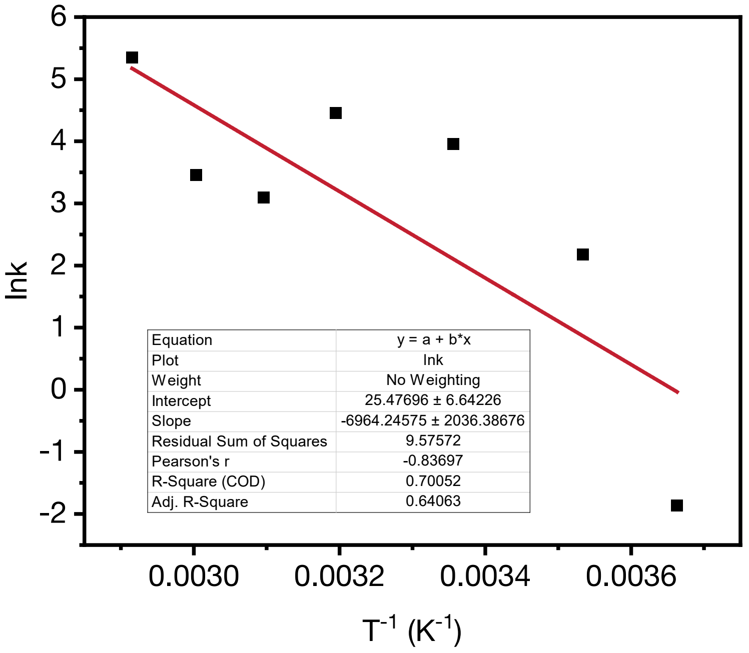

Supplementary Figure 102. Linear fitting for the estimation of the energy barrier for the structural transformation of B(*p*-Cl-C_6H_4)$^-$⊂**1**. As shown in Supplementary Figure 102, by fitting the natural logarithm of the rate constants and the reciprocal of the corresponding temperatures, a linear model was obtained. And according to a rearranged Arrhenius' equation:

$$\ln k = -\frac{E_a}{R}\frac{1}{T} + \ln A \qquad (S12)$$

The slope represents the energy barrier for the structural transformation and is estimated to be 7±2 kJ·mol^{-1}, according to the fitting results.

A similar estimation had been done for *γ*-Chlordane⊂**1**：

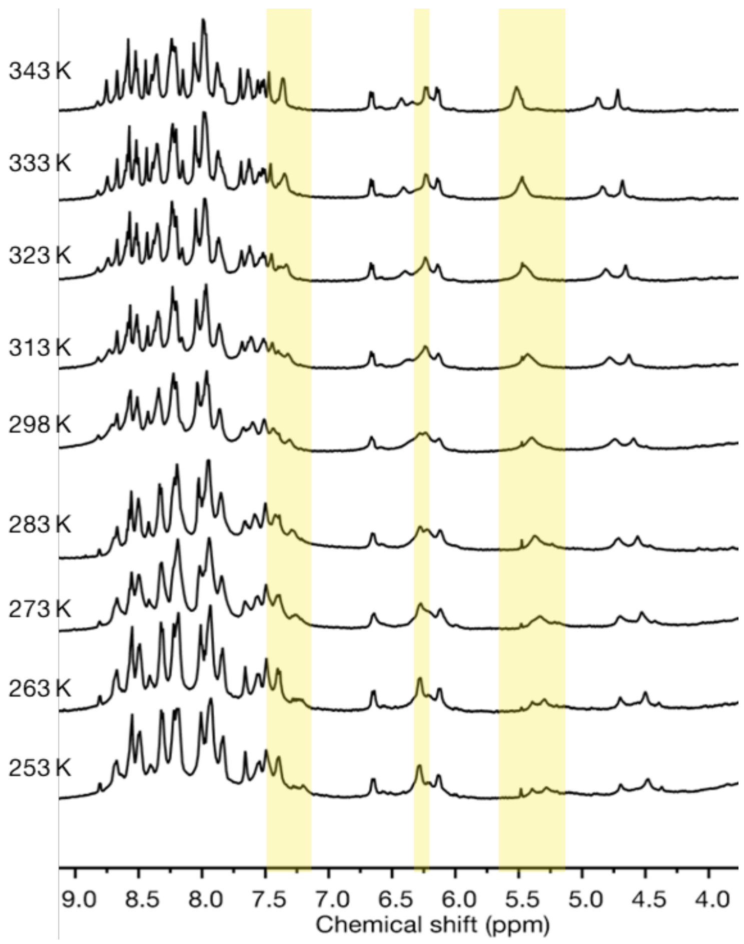

Supplementary Figure 103. Aromatic region of the variable-temperature NMR of *γ*-Chlordane⊂**1** ([**1**] = 1.00 mM, prepared with ca. 10 equiv. of *γ*-Chlordane and purified by washing with Et_2O).

The signal at ~5.37 ppm at 298K was selected as a representative coalescing signal for the estimation. Chemical exchange rate constants for the estimation of the energy barrier for the structural transformation of *γ*-Chlordane⊂**1** are summarized in Supplementary Table 7.

Supplementary Table 7. Chemical exchange rate constants for the estimation of the energy barrier for the structural transformation of γ-Chlordane⊂**1**.

T/K	T^{-1}/K^{-1}	k	lnk
343	0.002915	2899.32553	7.97223
333	0.003003	505.5664	6.22568
323	0.003096	227.57065	5.42746
313	0.003195	192.29678	5.25904
298	0.003356	114.62638	4.74168
283	0.003534	93.52269	4.5382
273	0.003663	61.60057	4.12067
263	0.003802	3.30995	1.19693

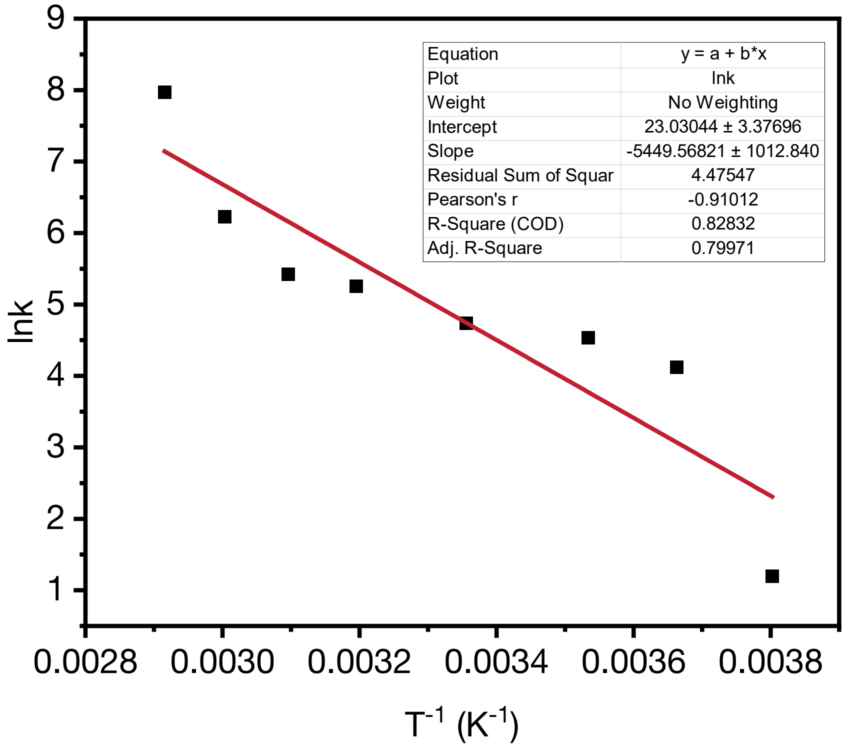

Supplementary Figure 104. Linear fitting for the estimation of the energy barrier for the structural transformation of γ-Chlordane⊂**1**.

The slope represents the energy barrier for the structural transformation and is estimated to be 5±1 kJ·mol^{-1}, according to the fitting results. Based on the results above, the energy barrier for the structural conversion of **1** thus estimated to be between 5±1 and 7±2 kJ·mol^{-1}, corresponding to an overall range of 4-9 kJ·mol^{-1}. This value is comparable to the energy change of most individual rearrangements in the

calculated pathway, as summarised in Supplementary Figure 5, but lower than the energy change of some steps with the highest energy barriers. As the conversion between different conformations of cage **1** was observed to be in fast exchange at room temperature in all cases, we infer that the presence of guest molecules is an important factor that stabilizes the intermediate states and lowers the energy barriers for these transitions.

7 References

1. H. Wadell. *J. Geol.* **1935**, *43*, 250–280.

2. D. Allan, H. Nowell, S. Barnett, M. Warren, A. Wilcox, J. Christensen, L. Saunders, A. Peach, M. Hooper, L. Zaja, S. Patel, L. Cahill, R. Marshall, S. Trimnell, A. Foster, T. Bates, S. Lay, M. Williams, P. Hathaway, G. Winter, M. Gerstel, R. Wooley, *Crystals* **2017**, *7*, 336;

3. P. Evans, *Acta Cryst.* **2006**, *D62*, 72–82;

4. G. Winter, *J. Appl. Crystallogr.* **2010**, *43*, 186–190;

5. G. Winter, D. G. Waterman, J. M. Parkhurst, A. S. Brewster, R. J. Gildea, M. Gerstel, L. Fuentes, Montero, M. Vollmar, T. Michels-Clark, I. D. Young, N. K. Sauter, G. Evans, *Acta Cryst.* **2018**, *D74*, 85–97;

6. O.V. Dolomanov, L.J. Bourhis; R.J. Gildea; J.A.K. Howard, H. Puschmann, *J. Appl. Cryst.*, 2009, **42**, 339-341.

7. P. R. Evans, G. N. Murshudov, *Acta Cryst.* **2013**, *D69*, 1204–1214;

8. M. D. Winn, C. C. Ballard, K. D. Cowtan, E. J. Dodson, P. Emsley, P. R. Evans, R. M. Keegan, E. B. Krissinel, A. G. W. Leslie, A. McCoy, S. J. McNicholas, G. N. Murshudov, N. S. Pannu, E. A. Potterton, H. R. Powell, R. J. Read, A. Vagin, K. S. Wilson, *Acta Cryst.* **2011**, *D67*, 235–242;

9. G. M. Sheldrick, *Acta Cryst.* **2015**, *A71*, 3–8;

10. G. M. Sheldrick, *Acta Cryst.* **2015**, *C71*, 3–8;

11. O. S. Smart, A. Sharff, J. Holstein, T. O. Womack, C. Flensburg, P. Keller, W. Paciorek, C. Vonrhein, G. Bricogne, *Grade2 version 1.3.0.* Global Phasing Ltd.: Cambridge, United Kingdom, 2021.

12. O. S. Smart, T. O. Womack, *Grade Web Server*. Global Phasing Ltd.: 2014.

13. P. van der Sluis, A. L. Spek, BYPASS: An Effective Method for the Refinement of Crystal Structures Containing Disordered Solvent Regions. *Acta Cryst.* **1990**, *A46*, 194–201.

14. A. L. Spek, , PLATON: A Multipurpose Crystallographic Tool. Utrecht University: Utrecht, The Netherlands, 2008.

15. J. B. Maglic, R. Lavendomme, *J. Appl. Cryst.*, **2022**, *55*, 1033-1044.

16. H. Wadell, Volume, Shape, and Roundness of Quartz Particles. *J. Geol.* **1935**, *43*, 250–280.

17. Bindfit v0.5 (Open Data Fit, http://app.supramolecular.org/bindfit/.

18. P. Thordarson, *Chem. Soc. Rev.*, **2011**, 40, 1305-1323.

19. B. T. Ruotolo, J. L. P. Benesch, A. M. Sandercock, S.-J. Hyung, C. V. Robinson, *Nat. Protoc.*, **2008**, *3*, 1139-1152;

20. M. F. Bush, Z. Hall, K. Giles, J. Hoyes, C. V. Robinson, B. T. Ruotolo, *Anal. Chem.*, **2010**, *82*, 9557-9565;

21. I. Campuzano, M. F. Bush, C. V. Robinson, C. Beaumont, K. Richardson, H. Kim, H. I. Kim, *Anal. Chem.*, **2012**, *84*, 1026-1033.

22. F. Neese, *Wiley Interdiscip. Rev.: Comput. Mol. Sci.*, **2022**, *12*, e1606;

23. C. Bannwarth, E. Caldeweyher, S. Ehlert, A. Hansen, P. Pracht, J. Seibert, S. Spicher, S. Grimme, *Wiley Interdiscip. Rev.: Comput. Mol. Sci.*, **2021**, *11*, e1493;

24. M. Bursch, H. Neugebauer, S. Grimme, *Angew. Chem. Int. Ed.*, **2019**, *58*, 11078–11087;

25. N. L. Allinger, Y. H. Yuh, J. H. Lii, *J. Am. Chem. Soc.*, **1989**, 111 (23), 8551–8566;

26. J. H. Lii, N. L. Allinger, *J. Am. Chem. Soc.*, **1989**, 111 (23), 8566–8575;

27. J. H. Lii, N. L. Allinger, *J. Am. Chem. Soc.*, **1989**, 111 (23), 8576–8582;

28. SCIGRESS version FJ 2.6 (EU 3.1.9) build 5996.8255.20141202 (Fujitsu Limited, 2013). https://www.fujitsu.com/global/solutions/business-technology/tc/sol/scigress/ (accessed 2023-09-05);

29. C. Bannwarth, S. Ehlert, S. Grimme, *J. Chem. Theory Comput.*, **2019**, *15*, 1652–1671.

30. S. Spicher, S. Grimme, *Angew. Chem. Int. Ed.,* **2020***, 59*, 15665-15673.

31. PATHSAMPLE: A program for generating connected stationary point databases and extracting global kinetics," http://www-wales.ch.cam.ac.uk/software.html (accessed 7 May 2024).

32. OPTIM: A program for geometry optimisation and pathway calculations," http://www-wales.ch.cam.ac.uk/software.html (accessed 7 May 2024).

33. D. J. Wales, M. A. Miller, T. R. Walsh, *Nature,* **1998**, *394*, 758.

34. O.M. Becker, M. Karplus, *J. Chem. Phys.*, **1997**, *106*, 1495–1517.

35. S. Grimme, A. Hansen, S. Ehlert, J.-M. Mewes, *J. Chem. Phys.,* **2021**, *154,* 064103.

36. P. Pracht, S. Grimme, C. Bannwarth, F. Bohle, S. Ehlert, G. Feldmann, J. Gorges, M. Müller, T. Neudecker, C. Plett, S. Spicher, P. Steinbach, P. A. Wesołowski, F. Zeller, *J. Chem. Phys.,* **2024**, *160,* 114110.

37. S. Ehlert, M. Stahn, S. Spicher, S. Grimme, *J. Chem. Theory Comput.*, **2021**, *17*, 4250-4261.
